# Genetic basis of heterosis for yield and yield components explored by QTL mapping across four genetic populations in upland cotton

**DOI:** 10.1186/s12864-018-5289-2

**Published:** 2018-12-12

**Authors:** Cong Li, Tianlun Zhao, Hurong Yu, Cheng Li, Xiaolei Deng, Yating Dong, Fan Zhang, Yi Zhang, Lei Mei, Jinhong Chen, Shuijin Zhu

**Affiliations:** Department of Agronomy, Zhejiang University, Zhejiang, 310058 Hangzhou China

**Keywords:** Immortalized F_2_ population, Backcross population, Hybrid, Inbreeding depression, Over-dominance, Epistasis

## Abstract

**Background:**

Quantitative trait loci (QTL) mapping provides a powerful tool to unravel the genetic bases of cotton yield and its components, as well as their heterosis. In the present study, the genetic basis underlying inbreeding depression and heterosis for yield and yield components of upland cotton was investigated in recombinant inbred line (RIL), immortalized F_2_ (IF_2_), and two backcross (BCF_1_) populations based on a high-density SNP linkage map across four environments.

**Results:**

Significant inbreeding depression of fruit branches per plant (FB), boll numbers per plant (BN), seed cotton yield (SY), and lint yield (LY) in RIL population and high levels of heterosis for SY, LY, and boll weight (BW) in IF_2_ and two BCF_1_ populations were observed. A total of 285 QTLs were identified in the four related populations using a composite interval mapping approach. In the IF_2_ population, 26.60% partially dominant (PD) QTLs and 71.28% over-dominant (OD) QTLs were identified. In two BCF_1_ populations, 42.41% additive QTLs, 4.19% PD QTLs, and 53.40% OD QTLs were detected. For multi-environment analysis, phenotypic variances (PV) explained by e-QTLs were higher than those by m-QTLs in each of the populations, and the average PV of m-QTLs and e-QTLs explained by QTL × environment interactions occupied a considerable proportion of total PV in all seven datasets.

**Conclusions:**

At the single-locus level, the genetic bases of heterosis varied in different populations. Partial dominance and over-dominance were the main cause of heterosis in the IF_2_ population, while additive effects and over-dominance were the main genetic bases of heterosis in two BCF_1_ populations. In addition, the various genetic components to heterosis presented trait specificity. In the multi-environment model analysis, epistasis was a common feature of most loci associated with inbreeding depression and heterosis. Furthermore, the environment was a critical factor in the expression of these m-QTLs and e-QTLs. Altogether, additive effects, over-dominance, epistasis and environmental interactions all contributed to the heterosis of yield and its components in upland cotton, with over-dominance and epistasis more important than the others.

**Electronic supplementary material:**

The online version of this article (10.1186/s12864-018-5289-2) contains supplementary material, which is available to authorized users.

## Background

Inbreeding depression, the reduced fitness of progenies arising from increased homozygosity [1, 2], and heterosis, wherein cross-fertilization hybrids between diverse varieties or different species exhibit superiority relative to parental performance [3], are fundamentally concerned with inbreeding and outbreeding. Inbreeding depression and heterosis are considered two aspects of the same phenomenon, and both have fundamental importance to applied genetics and breeding. In all cases, the reason for the inbreeding depression is that the inbreeding increases the probability of the homozygosity of deleterious recessive alleles in their progenies [4–6]. The vigor lost caused by consequence of inbreeding can be recovered by crossing [6]. Moreover, inbreeding depression may have a large impact on the formation of reproductive disorders between species, while heterosis may play a key role in maintaining genetic variation of populations [7].

In agriculture, the application of heterosis has contributed greatly to the production of many crops. However, the genetic basis of heterosis remains obscure. Three major hypotheses, including dominance [8, 9], over-dominance [10–12], and certain types of epistasis [13–15] have been proposed to explain heterosis. Quantitative trait locus (QTL) mapping studies in major crops have been performed to explain the genetic basis of heterosis. An appropriate experimental design for the genetic dissection of heterosis is essential. Comstock and Robinson [16] devised the North Carolina design III (Design III) mating scheme, which was the first use of backcross designs to analyze the genetic basis of heterosis. Based on two maize backcross F_3_ families (BCF_3_, a modified Design III scheme), Stuber et al. [12] reported that over-dominance was the major genetic basis of heterosis for grain yield. A study of Xiao et al. [9] investigated the genetic bases of heterosis in two rice BC_1_F_7_ populations and concluded that dominance complementation was the major cause. Li et al. [15] and Luo at al. [17] reported that epistasis and over-dominance were the main causes of inbreeding depression and heterosis of grain yield, grain yield components, and biomass in five related rice mapping populations. By re-analyzing the data of maize [12] and rice [9], Garcia et al. [18] reported that dominance was the main contributor of heterosis in maize, while additive × additive epistatic interactions could be the major genetic basis of heterosis in rice. Schön et al. [19] compared QTL mapping results of three previous Design III studies [12, 20, 21] by advanced statistical methods [22]. Their results indicated that the positive interactions of alleles from the opposite heterotic pool would lead to high heterosis for grain yield of maize. Shang et al. [23] investigated the yield heterosis of upland cotton with two BCF_1_ populations, which implied partial dominance and over-dominance were the main genetic bases. To dissect the heterotic effects directly, Hua et al. [24, 25] introduced an “immortalized F_2_” (IF_2_) population derived from pair crosses of RILs of rice and focused on detecting heterotic loci (HL) to explain the genetic basis of heterosis instead of using traditional QTLs. Based on this design, they discovered that single-locus heterotic effects and dominance × dominance (DD) interactions could explain the genetic basis of heterosis in hybrid rice. Zhou et al. [26] detected several HLs for yield and its components in a rice IF_2_ population and found that the relative contributions of the genetic components varied with traits. Based on a maize IF_2_ population, Tang et al. [27] demonstrated that dominance effects of HL as well as additive × additive interactions were the major genetic bases of heterosis for grain yield and its components. Using the same material, Guo et al. [28] re-analyzed yield heterosis using a reconstructed high-density linkage map. They found that dominance was more important for heterosis than other genetic effects. Moreover, over-dominance and epistasis also contributed to heterosis.

IF_2_ and BCF_1_ populations are ideal materials for comprehensively dissecting the composition of heterosis. Firstly, the genotypes of IF_2_ and BCF_1_ populations can be clearly deduced from the original parents and RILs; secondly, these two populations permit replicated trials; lastly, most loci of IF_2_ and BCF_1_ populations are heterozygous. This provides an opportunity for mapping HL and studying heterosis, rather than analyzing solely based on measuring performance of the trait. It is well known that stably expressed QTLs across multiple environments are deeply favored in marker-assisted selection (MAS). Thus, identifying QTLs and exploring their expression levels and the genetic basis of heterosis under multiple environments in related populations would allow us to map stable QTLs and accelerate the process of breeding.

Upland cotton is the most important natural textile fiber source globally. Currently, it is grown on a total area of 30.9 million ha of land in more than 80 countries [29]. It is urgent to improve the yield of upland cotton cultivars to meet worldwide demand, and maintain profitability for cotton growers. Yield is a complex trait in cotton that is controlled by a large number of QTLs. Several studies have discovered that significant heterosis for yield and yield components exists in upland cotton [30–32]. In addition, some QTL mapping studies have been reported that dissect the composition of heterosis for yield and yield components of upland cotton [23, 32, 33], but no studies have been reported on different segregating populations from the same parental combination. In the present study, RIL, IF_2_, and two BCF_1_ populations were used simultaneously to perform QTL genetic analysis for yield and yield components based on a high-density SNP intraspecific genetic map under multiple environmental conditions. The results will provide meaningful hints at the underlying genetic bases of inbreeding depression and heterosis for yield and yield components, which can be used in cotton breeding.

## Methods

### Plant materials

**Fig. 1:**
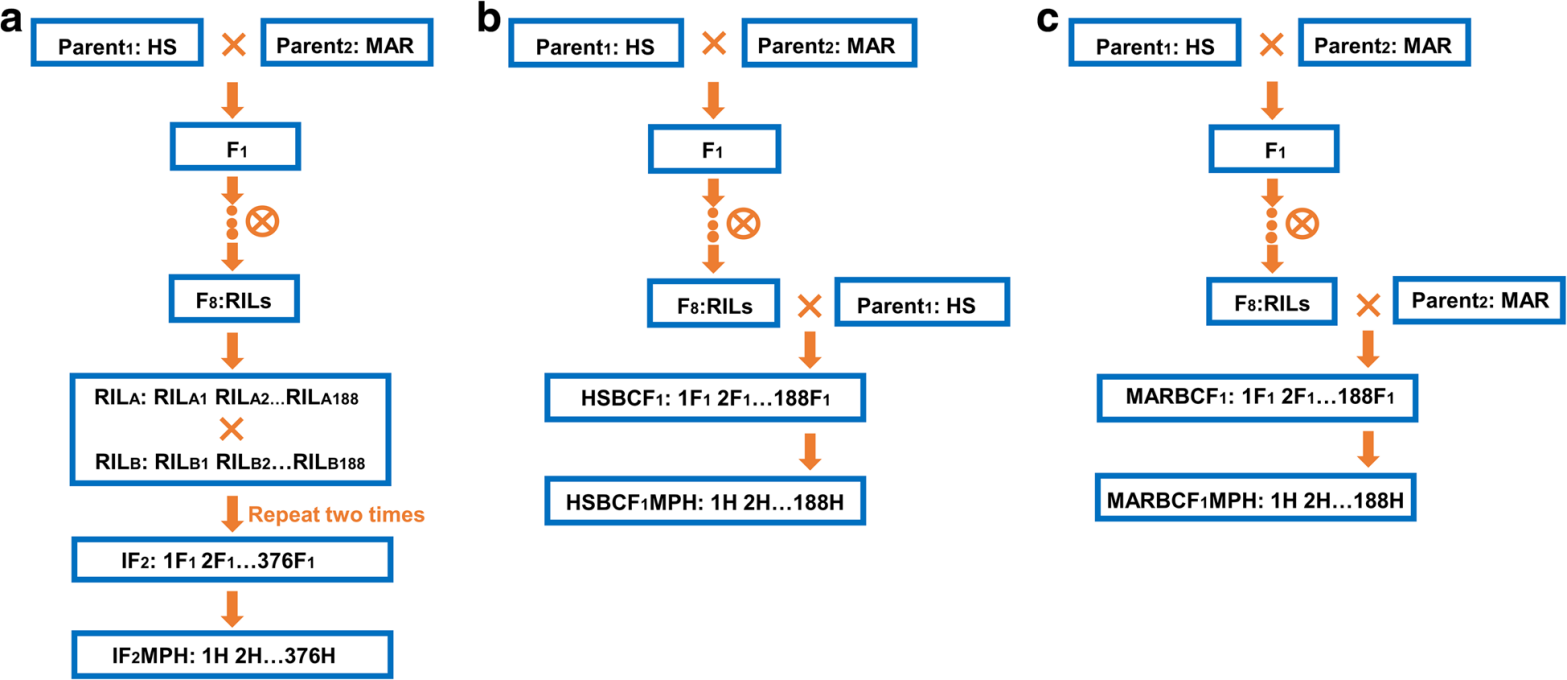
Diagram of the development scheme for the RIL, IF_2_, and two BCF_I_ populations. **a** Crosses were made between two different lines from RIL_A_ and RIL_B_ (for example, RIL_A1_ × RIL_B2_). Here, RIL_A_ and RIL_B_ represent female and male RIL lines, respectively. This procedure was repeated two times, and finally 376 lines were produced, forming the IF_2_ population. The IF_2_MPH dataset derived from the IF_2_ population included 376 mid-parental heterosis (MPH) values (H), which were estimated by MPH = F_1_ – MP (Hua et al. 2003). Here, F_1_ represents the observations in the IF_2_ populations, and MP represents the average trait value between their parents. **b**, **c** The HSBCF_1_ and MARBCF_1_ populations were produced by RILs × P_1_ and RILs × P_2_, respectively, and each contained 188 lines. The RILs were used as the female parents in two backcross designs with the two original parents. The HSBCF_1_MPH and MARBCF_1_MPH datasets also each included 188 individuals (H) and were estimated by MPH = F_1_ – MP (Hua et al. 2003). Here, F_1_ represents the observations in the two BCF_1_ populations, and MP represents the average trait value between their corresponding parents. HS: HS46; MAR: MARCABUCAG8US-1-88

Four related genetic populations were used, including a set of 188 RILs (F_8_), an IF_2_ population, and two BCF_1_ populations (Fig. 1). The RILs were derived by single-seed procedure from a cross between two elite upland germplasms, HS46 (P_1_) and MARCABUCAG8US-1-88 (P_2_). According to a diallel mating design [25], the IF_2_ population was produced from crosses between the RILs chosen by random permutations of the 188 RILs (Fig. 1a). This procedure was repeated two times, with each time making 188 hybrids, forming a population consisting of 376 IF_2_ hybrids. Both BCF_1_ populations were derived from a modified Design III [16, 21], in which two parents were used as the male parents backcrossed with the 188 RILs (Fig. 1b, c). The two BCF_1_ populations each contained 188 hybrids named HSBCF_1_ and MARBCF_1_, referring to HS46 (HS) and MARCABUCAG8US-1-88 (MAR) backcrossed with 188 RIL lines, respectively.

### Field planting and phenotypic evaluation

Two separate experiments were conducted at two locations, Yacheng (inland climate) and Baogang (coastal climate) of Sanya, Hainan Province, China, in the cotton growing seasons of 2014 and 2015. All plants of the four populations and the two parental lines were planted in a randomized block design with two replications in each location and with 5.6 m^2^ plot areas. Finally, 29 plants were grown in each row at a spacing of 25 cm between plants. Standard cultivation, weed and insect control practices were performed as the management of the local cotton production.

Ten consecutive plants in the middle of each row were tagged for trait measurement [32, 34]. Data were collected for the following traits: seed cotton yield (SY), lint yield (LY), fruit branches per plant (FB), boll numbers per plant (BN), boll weight (BW), and lint percentage (LP). During the harvest season, twenty fully open bolls in each row were harvested for measurement of BW and LP. SY was determined as the seed cotton weight harvested from each plot and LY was determined by multiplying lint percentage with SY.

### Genotype analysis and linkage maps

Young leaves were collected from RILs and two parents. Individual genomic DNA was extracted following a modified CTAB method [35].

The RIL population and two parents were genotyped with the cotton 63 K SNP array [36]. A total of 63,058 SNPs were screened for polymorphism between parents, in which a total of 2618 SNP markers were selected to genotype the RILs [37]. In the IF_2_ and two BCF_1_ populations, the genotypes for each F_1_ were deduced from the RILs and the parental lines that were used as the parents for each cross.

### Data analysis

Each year-location was treated as an independent environment. A descriptive statistics model was used to test the basic statistics of phenotypic data for RILs, IF_2_s, HSBCF_1_s, and MARBCF_1_s. One-way analysis of variance (ANOVA) was performed to analyze the difference for yield and yield components between the two parents using SPSS 20.0. Broad-sense heritability was estimated as *H*^2^ = *V*_G_ / (*V*_*G*_ + *V*_*GE*_ */ e* + *V*_*ε*_ */ re*), where *H*^2^ is broad-sense heritability, *V*_G_ = genetic variance, *V*_*GE*_ = genotype × environment interaction variance, *V*_ε_ = error variance, and *e* and *r* are the numbers of environments and replicates, respectively. The *V*_*G*_, *V*_*GE*_, and *V*_ε_ variances were calculated using the minimum norm quadratic unbiased estimation (MINQUE) approach [38] by in QGA Station 2.0 (http://ibi.zju.edu.cn/software/qga/index.htm).

The hybrid breakdown value (HB), a component of inbreeding depression [39, 40], was calculated for individual RILs as follows: HB = RIL− MP, where MP = (HS46 + MARCABUCAG8US-1-88) / 2. The equation for calculating values of the mid-parental heterosis (MPH) of individual IF_2_, HSBCF_1_, and MARBCF_1_ hybrids for yield traits was as follows: MPH = F_1_ – MP [25], where F_1_ was the mean value of each hybrid in the IF_2_, HSBCF_1_, and MARBCF_1_ populations, and MP was the average value of the corresponding parents. The MPH datasets were used as the raw data for exploring the genetic basis of yield heterosis.

WinQTL Cartographer 2.5 [41] was used to identify single-locus QTLs with the composite interval mapping (CIM) method. The LOD threshold for declaring a significant QTL was calculated by 1000 permutation tests with a mapping step of 1.0 cM and a significance level of *P* < 0.05. The MPH datasets only detected the dominance effect under the genetic model of CIM, where the QTL exhibited a significant difference between the heterozygote and the mean of the two parental homozygotes. QTLs were named as: q + trait abbreviation + chromosome number + QTL number [37]. A graphical representation of the linkage map with QTLs marked was created using Map Chart 2.2 [42].

The gene actions in different datasets were estimated as follows: *a* = (P_1_P_1_ − P_2_P_2_) / 2, *d* = (P_1_P_2_ − (P_1_P_1_ + P_2_P_2_) / 2), BCF_1_ = (*a* + *d*), where P_1_ and P_2_ represent the parents, P_1_P_1_ and P_2_P_2_ indicate the effects of homozygous genotypes observed in RILs, IF_2_s, and BCF_1_s; and P_1_P_2_ represents the effects of heterozygous genotypes in hybrid. The gene action mode for each QTL was calculated by the absolute value of the ratio of dominant and additive effects (|*d*/*a*|) [26, 35, 36, 43]. There were some differences between the assessment in IF_2_ and BCF_1_ populations. For the IF_2_ population, QTLs with |*d*/*a*| ≤ 1 were considered as completely or partially dominant (D or PD) loci. If |*d*/*a*| > 1 or if it was only detectable for the MPH dataset, the QTL was referred to as an over-dominance (OD) locus. The |*d*/*a*| value was estimated in two ways; both *a* and *d* were estimated from IF_2_s for a QTL which only detected in IF_2_s; *a* was from RILs and *d* was from the MPH dataset for a QTL detected simultaneously in RILs and the IF_2_MPH dataset. Moreover, the value of |*d*/*a*| in IF_2_s was used as the criterion. For BCF_1_ populations, a QTL was considered to be an OD locus in the following three cases: (1) MPH (*d*) times two was higher than BCF_1_ performance (*a* + *d*) i.e., 2 |*d*| (MPH) > |*a* + *d*| (BCF_1_) (equal to |*d*/*a*| > 1) for a QTL detected in BCF_1_s and the MPH dataset; (2) *a* was estimated from RILs and d from the MPH dataset with |*d*/*a*| > 1 for a QTL detected simultaneously in the RILs and MPH dataset; (3) only detected in MPH dataset. Otherwise, the QTL was considered to be a D or PD locus. QTLs detected only in BCF_1_ were referred to as additive (A) loci. When a QTL was present in all three datasets, the judgment depended on the ratio of the effects in the BCF_1_s and MPH dataset.

A combined multi-environment model analysis that tests the main-effect QTLs (m-QTLs), epistatic QTLs (e-QTLs), and their environmental interactions (QTL × environment, QE) was implemented using the RILs, IF_2_s, two BCF_1_s, and three MPH datasets with the inclusive composite interval mapping (ICIM) method in IciMapping 4.1 [44]. The pre-set parameters Scan = 1 cM / PIN = 0.0001 and Scan = 5 cM / PIN = 0.0001 were used to conduct the additive and epistasis QTL mapping analyses, respectively. The threshold LOD for declaring m-QTLs and e-QTLs was calculated using a permutation test at a significance level of *P* < 0.05, *n* = 1000. The identified m-QTLs were named using the dataset abbreviation followed by “maq” (multi-environment additive QTL), and then suffixed with the abbreviation of trait and chromosome number, followed by the QTL number. The e-QTLs detected were named using the dataset abbreviation followed by “meq” (multi-environment epistatic QTL), and then with the abbreviation of the trait and the QTL pair number. Datasets were abbreviated was as follows: “R”, “I”, “B_1_”, and “B_2_” represent RILs, IF_2_s, HSBCF_1_s, and MARBCF_1_s, respectively, and “M” was added after the last three heterozygous population abbreviation to represent their corresponding MPH datasets, i.e., “IM”, “B_1_M”, and “B_2_M”.

## Results

### Inbreeding depression and heterosis for yield and yield components

The phenotypic variation for yield and its components among the parents, RIL, IF_2_, and two BCF_1_ populations, as well as the estimated HB of RILs and the MPH of the IF_2_s and two BCF_1_s are shown in Table 1 and Table 2, respectively. The female parent, HS46, had significantly greater trait values for FB, BN, BW, SY, and LY than those of MARCABUCAG8US-1-88 in all environments (Additional file 1: Table S1). A wide range of variation was observed in yield and its components in the RILs, IF_2_s, HSBCF_1_s, and MARBCF_1_s (Table 1). In all environments, obvious reductions of the RILs were observed as a result of hybrid breakdown in the traits of FB, BN, SY, and LY (Table 2, Additional file 2: Table S2). The mean deviation of the RILs from the midparental values for LP was found in three environments (but not in 2015Bg), while it was only detected in one environment for BW. High levels of heterosis for SY, LY, and BW were observed in the IF_2_ and two BCF_1_ populations. However, other yield components showed lower levels of heterosis in these three populations.

**Table 1:** Phenotypic variation of yield and yield components

Traits ^a^	Env. ^b^	Parents ^c^		RILs			IF_2_s			HSBCF_1_s			MARBCF_1_s		
		P_1_	P_2_	Mean	Min	Max	Mean	Min	Max	Mean	Min	Max	Mean	Min	Max
FB	2014Yc	8.93	7.62	8.25	5.68	11.20	8.29	3.00	12.00	8.67	5.25	11.40	8.25	5.40	11.00
	2014Bg	8.71	7.80	8.25	5.05	10.50	8.02	4.00	11.00	8.34	4.67	11.50	8.10	4.00	14.20
	2015Yc	9.53	9.50	9.05	7.00	12.00	9.83	6.00	14.00	10.76	8.40	13.80	11.00	8.50	14.00
	2015Bg	9.41	9.34	9.07	7.00	13.00	9.84	7.00	14.00	10.73	7.20	13.60	10.96	8.80	24.80
BN	2014Yc	17.22	12.82	14.52	8.95	26.33	13.65	6.00	27.00	14.52	7.50	25.60	14.20	7.00	24.80
	2014Bg	15.21	13.02	13.45	7.93	22.83	12.50	4.00	22.00	13.40	6.33	24.50	12.96	6.00	21.00
	2015Yc	11.64	11.37	11.30	8.00	17.00	10.95	5.00	30.00	10.87	6.80	20.20	11.58	6.60	19.60
	2015Bg	11.57	11.47	11.00	7.00	18.00	10.85	5.00	32.00	10.70	6.60	20.40	11.37	7.40	19.20
BW	2014Yc	5.43	4.91	5.36	3.96	7.09	6.48	5.11	7.78	6.62	3.46	7.99	6.47	3.53	8.22
	2014Bg	5.81	5.37	5.58	3.92	7.27	6.40	4.36	7.83	6.23	4.54	7.95	6.52	5.28	8.71
	2015Yc	5.70	5.38	5.94	4.56	7.19	6.25	4.20	7.93	6.03	4.00	7.47	6.16	4.06	7.49
	2015Bg	5.93	5.53	5.75	3.91	7.02	5.81	3.93	7.19	5.77	4.10	7.33	5.77	3.96	7.55
LP	2014Yc	37.07	38.74	37.70	31.63	43.27	38.17	32.40	44.80	37.89	31.72	41.93	38.03	32.01	45.69
	2014Bg	39.07	39.87	38.24	33.11	42.15	39.80	32.03	48.24	38.72	34.39	42.51	39.43	30.08	44.60
	2015Yc	36.80	37.48	33.38	25.42	41.96	36.78	30.62	46.29	36.84	32.05	40.92	36.96	32.73	41.46
	2015Bg	35.22	37.51	36.55	30.96	43.03	36.97	31.42	49.89	36.16	29.11	44.78	36.48	32.29	42.15
SY	2014Yc	653.07	500.98	536.19	222.51	1094.62	799.83	246.34	1630.69	741.73	273.78	1201.44	771.05	304.27	1519.42
	2014Bg	688.61	524.50	572.03	233.77	1070.01	784.51	311.78	1618.95	763.67	327.05	1495.06	776.61	317.56	1381.54
	2015Yc	689.56	637.48	639.04	367.37	1165.21	662.18	380.12	1282.85	714.35	454.95	1085.23	687.21	429.33	1009.60
	2015Bg	710.93	665.00	606.15	389.04	884.08	631.93	323.26	1147.04	699.53	339.48	1113.20	675.57	458.16	1088.63
LY	2014Yc	242.84	194.54	201.93	80.45	402.32	290.91	91.55	621.65	281.23	102.99	490.98	293.26	122.74	603.94
	2014Bg	268.71	208.84	219.07	85.43	420.59	312.89	127.68	634.61	295.96	125.72	602.08	306.24	125.09	576.80
	2015Yc	243.07	237.53	235.13	131.55	444.15	241.60	122.38	449.10	263.16	156.13	390.22	253.96	148.76	383.77
	2015Bg	250.27	248.44	221.54	135.12	330.12	232.84	113.83	461.05	252.89	159.12	383.85	246.65	159.12	383.85

^a^*FB* fruit branches per plant, *BN* boll numbers per plant, *BW* boll weight, *LP* lint percentage, *SY* seed cotton yield, *LY* lint yield

^b^2014Yc: Yacheng, Hainan Province in 2014; 2014Bg: Baogang, Hainan Province in 2014; 2015Yc: Yacheng, Hainan Province in 2015; 2015Bg: Baogang, Hainan Province in 2015

^c^P_1_: HS46; P_2_: MARCABUCAG8US-1-88

**Table 2:** Summary statistics on HB^a^ percentage of RILs and MPH^a^ percentage of IF_2_s, HSBCF_1_s, and MARBCF_1_s

Traits ^b^	Env. ^c^	RILHBs (%)			IF_2_MPHs (%)			HSBCF_1_MPHs (%)			MARBCF_1_MPHs (%)		
		Mean	Min	Max	Mean	Min	Max	Mean	Min	Max	Mean	Min	Max
FB	2014Yc	−0.30	−31.42	35.35	0.81	−42.23	58.10	2.12	−38.00	53.16	5.12	−40.79	92.91
	2014Bg	−0.06	−38.80	27.24	−1.82	−47.02	50.79	−1.39	−40.00	58.10	1.85	−54.72	50.88
	2015Yc	−5.26	−26.42	20.88	9.09	−28.81	65.19	24.02	−17.60	82.42	19.75	−16.19	77.48
	2015Bg	−3.83	−28.56	36.22	8.83	−30.76	60.00	22.96	−24.61	74.36	20.77	−11.57	68.55
BN	2014Yc	−3.38	−40.43	75.28	−4.17	−60.78	123.64	−6.84	−54.26	117.56	6.92	−42.77	97.02
	2014Bg	−4.69	−43.85	61.79	−5.48	−75.94	83.63	−5.27	− 52.32	98.38	1.49	−51.19	84.91
	2015Yc	−2.21	−31.34	43.40	1.39	−54.55	169.68	4.69	−42.54	99.01	7.94	−39.35	80.71
	2015Bg	−5.03	−40.96	53.24	3.26	−47.83	195.35	7.75	−46.81	108.16	8.76	− 45.54	75.00
BW	2014Yc	3.57	−23.38	37.04	20.94	−13.49	46.43	22.46	−33.54	53.51	26.72	−32.55	84.25
	2014Bg	−0.13	−29.83	29.99	14.88	−21.42	50.79	9.21	−22.39	44.58	19.32	−14.11	59.64
	2015Yc	7.24	−17.57	29.86	5.53	−26.19	38.07	3.96	−34.60	38.65	9.06	−27.50	44.49
	2015Bg	0.30	−31.74	22.56	1.88	−39.57	60.79	0.44	−28.90	50.64	2.82	−30.24	50.49
LP	2014Yc	−0.54	−16.54	14.15	1.49	− 12.73	15.68	1.28	−14.78	21.55	−0.56	−17.03	19.13
	2014Bg	−3.12	−16.12	6.79	4.50	−18.08	22.81	0.62	−9.71	13.43	1.03	−24.67	16.02
	2015Yc	−10.13	−31.55	12.99	12.60	−22.61	70.98	6.14	−12.10	25.96	3.98	−16.26	25.57
	2015Bg	0.49	−14.87	18.32	1.43	−19.06	37.09	0.37	−18.30	30.21	−1.33	−16.73	16.19
SY	2014Yc	−7.08	−61.44	89.70	51.96	−61.43	195.90	25.01	−49.55	144.85	51.39	−49.74	137.63
	2014Bg	−5.69	−61.46	76.41	38.94	−56.93	148.05	22.05	−51.34	166.60	44.37	−40.61	115.56
	2015Yc	−3.69	−44.63	75.61	4.74	−41.94	104.71	6.68	−45.56	95.99	7.64	−47.25	96.22
	2015Bg	−11.89	−43.45	28.51	5.97	−43.89	69.82	6.32	−52.10	91.46	6.87	− 41.73	95.69
LY	2014Yc	−7.66	−63.21	83.97	46.73	−61.63	196.96	26.74	−47.72	166.46	50.53	−49.29	142.61
	2014Bg	−8.25	−64.22	76.15	45.28	−54.74	151.20	22.72	−47.82	177.90	46.02	−37.47	120.89
	2015Yc	−2.15	−45.26	84.83	4.15	−53.62	93.64	8.85	−47.44	95.35	7.32	−46.75	88.85
	2015Bg	−11.15	−45.81	32.39	7.19	−44.54	103.30	6.74	−52.48	98.55	5.39	−40.08	91.87

^a^HB: hybrid breakdown value; MPH: mid-parental heterosis value

^b^*FB* fruit branches per plant, *BN* boll numbers per plant, *BW* boll weight, *LP* lint percentage, *SY* seed cotton yield, *LY* lint yield

^c^2014Yc: Yacheng, Hainan Province in 2014; 2014Bg: Baogang, Hainan Province in 2014; 2015Yc: Yacheng, Hainan Province in 2015; 2015Bg: Baogang, Hainan Province in 2015

Different levels of heterosis were found among the different populations across four environments (Additional file 2: Table S2). For SY, IF_2_ and MARBCF_1_ populations showed the same levels of heterosis, at 24.75 and 28.61%, respectively, which were higher than that of the HSBCF_1_ population (15.42%). The mean levels of heterosis of for the LY trait showed the same trend as SY in different populations. For BW, different populations have similar levels of heterosis, although it was slightly higher in the MARBCF_1_ population. For FB, the two BCF_1_ populations showed the same levels of heterosis, and IF_2_s exhibited lower mean heterosis. For BN, the mean levels of heterosis in the MARBCF_1_ population were higher than that of the HSBCF_1_ population, while the IF_2_ population exhibited negative heterosis (− 2.70%). For LP, the order of the mean values in the MPH datasets was IF_2_MPH > HSBCF_1_MPH > MARBCF_1_MPH.

There were some differences between environments (Table 2). In all three populations, the MPH (%) of SY was lower in 2015 than that in 2014. The same trend was found for LY and BW, caused by boll rot during experiments due to high rainfall in 2015 in Sanya. However, the heterosis level of FB showed the opposite trend, where higher levels of heterosis were detected in 2015. Moreover, all of the environments showed low levels of heterosis for LP in all populations except for the 2015Yc environment.

Within each population, heterosis values of individual hybrids varied considerably. Most of the trait values of extreme lines showed high MPH in all environments (Additional file 3: Table S3). For example, in 2014Yc and 2014Bg, the mean heterosis values of SY were more than 83% for the 20 highest-heterosis hybrids of the IF_2_, HSBCF_1_, and MARBCF_1_ populations and were more than 46% in the 2015Yc and 2015Bg experiments.

The broad-sense heritability was analyzed using measurement data from four environments (Table 3). All measures of yield and its components showed moderate heritability, ranging from 56.00 to 86.31%, 46.79 to 64.05%, 39.73 to 65.81%, and 41.20 to 67.21% in the RIL, IF_2_, HSBCF_1_, and MARBCF_1_ populations, respectively, which presented significant genetic and environmental effects. LP exhibited nearly the highest heritability and FB the lowest in all populations. Interestingly, the heritability of all traits was highly consistent between the two BCF_1_ populations, which might be related to their closer genetic basis.

**Table 3:** Analysis of variance for yield and yield components across four populations

Population	Components of variation^a^	Traits^b^					
		FB	BN	BW	LP	SY	LY
RIL	*V*_*G*_	0.287	1.340	0.081	1.843	3944.200	638.499
	*V*_*GE*_	0.000	0.000	0.020	0.000	0.002	0.001
	*V*_*e*_	1.804	8.243	0.421	2.338	20,321.300	2956.910
	*H*^*2*^ (%)	56.00	56.54	58.45	86.31	60.83	63.34
IF_2_	*V*_*G*_	0.347	1.775	0.154	1.062	10,632.730	1643.570
	*V*_*GE*_	0.011	0.260	0.017	0.604	2385.370	479.871
	*V*_*e*_	3.139	14.364	0.787	4.924	44,067.000	6421.440
	*H*^*2*^ (%)	46.79	48.82	60.01	58.09	63.53	64.05
HSBCF_1_	*V*_*G*_	0.315	1.589	0.072	1.192	4952.399	785.839
	*V*_*GE*_	0.130	0.744	0.027	0.199	147.787	4.376
	*V*_*e*_	1.694	7.424	0.412	2.277	19,672.600	2875.500
	*H*^*2*^ (%)	40.84	43.77	39.73	65.81	49.99	52.19
MARBCF_1_	*V*_*G*_	0.339	1.585	0.101	1.185	5590.720	835.094
	*V*_*GE*_	0.240	0.938	0.031	0.038	1793.300	169.841
	*V*_*e*_	3.390	14.863	0.824	4.549	44,491.400	5743.140
	*H*^*2*^ (%)	41.20	43.11	47.65	67.21	48.19	52.34

^a^*V*_*G*_ genetic variance, *V*_*GE*_ genotype × environment interaction variance, *Ve* error variance, *H*^*2*^ the broad-sense heritability

^b^*FB* fruit branches per plant, *BN* boll numbers per plant, *BW* boll weight, *LP* lint percentage, *SY* seed cotton yield, *LY* lint yield

The phenotypic correlations among the traits varied greatly in the RIL, IF_2_, and two BCF_1_ populations (Additional file 4: Table S4). This can be illustrated with LY as an example. Consistent with previous reports [45, 46], there were significant positive correlations between LY and SY in all populations, possibly because LY is derived from SY multiplied by lint percentage. Similarly, LY was positively and significantly correlated with BN and BW in all populations, indicating that variation in BN and BW contributed strongly to the variation in LY. The association between LY and LP was significant in three populations except for the RILs, and a significant positive correlation was recovered in two BCF_1_ populations but was significantly negative in the IF_2_ population, indicating that variation in LP contributed differently to LY variation in different populations. However, LY was only significantly positively correlated with FB in the RIL population.

### QTL analysis of yield and yield components in RIL, IF_2_, and two BCF_1_ populations

A genetic linkage map was previously constructed based on the polymorphic loci identified [37] (Additional file 5: Figure S1). A total of 285 QTLs for yield and its components were detected using CIM in the RILs, IF_2_s, two BCF_1_s, and three MPH datasets (Additional file 5: Figure S1, Table 4, Additional file 6: Table S5). Among them, 107 QTLs were identified in more than two environments or datasets.

**Table 4:** Gene actions of QTL identified for yield and yield components by CIM^a^ across four environments

Traits^b^	IF_2 _population				HSBCF_1_ population				MARBCF_1_ population			
	A^c^	PD/D^c^	OD^c^	Uncertain^c^	A	PD/D	OD	Uncertain	A	PD/D	OD	Uncertain
FB	0	3	16	0	2	0	10	0	6	2	3	0
BN	0	8	7	0	6	1	6	0	7	0	7	0
BW	0	3	12	0	10	0	9	0	11	0	4	0
LP	0	5	17	2	7	0	7	0	9	1	8	0
SY	0	2	6	0	5	0	19	0	5	1	11	0
LY	0	4	9	0	4	1	12	0	9	2	6	0

^a^*CIM* composite interval mapping method

^b^*FB* fruit branches per plant, *BN* boll numbers per plant, *BW* boll weight, *LP* lint percentage, *SY* seed cotton yield; LY: lint yield

^c^*A* additive effect, *PD/D* partial dominant or dominant effect, *OD* over-dominant effect; Uncertain: QTL with different gene action in different environments

#### Fruit branches per plant (FB)

A total of 40 QTLs were detected in seven datasets, explaining 3.15–31.66% of the total PV, and ten of them were the stable QTLs that were identified in at least two environments or datasets. Three, six, five, eight, 15, ten, and five QTLs were detected in the RILs, IF_2_s, HSBCF_1_s, MARBCF_1_s, IF_2_MPHs, HSBCF_1_MPHs, and MARBCF_1_MPHs, respectively. In the IF_2_ population, three QTLs with PD or D effects and 16 with OD effects were observed. Two QTLs with PD effects were simultaneously detected in both IF_2_s and IF_2_MPHs. In the HSBCF_1_ population, two QTLs with A effects and ten with OD effects were found. Four QTLs with apparent OD effects were detected in both HSBCF_1_s and HSBCF_1_MPHs. In the MARBCF_1_ population, six QTLs with A effects, two with PD or D effects, and three with OD effects were observed. Two QTLs with OD effects were detected in MARBCF_1_ and its MPH dataset.

#### Boll numbers per plant (BN)

Forty-two QTLs associated with BN were detected in seven datasets. Among those, 17 were detected in more than two environments or datasets. There were six, 11, ten, ten, six, seven, and seven QTLs in the RILs, IF_2_s, HSBCF_1_s, MARBCF_1_s, IF_2_MPHs, HSBCF_1_MPHs, and MARBCF_1_MPHs, respectively. In the IF_2_ population, eight QTLs with PD effects and seven QTLs with OD effects were observed. qBN-C01–2, with PD effect, was detected in both IF_2_s and IF_2_MPHs. In the HSBCF_1_ population, six QTLs with A effects, one QTL with PD or D effect, and six QTLs with OD effects were detected. Among them, four QTLs with OD effects were detected in HSBCF_1_ and its MPH dataset. In the MARBCF_1_ population, seven QTLs with A effects and seven with OD effects were observed. Three QTLs with OD effects were detected in MARBCF_1_ and its MPH dataset. qBN-C06–1 with apparent A effect, and qBN-C17–3, with OD effect, were identified in the two environments of MARBCF_1_s and MARBCF_1_MPHs, respectively. Both of them showed favorable alleles conferred by different parent in their two environments.

#### Boll weight (BW)

A total of 30 QTLs were identified, explaining 11.41% of the mean total PV. Among them, 11 QTLs were identified in more than two environments or datasets. Three, 11, 14, 12, five, nine, and four QTLs were identified in the RILs, IF_2_s, HSBCF_1_s, MARBCF_1_s, IF_2_MPHs, HSBCF_1_MPHs, and MARBCF_1_MPHs, respectively. In the IF_2_ population, three QTLs exhibited PD or D effects, while 12 QTLs with |*d*/*a*| > 1 showed apparent OD effects. qBW-C10–1, with PD effect, was detected in both IF_2_s and IF_2_MPHs. In the HSBCF_1_ population, ten QTLs with A effects and nine with OD effects were detected. Four QTLs with apparent OD effects were detected in both HSBCF_1_s and HSBCF_1_MPHs. In the MARBCF_1_ population, 11 QTLs with A effects and four with OD effects were observed. qBW-C13–4, with OD effect, was detected in both MARBCF_1_s and MARBCF_1_MPHs.

#### Lint percent (LP)

Among 50 identified QTLs related to LP, 27 QTLs were detected in more than two environments or populations. Thirteen, 21, nine, 15, nine, seven, and nine QTLs were detected in the RILs, IF_2_s, HSBCF_1_s, MARBCF_1_s, IF_2_MPHs, HSBCF_1_MPHs, and MARBCF_1_MPHs, respectively. In the IF_2_ population, five QTLs with PD effects and 17 with OD effects were observed. Six QTLs were simultaneously detected in both IF_2_s and IF_2_MPHs. The gene action of two QTLs, qLP-C09–3 and qLP-C25–2, was uncertain because of inconsistent dominance degree in different environments. In the HSBCF_1_ population, seven QTLs with A effects and seven with OD effects were found. Two QTLs with apparent OD effects were detected in both HSBCF_1_s and HSBCF_1_MPHs. In the MARBCF_1_ population, nine QTLs with A effects, one with PD or D effect, and eight with OD effects were observed. Six QTLs were detected in both MARBCF_1_ and its MPH dataset.

#### Seed cotton yield (SY)

Fifty QTLs were identified on 22 chromosomes in the seven datasets, explaining 13.39% (ranging from 3.40 to 34.83%) of the mean total PV. Twenty-three QTLs were identified in more than two environments or datasets. There were eight QTLs detected in IF_2_s and its MPH dataset, among which two QTLs exhibited PD effects and six QTLs showed OD effects. qSY-C18–2, with PD effect, was identified in IF_2_s in 2015Bg and in IF_2_MPHs in 2015Yc and 2015Bg. In HSBCF_1_s and its MPH dataset, five QTLs with A effects and 19 with OD effects were detected. Up to ten QTLs with apparent OD effects were detected in both HSBCF_1_s and HSBCF_1_MPHs. Among them, qSY-C16–1 showed favorable alleles conferred by different parents in the two environments of HSBCF_1_MPHs. In the MARBCF_1_ population, five QTLs with A effects, one with a PD or D effect, and 11 with OD effects were observed. Five QTLs were identified simultaneously in MARBCF_1_ and its MPH dataset.

#### Lint yield (LY)

Forty-seven QTLs, explaining 3.06–34.06% of the total PV, were detected using the seven datasets. In the IF_2_ hybrids, eight QTLs were detected. Four QTLs with PD effects and nine with OD effects were observed in a combined analysis of IF_2_ and its MPH dataset. qLY-C18–1, with PD effect, was detected in both IF_2_s and IF_2_MPHs. In the HSBCF_1_ population, four QTLs with A effects, one with PD effect, and 12 with OD effects were found. Among them, seven QTLs were detected simultaneously in HSBCF_1_s and HSBCF_1_MPHs. In the MARBCF_1_ population, nine QTLs with A effects, two with PD effects, and six with OD effects were observed. Three QTLs were detected simultaneously in MARBCF_1_s and MARBCF_1_MPHs. qLY-C19–2, with PD effect, was identified in MARBCF_1_MPHs in both 2014Yc and 2015Bg, as well as in one environment of the RILs, which showed favorable alleles conferred by different parents in these two environments of MARBCF_1_MPHs.

### Multi-environment analysis of main-effect QTL and environmental interactions

**Fig. 2:**
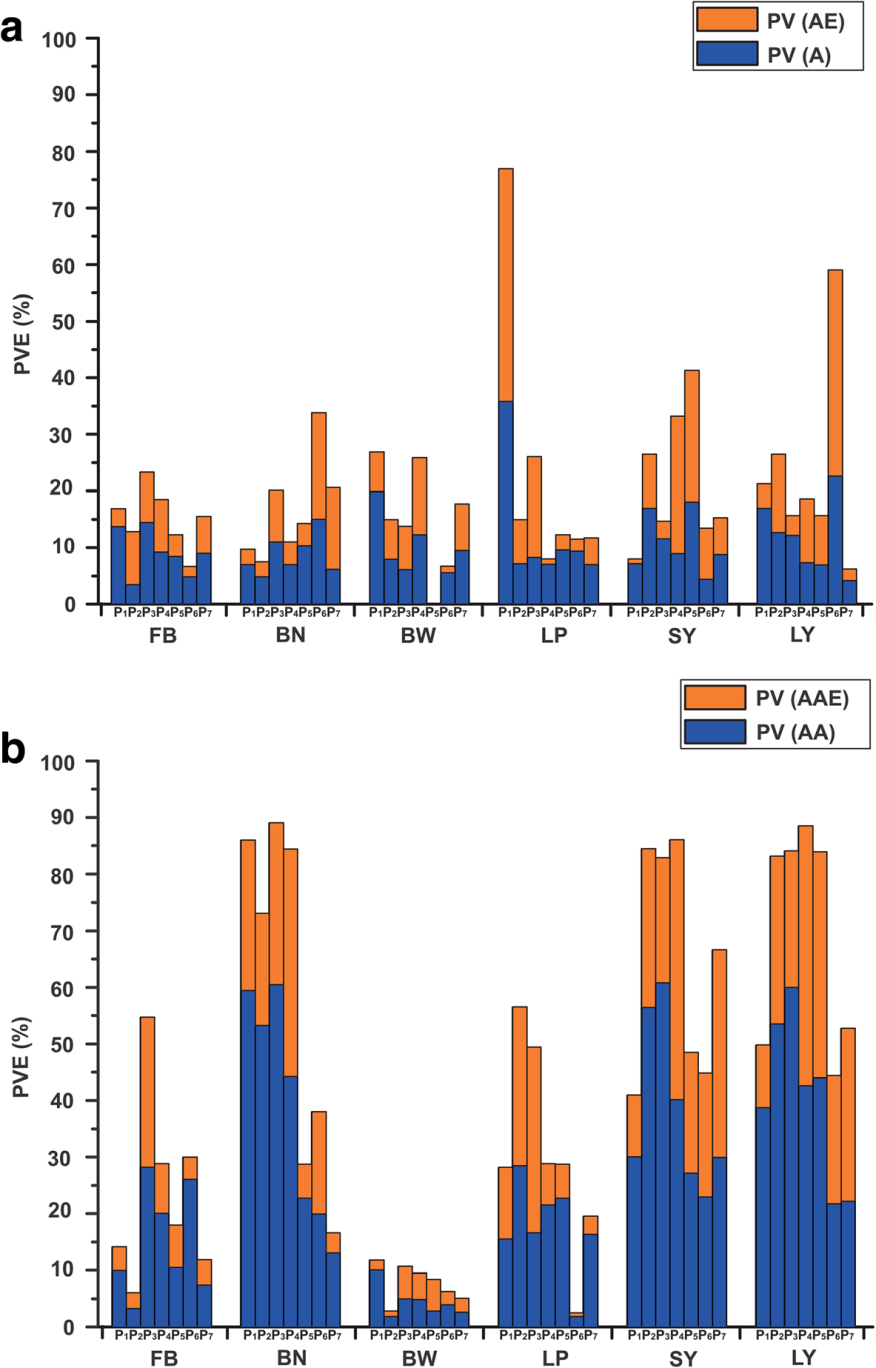
Phenotypic variance explained by the m-QTL and e-QTL effects for yield and yield components. **a** Phenotypic variance explained by the m-QTLs. PV: the phenotypic variance that the total effects explained; PV (A): the phenotypic variation that the main effect explained; PV (AE): the phenotypic variation that the environmental interaction effect explained. **b** Phenotypic variance explained by the e-QTLs. PV: the phenotypic variation that the total epistasis effect explained; PV (AA): the phenotypic variation that the main epistasis effect explained; PV (AAE): the phenotypic variation that the environmental interaction of the epistasis effect explained. P_1_: RILs; P_2_: IF_2_s; P_3_: HSBCF_1_s; P_4_: MARBCF_1_s; P_5_: IF_2_MPHs; P_6_: HSBCF_1_MPHs; P_7_: MARBCF_1_MPHs

The m-QTLs and QEs detected for yield and yield components in the RILs, IF_2_s, HSBCF_1_s, MARBCF_1_s, IF_2_MPHs, HSBCF_1_MPHs, and MARBCF_1_MPHs are shown in Fig. 2, Additional file 7: Table S6, and Additional file 8: Table S7.

A total of 48 m-QTLs and QEs were identified in the RIL population. On average, m-QTLs explained 2.37% of the PV, and the QEs explained 0.90% of the PV. Three major m-QTLs related to LP, RmaqLP-C07–1, RmaqLP-C08–1, and RmaqLP-C09–1, were found to account for more than 10% of the total explained PV (PV (A) and PV (AE)). For the IF_2_ population, 60 and 50 m-QTLs were identified in IF_2_ and IF_2_MPH datasets, respectively. On average, m-QTLs detected in the IF_2_ and IF_2_MPH datasets explained 0.92 and 1.36% of the PV (A), respectively, and the QEs explained 0.97 and 1.26% of the PV (AE), respectively. One locus, IMmaqLP-C10–1, was considered as a major m-QTL with 10.69% of the total PV explained. In the HSBCF_1_ population, a total of 24 and 21 m-QTLs were detected in HSBCF_1_ and HSBCF_1_MPH datasets, respectively. In HSBCF_1_s, the number of m-QTLs varied from two to five for different traits, with an average of 2.40% of the PV (A) and 2.53% of the PV (AE). Furthermore, in HSBCF_1_MPHs, the number of m-QTLs varied from zero to seven for different traits, with an average of 2.40% of PV (A) and 1.92% of PV (AE). No m-QTL was detected for BW. Two m-QTLs, B_1_MmaqSY-C14–1 and B_1_MmaqLY-C14–1, were found to have major effects, and were located in the same marker interval of i28957Gh-i36740Gh. In the MARBCF_1_ population, there were 28 m-QTLs in MARBCF_1_s and 15 in MARBCF_1_MPHs detected, on average, explaining 4.25 and 6.12% of the total PV in F_1_ performance and MPH, respectively. In MARBCF_1_s, B_2_maqBN-C03–1 and B_2_maqLY-C21–1 explained more than 10% of the total PV. In MARBCF_1_MPHs, B_2_MmaqBN-C21–1 was identified to be a major m-QTL, with 13.35% of the total PV explained.

### Epistatic QTLs in RIL, IF_2_, and two BCF_1_ populations

A total of 72, 124, 126, 73, 147, 73, and 67 e-QTLs pairs were identified in the RILs, IF_2_s, HSBCF_1_s, MARBCF_1_s, IF_2_MPHs, HSBCF_1_MPHs, and MARBCF_1_MPHs, respectively (Fig. 2, Table 5, Additional file 9: Table S8, Additional file 10: Table S9). These e-QTLs explained most of the variation for yield traits. For example, the e-QTLs of SY in IF_2_s and IF_2_MPHs explained more than 80% of the total PV. The e-QTLs in HSBCF_1_s and HSBCF_1_MPHs explained 91.89 and 51.68% of the total PV for SY, respectively, and those in MARBCF_1_s and MARBCF_1_MPHs explained 47.75 and 71.12% of the total PV, respectively. In addition, the environmental interactions of these e-QTLs also accounted for considerable PV. On average, the QEs of e-QTLs for each trait explained 12.00, 19.52, 27.27, 12.40, 24.96, 15.41, and 14.45% of the PV in RILs, IF_2_s, HSBCF_1_s, MARBCF_1_s, IF_2_MPHs, HSBCF_1_MPHs, and MARBCF_1_MPHs, respectively.

**Table 5:** Type of epistatic interactions and the total phenotypic variation explaining by e-QTLs

Population	Traits^a^	Type of epistasis^b^			Sum^c^	Total variation (%)^d^		
		I	II	III		PV^d^	PV(AA)^d^	PV(AAE)^d^
RIL	FB	0	0	4	4	14.92	10.40	4.51
	BN	0	0	27	27	91.79	63.38	28.40
	BW	0	0	4	4	12.42	10.50	1.92
	LP	0	3	7	10	29.95	16.35	13.60
	SY	0	0	12	12	43.68	31.96	11.72
	LY	0	0	15	15	53.08	41.23	11.85
IF_2_	FB	0	0	2	2	6.29	3.28	3.00
	BN	0	0	29	29	78.01	56.76	21.25
	BW	0	0	1	1	2.81	1.78	1.03
	LP	0	1	25	26	60.30	30.27	30.03
	SY	0	3	31	34	90.19	60.16	30.03
	LY	0	1	31	32	88.78	57.03	31.75
HSBCF_1_	FB	0	1	11	12	30.68	21.23	9.45
	BN	0	0	35	35	90.14	47.12	43.02
	BW	0	0	4	4	9.97	4.96	5.01
	LP	0	0	11	11	30.68	22.80	7.88
	SY	0	3	28	31	91.89	42.76	49.13
	LY	0	1	32	33	94.48	45.38	49.10
MARBCF_1_	FB	0	0	13	13	31.86	27.67	4.20
	BN	0	0	17	17	40.47	21.10	19.37
	BW	0	0	3	3	6.51	3.97	2.53
	LP	0	0	1	1	2.46	1.76	0.70
	SY	0	2	17	19	47.75	24.34	23.42
	LY	0	7	13	20	47.27	23.08	24.19
IF_2_MPH	FB	0	1	21	22	58.32	29.95	28.37
	BN	0	4	29	33	95.10	64.48	30.62
	BW	0	0	4	4	11.22	5.06	6.15
	LP	0	1	20	21	52.67	17.53	35.14
	SY	0	0	34	34	88.49	64.82	23.66
	LY	0	1	32	33	89.77	63.94	25.83
HSBCF_1_MPH	FB	0	0	7	7	19.02	10.99	8.03
	BN	0	1	10	11	30.54	24.09	6.45
	BW	0	0	4	4	8.76	2.83	5.93
	LP	0	1	10	11	30.54	24.09	6.45
	SY	0	1	22	23	51.68	28.82	22.86
	LY	0	2	40	42	89.61	46.85	42.76
MARBCF_1_MPH	FB	0	0	5	5	12.45	7.74	4.72
	BN	0	0	7	7	17.56	13.77	3.79
	BW	0	0	2	2	5.20	2.55	2.65
	LP	0	0	7	7	20.72	17.25	3.48
	SY	0	2	25	27	71.12	31.81	39.31
	LY	0	0	19	19	56.23	23.50	32.73

^a^*FB* fruit branches per plant, *BN* boll numbers per plant, *BW* boll weight, *LP* lint percentage, *SY* seed cotton yield, *LY* lint yield

^b^Type of epistasis: (I) two loci with m-QTL, (II) one loci with m-QTL and the other loci without significant m-QTL and (III) two loci without significant m-QTL

^c^Sum total number of epistatic interactions

^d^*PV* the phenotypic variation that the total epistasis effect explained, *PV(AA)* the phenotypic variation that the main epistasis effect explained, *PV (AAE)* the phenotypic variation that the environmental interaction of the epistasis effect explained

The e-QTLs were classified into three types: (I) two loci with m-QTL; (II) one loci with m-QTL and a locus without significant m-QTL; and (III) two loci without significant m-QTL [15]. For these e-QTLs in the RIL population, three pairs of LP QTLs were type II, and the remaining interactions were type III. No type I interactions were observed (Table 5). For these e-QTLs in the IF_2_ population, five pairs in IF_2_s and seven pairs in IF_2_MPHs were type II and the remaining interactions were type III. Of these e-QTLs in the HSBCF_1_ population, five pairs were detected between an interval with significant additive effect and other loci. The remaining interactions occurred between two complementary loci. Of these e-QTLs in the MARBCF_1_ population, nine pairs in MARBCF_1_s and two pairs in MPHs were type II, and all remaining interactions were type III.

### Congruence analysis of the single-locus QTLs and main-effect QTLs

Comparing the identified additive QTLs, the confidence intervals of 63 single-locus QTLs identified by the CIM method overlapped with 77 m-QTLs identified by the ICIM method, of which some single-locus QTLs harbored two or more m-QTLs identified in different datasets (Additional file 5: Figure S1, Additional file 6: Table S5, Additional file 7: Table S6, Additional file 8: Table S7).

For FB, four stable single-locus QTLs, qFB-C05–2, qFB-C06–2, qFB-C06–3, and qFB-C16–1, had the same or overlapping confidence intervals of four m-QTLs, ImaqFB-C05–1, IMmaqFB-C06–1, B_1_maqFB-C06–1 and B_1_MmaqFB-C16–1, respectively. The six m-QTLs RmaqFB-C02–1, RmaqFB-C07–1, B_1_maqFB-C13–1, IMmaqFB-C22–1, RmaqFB-C24–1, and IMmaqFB-C26–1 also had overlapping confidence intervals with the QTLs qFB-C02–1, qFB-C07–1, qFB-C13–1, qFB-C22–1, qFB-C24–1, and qFB-C26–1, respectively, which could only be detected in one environment.

For BN, the confidence interval of the stable single-locus QTL, qBN-C01–2, detected in the 2015Bg environment in IF_2_s and IF_2_MPHs, harbored two m-QTLs, ImaqBN-C01–1 and IMmaqBN-C01–1. The other two stable single-locus QTLs, qBN-C02–2 and qBN-C18–1, had the same or overlapping confidence intervals with two m-QTLs, B_1_maqBN-C02–1 and IMmaqBN-C18–1, respectively. The remaining five m-QTLs had overlapping confidence intervals with four single-locus QTLs that could only be detected in one environment. Among them, qBN-C14–3 harbored two m-QTLs.

For BW, two stable single-locus QTLs, qBW-C08–3 and qBW-C10–1, had the same or overlapping confidence intervals with two m-QTLs, B_1_maqBW-C08–1 and ImaqBW-C10–1, respectively. The confidence interval of the stable single-locus QTL qBW-C03–1 harbored two m-QTLs, IMmaqBW-C03–1 and B_2_MmaqBW-C03–2. Similarly, the single-locus QTL qBW-C01–2, which was detected in one environment in IF_2_s, also harbored two m-QTLs, ImaqBW-C01–2 and IMmaqBW-C01–1. The other six m-QTLs had one-to-one corresponding confidence intervals with six single-locus QTLs that could only be detected in one environment.

For LP, five stable single-locus QTLs, qLP-C10–1, qLP-C13–2, qLP-C13–3, qLP-C18–2, and qLP-C20–1, had the same or overlapping confidence intervals with five m-QTLs, B_2_maqLP-C10–1, RmaqLP-C13–2, B_1_maqLP-C13–1, IMmaqLP-C18–2, and ImaqLP-C20–1, respectively. The confidence interval of the stable single-locus QTL qLP-C04–2, detected in 2014Yc and 2014Bg in the RILs and 2015Bg in IF_2_s, harbored two m-QTLs, ImaqLP-C04–1 and RmaqLP-C04–1. The other four m-QTLs had one-to-one corresponding confidence intervals with four single-locus QTLs, although they could only be detected in one environment.

For SY, seven stable single-locus QTLs, qSY-C02–2, qSY-C05–1, qSY-C13–4, qSY-C13–5, qSY-C18–3, qSY-C23–1, and qSY-C24–2, had the same or overlapping confidence intervals with seven m-QTLs, ImaqSY-C02–1, ImaqSY-C05–1, B_1_MmaqSY-C13–2, IMmaqSY-C13–2, ImaqSY-C18–1, ImaqSY-C23–1, and ImaqSY-C24–1, respectively. The confidence interval of the stable single-locus QTL qSY-C05–2, which was simultaneously detected in HSBCF_1_s and HSBCF_1_MPHs, harbored two m-QTLs, B_1_maqSY-C05–1 and B_1_MmaqSY-C03–2. In addition, eight m-QTLs had overlapping confidence intervals with six single-locus QTLs that could only be detected in one environment. Among them, two single-locus QTLs harbored two m-QTLs, respectively.

For LY, the confidence intervals of three stable single-locus QTLs, qLY-C02–3, qLY-C24–1, and qLY-C26–2, overlapped with three m-QTLs, IMmaqLY-C02–1, ImaqLY-C24–3, and RmaqLY-C26–1, respectively. Stable QTLs qLY-C21–1 and qLY-C02–2 overlapped with three m-QTLs (B_2_MmaqLY-C21–1, ImaqLY-C21–1, and IMmaqLY-C21–1) and two m-QTLs (ImaqLY-C02–1 and B_1_maqLY-C02–1), respectively. In addition, eight m-QTLs overlapped with seven single-locus QTLs that could only be detected in one environment. Among them, one single-locus QTL harbored two m-QTLs.

## Discussion

### Application of RIL, IF_2_ and BCF_1_ populations

RIL and doubled haploid (DH) populations are permanent populations that can be repeated in different environments to detect valuable QTLs in multi-environments [47, 48]. BCF_1_ or IF_2_ populations based on RIL or DH populations have been constructed previously to conduct QTL mapping with respect to heterosis [15, 25, 27, 43, 49]. However, no one has used different segregating populations from the same parental combination to study heterosis. Our experimental schemes using related RIL, IF_2_ and two BCF_1_ populations were specifically designed to allow simultaneous and comprehensive mapping of loci contributing yield and yield components heterosis in upland cotton. Based on this, more heterozygous loci were uncovered, and more QTLs were detected than from a single population. Some QTLs that could not be identified in RIL population could be detected in IF_2_/BCF_1_ populations, and the QTLs detected in RILs could be confirmed using the IF_2_/BCF_1_ populations. Furthermore, through the combination of these four populations, both additive and non-additive gene actions of the detected loci were more accurately identified. For instance, the QTL main effects obtained using the F_1_ mean values of the IF_2_/BCF_1_ populations contained both additive and dominance effects while those obtained from the MPH values were estimates of the dominant effect [50]. Similarly, for the epistatic loci, the estimated epistatic effects using the mean F_1_ values contained both additive and nonadditive epistatic interactions of the epistatic QTL, while those from MPH values represent the DD interactions [50].

### Detection of heterotic loci

Detection of HL using the MPH measurements enabled by the IF_2_ and two BCF_1_ populations represents another feature of the study. This analysis method effectively separated the single-locus effects causing heterosis from the QTL concerning the trait performance as detected in most previous QTL studies. Making use of the MPH data, we detected 47, 65 and 45 HLs for yield and its components in the IF_2_, HSBCF_1_, and MARBCF_1_ populations, respectively. Moreover, 16 stable HLs were detected in two environments, of which five showed inconsistent parental sources of favorable alleles in different environments, which indicated a high sensitivity of the HLs to the environment. To some extent, this should be taken into account in upland cotton breeding. The remaining 11 stable HLs, including three for BN, five for SY, and two for LY could be important for the application of MAS in upland cotton breeding in the future. Here, the BN trait harbored more stable HLs than other yield components, which may be attributed to its higher average heterosis of the 20 top high-heterosis hybrids (Additional file 3: Table S3). Hua et al. [25] found that only ten of 33 HLs identified in their analysis were detected by QTL analysis using trait performance and indicated that trait performance and heterosis were controlled by different sets of loci. However, HLs detected in our study were not independent, and a subset overlapped with QTLs controlling trait phenotypes. Among all HLs, 12, 30, and 20 HLs identified in IF_2_MPH, HSBCF_1_MPH, and MARBCF_1_MPH datasets were also detected by QTL analysis using the data of the IF_2_s, HSBCF_1_s, and MARBCF_1_s, respectively. This result suggests that the MPH and performance per se of the hybrid might share identical genetic modes of action in upland cotton. In fact, it is impossible to demonstrate the genetic mechanism underlying yield traits without involving heterosis.

### Genetic bases of inbreeding depression and heterosis of yield and yield components

In the present study, the levels of hybrid breakdown were ordered as LY > SY > BN > LP > FB > > BW, while heterosis was LY > SY > BW > FB > LP > > BN. All traits showed moderate and low heritability except for LP. This tendency toward more complex yield traits showing much greater levels of inbreeding depression and heterosis has been universally observed in many crops [17, 23]. The traits with serious inbreeding depression did not necessarily possess high heterosis in hybrids, which implied that there are differences in the mechanisms controlling these two biological phenomena. At the same time, wide variations were also observed in mid-parental heterosis of the IF_2_ and two BCF_1_ populations. The performances of some hybrids were better than those of the MP values of the original parents, while some other hybrids showed the opposite. Similar results were also obtained by Luo et al. [20, 43]. Together, it can be speculated that high heterosis is derived from heterozygosity at certain loci but not from genome-wide heterozygosity [17, 24, 43, 49, 51, 52].

The reduction of the RIL population from the midparental value was highly significant. This might be attributed to the homozygosity of deleterious alleles and/or less fit multilocus genotypes during the development of RILs [15, 39, 40, 53]. Theoretically, the inbreeding depression values of individual RILs and the MPH values of IF_2_/BCF_1_ hybrids for yield and its components have three components. The first is additive gene action, which leads to the deviation of the RILs from the midparental value and hybrid breakdown [1, 39, 40]. The genes of this group are directly detected in the RILs but are confounded in the IF_2_s and BCF_1_s. The second is dominant gene action, which leads to the deviation of the F_1_ hybrids from their corresponding midparental value. Those genes are segregating and contribute to heterosis in the IF_2_s/BCF_1_s but are not directly detected in the RILs. The third is nonadditive gene action, which causes disharmonious interactions in inbreeding depression of RILs and beneficial interactions in heterosis of IF_2_s and BCF_1_s. In fact, there are some overlapping genes between inbreeding depression and heterosis. The overlapping genes of this type are particularly important since they contribute negatively to the mean value of the inbred RILs when homozygous (resulting in hybrid breakdown) and positively to heterosis when heterozygous.

In the IF_2_ population of our present study, 25 (26.60%) PD QTLs and 67 (71.28%) OD QTLs were identified. In the HSBCF_1_ population, 34 (34.34%) A QTLs, two (2.02%) PD QTLs, and 63 (63.64%) OD QTLs were detected. In the MARBCF_1_ population, 47 (51.09%) A QTLs, six (6.52%) PD QTLs, and 39 (42.39%) OD QTLs were detected (Table 4). These results revealed that the genetic basis of heterosis was varied in different populations. At the single-locus level, partial dominance and over-dominance were the main causes of heterosis in the IF_2_ population, and additive and over-dominance were the main genetic bases of heterosis in the two BCF_1_ populations. Similar results have been discovered in previous studies [28, 33, 43]. In addition, similar to Zhou et al. [26], our results showed that the relative contributions of the various genetic components to heterosis were trait specificity. Over-dominance and additive effects were the main contributors to heterosis for SY, LY, BW, and LP. Over-dominance, partial dominance, and additive effects all had roles in heterosis of BN. Over-dominance was the most important contributor to heterosis of FB. Overall, over-dominance played an important role in the formation of heterosis in these traits.

Epistasis is a common feature of most loci associated with inbreeding depression and heterosis. First, the e-QTLs explained a much greater portion of the total PV than the m-QTLs for the yield in each of the mapping populations, but this was not true for the yield components (Fig. 2, Table 5). This was consistent with the results of Li et al. [39], which indicated that complex traits tended to be determined by a greater degree of epistasis. In a similar experimental design, Xiao et al. [9] detected a single main-effect QTL which had an R^2^ of 6–7% for grain yield in each of the two rice BCF_1_ populations. However, the majority of the phenotypic variation was unexplained. Apparently, their failure to detect epistasis was largely attributed to the unavailability of an appropriate analytical method. With a similar experimental design, Shang et al. [23] reported m-QTLs and e-QTLs that had different proportions for yield and yield components in an upland cotton BCF_1_ population, but the relative importance of m-QTLs and e-QTLs was not evaluated. Second, most epistasis occurred between complementary loci with no detectable main effects (Table 5). Fewer cases of epistasis occur between m-QTLs and complementary loci. The predominance of epistasis between complementary loci indicate that yield and its component traits related e-QTLs occurred more in multilocus genotypes than in specific alleles at individual loci, which has been demonstrated by a large number of empirical studies [17, 23, 43]. In addition, the environment was a critical factor in the expression of these m-QTLs and e-QTLs. The average PV of m-QTLs and e-QTLs explained by QEs occupied a large proportion of the total PV in all seven datasets.

### Implications for MAS in yield improvement of upland cotton

Numerous classical genetic studies have clearly found that the phenotypic relationships between yield and its components in crops are complex, and the genetic bases of heterosis in segregating populations remains poorly understood. The results of our study have several implications. For breaking the yield ceiling of hybrid upland cotton cultivars, simultaneous selection for all yield components, with an emphasis on increased BN and BW, should be much more efficient than selecting for only lint yield. This is because both BN and BW were significantly positively correlation with LY, and the heterosis of BW in three hybrid populations was obvious and showed the same trend with SY and LY.

Although several QTLs for cotton yield traits have been detected previously using an intraspecific map, few shared markers were used in the present research. Furthermore, it is difficult to compare our results with previous yield QTLs due to the use of different maps, population types, population structure, and environments, etc. [33]. In our study, 12 stable QTLs were identified across at least four datasets or environments. Additionally, there were six stable QTLs, qBN-C05–1, qBN-C05–2, qBN-C22–3, qSY-C16–2, qSY-C19–1, and qSY-C24–2, that are also stable HLs. These stable QTLs across multiple populations and environments should greatly promote further interest in the fine mapping of yield traits or implementation of MAS. When we compared the single-locus QTLs from CIM with m-QTLs from ICIM, 29 stable single-locus QTLs overlapped with m-QTLs. qSY-C24–2 could be an important QTL identified in this study, as it was not only identified as a stable QTL and HL across multiple environments but was also simultaneously confirmed by both CIM and ICIM. The large dominance effects of this QTL justify its potential use in genetic improvement of the yield of both inbred and hybrid cultivars through marker-assistant transfer in upland cotton breeding programs.

## Conclusion

These results showed that obvious inbreeding depression was found in the RIL population and high levels of heterosis were detected in the IF_2_ and BCF_1_ populations for yield and its components in upland cotton. Heterosis of yield and its components definitely included the relative contributions of additive effects, partial dominance, over-dominance, and epistatic effects of multiple QTLs, which differed among populations and traits. Through integrating the results from single-locus and multi-environment QTL analysis, over-dominance and epistasis were found to be more important than the others. Furthermore, the heterosis genes can be further exploited because of the detection of significant HLs, which will greatly accelerate the hybrid breeding process of upland cotton.

## Additional files

Additional file 1: **Table S1.** Phenotypic variation of yield and yield components for the parents (PDF 105 kb)

Additional file 2: **Table S2.** HB of RILs and MPH percentage of yield and yield components across four environments. (PDF 100 kb)

Additional file 3: **Table S3.** Average MPH of 20 top high-heterosis hybrids of yield and yield components. (PDF 100 kb)

Additional file 4: **Table S4.** Correlations between yield and yield components estimated in the RIL, IF_2_, and two BCF_1_ populations. (PDF 61 kb)

Additional file 5: **Figure S1.** Chromosomal location of QTLs for yield and yield components in RIL, IF_2_, HSBCF_1_, MARBCF_1_, IF_2_MPH, HSBCF_1_MPH, and MARBCF_1_MPH datasets across four environments. Map distances are given in centimorgans (cM). Solid bars with different colors represent different traits, and the legend is given at the end of figure. FB: fruit branches per plant; BN: boll numbers per plant; BW: boll weight; LP: lint percentage; SY: seed cotton yield; LY: lint yield. (PDF 686 kb)

Additional file 6: **Table S5.** QTLs identified for yield and yield components in RILs, IF_2_s, HSBCF_1_s, MARBCF_1_s and their MPH datasets by using the CIM method. (PDF 668 kb)

Additional file 7: **Table S6.** Main effects and environmental interactions detected for yield and yield components in RIL, IF_2_ and two BCF_1_ datasets using the ICIM method. (PDF 404 kb)

Additional file 8: **Table S7.** Main effects and environmental interactions detected for yield and yield components in IF_2_MPH, HSBCF_1_MPH, and MARBCF_1_MPH datasets using the ICIM method. (PDF 261 kb)

Additional file 9: **Table S8.** Epistatic effects and environmental interactions detected for yield and yield components in RIL, IF_2_ and two BCF_1_ datasets using the ICIM method. (PDF 648 kb)

Additional file 10: **Table S9.** Epistatic effects and environmental interactions detected for yield and yield components in IF_2_MPH, HSBCF_1_MPH, and MARBCF_1_MPH datasets using the ICIM method. (PDF 631 kb)
