# Supplementary material for: Genetic basis of heterosis for yield and yield components explored by QTL mapping across four genetic populations in upland cotton
Source: BMC Genomics. 2018 Dec 12;19:910. doi: 10.1186/s12864-018-5289-2 (PMC6292039; doi:10.1186/s12864-018-5289-2)
Supplement: Supplementary file 1 — Table S1. Phenotypic variation of yield and yield components for the parents (PDF 105 kb) [file 12864_2018_5289_MOESM1_ESM.pdf]

Table S1 Phenotypic variation of yield and yield components for the parents

Traits [a]	Env. [b]	Parents [c]				
		P_1	P_2	MP	P_1-P_2	*P*-value
FB	2014Yc	8.93	7.62	8.28	1.31	0.0622
	2014Bg	8.71	7.80	8.25	0.91	
	2015Yc	9.53	9.50	9.51	0.03	
	2015Bg	9.41	9.34	9.38	0.07	
BN	2014Yc	17.22	12.82	15.02	4.40	0.0051
	2014Bg	15.21	13.02	14.11	2.19	
	2015Yc	11.64	11.37	11.51	0.27	
	2015Bg	11.57	11.47	11.52	0.10	
BW	2014Yc	5.43	4.91	5.17	0.51	0.0016
	2014Bg	5.81	5.37	5.59	0.44	
	2015Yc	5.70	5.38	5.54	0.32	
	2015Bg	5.93	5.53	5.73	0.40	
LP	2014Yc	37.07	38.74	37.90	-1.67	0.0057
	2014Bg	39.07	39.87	39.47	-0.81	
	2015Yc	36.80	37.48	37.14	-0.68	
	2015Bg	35.22	37.51	36.37	-2.29	
SY	2014Yc	653.07	500.98	577.03	152.09	0.0006
	2014Bg	688.61	524.50	606.55	164.10	
	2015Yc	689.56	637.48	663.52	52.08	
	2015Bg	710.93	665.00	687.97	45.93	
LY	2014Yc	242.84	194.54	218.69	48.29	0.0102
	2014Bg	268.71	208.84	238.77	59.87	
	2015Yc	243.07	237.53	240.30	5.54	
	2015Bg	250.27	248.44	249.35	1.83	

[a] FB: number of fruit branches per plant; BN: number of bolls per plant; BW: boll weight; LP: lint percentage; SY: seed cotton yield; LY: lint yield

[b] 2014Yc: Yacheng, Hainan Province in 2014; 2014Bg: Baogang, Hainan Province in 2014; 2015Yc: Yacheng, Hainan Province in 2015; 2015Bg: Baogang, Hainan Province in 2015

[c] P_1: HS46; P_2: MARCABUCAG8US-1-88
