# Supplementary material for: Genetic basis of heterosis for yield and yield components explored by QTL mapping across four genetic populations in upland cotton
Source: BMC Genomics. 2018 Dec 12;19:910. doi: 10.1186/s12864-018-5289-2 (PMC6292039; doi:10.1186/s12864-018-5289-2)
Supplement: Supplementary file 2 — Table S2. HB of RILs and MPH percentage of yield and yield components across four environments. (PDF 100 kb) [file 12864_2018_5289_MOESM2_ESM.pdf]

Table S2 HB of RILs and MPH percentage of yield and yield components across four environments

Traits[a]	RILHBs (%)			IF_2MPHs (%)			HSBCF_1MPHs (%)			MARBCF_1MPHs (%)		
	Mean	Min	Max	Mean	Min	Max	Mean	Min	Max	Mean	Min	Max
FB	-2.36	-38.80	36.22	4.35	-58.69	65.19	11.38	-40.74	82.42	11.41	-54.72	92.91
BN	-3.83	-43.85	75.28	-2.7019	-75.94	195.35	7.7178	-54.26	118.18	11.1245	-51.65	107.87
BW	2.75	-31.74	37.04	10.58	-39.57	60.79	9.34	-34.60	53.51	14.92	-32.55	84.25
LP	-3.32	-31.55	18.32	5.07	-22.61	70.98	2.05	-18.30	30.21	0.75	-24.67	25.57
SY	-7.09	-61.46	89.70	24.75	-61.43	202.74	15.42	-52.10	166.60	28.61	-49.74	244.41
LY	-7.30	-64.22	84.83	25.16	-61.63	204.12	16.66	-52.48	177.90	28.39	-49.29	248.76

[a] FB: number of fruit branches per plant; BN: number of bolls per plant; BW: boll weight; LP: lint percentage; SY: seed cotton yield; LY: lint yield
