# Supplementary material for: Genetic basis of heterosis for yield and yield components explored by QTL mapping across four genetic populations in upland cotton
Source: BMC Genomics. 2018 Dec 12;19:910. doi: 10.1186/s12864-018-5289-2 (PMC6292039; doi:10.1186/s12864-018-5289-2)
Supplement: Supplementary file 3 — Table S3. Average MPH of 20 top high-heterosis hybrids of yield and yield components. (PDF 100 kb) [file 12864_2018_5289_MOESM3_ESM.pdf]

Table S3 Average MPH of 20 top high-heterosis hybrids of yield and yield components

Traits [a]	Env. [b]	Average MPH of 20 top high-heterosis hybrids (%)		
		IF_2	$HSBCF_1$	$MARBCF_1$
FB	2014Yc	33.44	32.74	41.89
	2014Bg	31.75	28.69	31.23
	2015Yc	40.32	50.99	48.41
	2015Bg	40.33	52.11	47.76
BN	2014Yc	53.94	39.11	55.89
	2014Bg	45.71	43.17	45.95
	2015Yc	61.03	40.6	49.36
	2015Bg	62.46	49.24	46.44
BW	2014Yc	42.56	42.98	50.00
	2014Bg	40.09	27.11	43.96
	2015Yc	29.03	28.87	30.08
	2015Bg	31.23	23.13	31.17
LP	2014Yc	11.98	11.84	9.45
	2014Bg	15.17	8.79	10.14
	2015Yc	42.66	19.11	17.23
	2015Bg	15.67	11.01	7.21
SY	2014Yc	152.28	83.36	133.21
	2014Bg	128.19	87.7	116.32
	2015Yc	63.30	46.51	49.94
	2015Bg	65.23	48.99	47.96
LY	2014Yc	145.56	88.34	132.09
	2014Bg	142.92	89.57	119.09
	2015Yc	62.00	48.46	49.28
	2015Bg	69.69	51.47	46.13

[a] PB: number of fruit branches per plant; NB: number of bolls per plant; BW: boll weight; LP: lint percentage; SY: seed cotton yield; LY: lint yield

[b] 2014Yc: Yacheng, Hainan Province in 2014; 2014Bg: Baogang, Hainan Province in 2014; 2015Yc: Yacheng, Hainan Province in 2015; 2015Bg: Baogang, Hainan Province in 2015
