# Supplementary material for: Genetic basis of heterosis for yield and yield components explored by QTL mapping across four genetic populations in upland cotton
Source: BMC Genomics. 2018 Dec 12;19:910. doi: 10.1186/s12864-018-5289-2 (PMC6292039; doi:10.1186/s12864-018-5289-2)
Supplement: Supplementary file 4 — Table S4. Correlations between yield and yield components estimated in the RIL, IF2, and two BCF1 populations. (PDF 61 kb) [file 12864_2018_5289_MOESM4_ESM.pdf]

Table S4 Correlations between yield and yield components estimated in the RIL, IF_2, and two BCF_1 populations

Traits[a]	Population	FB	BN	BW	LP	SY	LY
BN	RIL	0.262**					
	IF_2	0.290**					
	$HSBCF_1$	0.082*					
	$MARBCF_1$	0.135**					
BW	RIL	0.144**	-0.126**				
	IF_2	-0.069*	0.113**				
	$HSBCF_1$	-0.233**	0.203**				
	$MARBCF_1$	-0.249**	0.155**				
LP	RIL	-0.249**	0.246**	-0.257**			
	IF_2	0.026	0.058	0.796**			
	$HSBCF_1$	-0.387**	0.157**	0.129**			
	$MARBCF_1$	-0.411**	0.094*	0.167**			
SY	RIL	0.388**	0.209**	0.485**	-0.227**		
	IF_2	0.084**	0.583**	0.174**	-0.052		
	$HSBCF_1$	0.178**	0.506**	0.319**	0.113**		
	$MARBCF_1$	0.025	0.585**	0.397**	0.135**		
LY	RIL	0.344**	0.253**	0.458**	-0.071	0.967**	
	IF_2	0.023	0.551**	0.149**	-0.059*	0.967**	
	$HSBCF_1$	0.078	0.517**	0.332**	0.343**	0.971**	
	$MARBCF_1$	-0.064	0.573**	0.413**	0.345**	0.975**	

*, ** indicate that the correlation is significant at 0.05 and 0.01 probability levels, respectively

[a] FB: number of fruit branches per plant; BN: number of bolls per plant; BW: boll weight; LP: lint percentage; SY: seed cotton yield; LY: lint yield
