# Supplementary figures and images for: Genetic basis of heterosis for yield and yield components explored by QTL mapping across four genetic populations in upland cotton
Source: BMC Genomics. 2018 Dec 12;19:910. doi: 10.1186/s12864-018-5289-2 (PMC6292039; doi:10.1186/s12864-018-5289-2)

**Chr01**

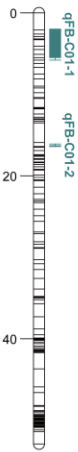

**Chr02**

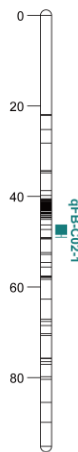

**Chr03**

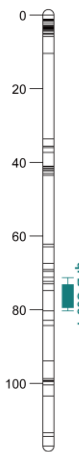

**Chr04**

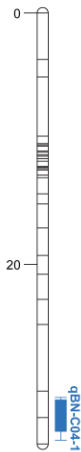

**Chr05**

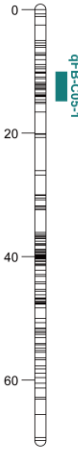

**Chr06**

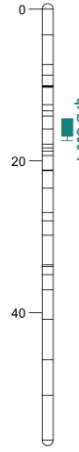

**Chr07**

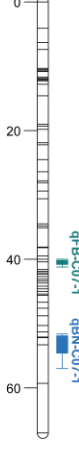

**Chr08**

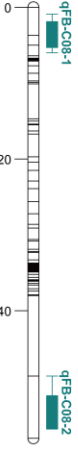

**Chr09**

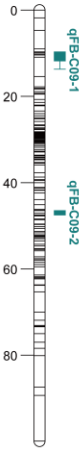

**Chr10**

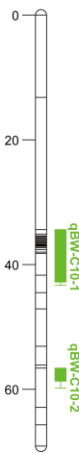

**Chr11**

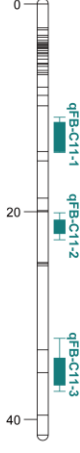

**Chr12**

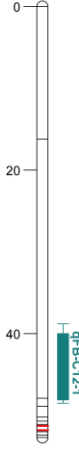



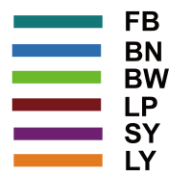

Chr26

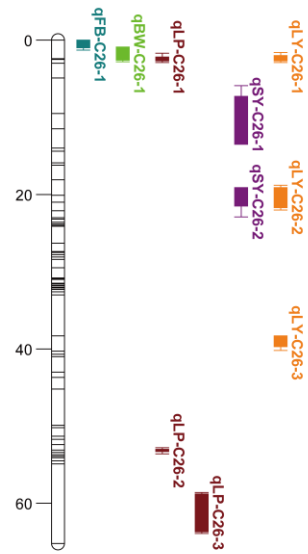

Chr25

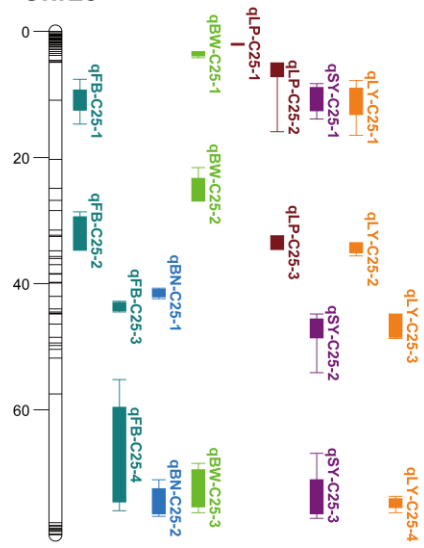

Supplement: Supplementary file 5 — Figure S1. Chromosomal location of QTLs for yield and yield components in RIL, IF2, HSBCF1, MARBCF1, IF2MPH, HSBCF1MPH, and MARBCF1MPH datasets across four environments. Map distances are given in centimorgans (cM). Solid bars with different colors represent different traits, and the legend is given at the end of figure. FB: fruit branches per plant; BN: boll numbers per plant; BW: boll weight; LP: lint percentage; SY: seed cotton yield; LY: lint yield. (PDF 686 kb) [file 12864_2018_5289_MOESM5_ESM.pdf]
