# Supplementary material for: Genetic basis of heterosis for yield and yield components explored by QTL mapping across four genetic populations in upland cotton
Source: BMC Genomics. 2018 Dec 12;19:910. doi: 10.1186/s12864-018-5289-2 (PMC6292039; doi:10.1186/s12864-018-5289-2)
Supplement: Supplementary file 6 — Table S5. QTLs identified for yield and yield components in RILs, IF2s, HSBCF1s, MARBCF1s and their MPH datasets by using the CIM method. (PDF 668 kb) [file 12864_2018_5289_MOESM6_ESM.pdf]

Table S5 QTLs identified for yield and yield components in RILs, IF_2, $HSBCF_1$, $MARBCF_1$ populations and their MPH datasets using the CIM method

Trait[a]	QTL	Environment[b]	Flanking markers	Position[c]	LOD[d]	A[e]	D[e]	A+D[e]	R^2（%）[f]	Population
FB	qFB-C01-1	2015Bg	i33646Gh and i34473Gh	2.11	5.14	0.37			9.69	RIL
		2015Yc	i31090Gh and i60883Gt	4.21	3.21	0.23			5.73	RIL
	qFB-C01-2	2015Yc	i27043Gh and i45431Gh	16.41	3.57		-1.00		8.37	IF_2MPH
	qFB-C02-1	2014Yc	i46092Gh and i39133Gh	47.31	3.19		1.37		23.42	IF_2MPH
	qFB-C02-2	2014Yc	i21849Gh and i28841Gh	58.41	3.53		-0.66		10.62	IF_2MPH
	qFB-C02-3	2014Bg	i14841Gh and i30800Gh	81.51	3.35		-0.26		9.08	$MARBCF_1$MPH
	qFB-C03-1	2014Yc	i30069Gh and i45963Gh	74.81	4.20			-0.79	5.37	$MARBCF_1$
	qFB-C05-1	2014Yc	i36865Gh and i45777Gh	10.31	3.04	0.35	0.12		7.93	IF_2
		2014Bg	i17756Gh and i21516Gh	11.21	3.18	-2.23	1.08		30.04	IF_2
		2015Yc	i20957Gh and i00186Gh	14.61	3.48		0.56		12.15	IF_2MPH
	qFB-C05-2	2014Yc	i35761Gh and i09052Gh	17.61	3.32	0.45	0.47		6.99	IF_2
		2015Yc	i09497Gh and i09052Gh	17.61	3.18	-0.45	0.00		11.32	IF_2
		2014Yc	i35761Gh and i37479Gh	20.31	3.13	-0.37			7.35	RIL
	qFB-C06-1	2015Yc	i19214Gh and i43320Gh	15.11	3.76		-0.06		13.05	$MARBCF_1$MPH
		2014Yc	i19214Gh and i43320Gh	15.81	3.19		0.25		8.97	IF_2MPH
		2015Yc	i19214Gh and i43320Gh	15.81	4.60			0.23	11.91	$MARBCF_1$
	qFB-C06-2	2014Bg	i21566Gh and i06526Gh	23.61	3.18		0.62		9.51	IF_2MPH
		2015Yc	i21566Gh and i06526Gh	24.61	6.08			-0.70	19.00	$MARBCF_1$
		2015Yc	i21566Gh and i06526Gh	24.61	5.60		0.25		22.20	$MARBCF_1$MPH
	qFB-C06-3	2015Yc	i14061Gh and i15830Gh	30.81	3.92			-0.83	10.07	$MARBCF_1$
		2015Bg	i34827Gh and i06036Gh	31.81	4.73			0.54	31.29	$HSBCF_1$
		2015Yc	i15830Gh and i06037Gh	34.01	5.91		-1.17		31.66	$HSBCF_1$MPH
	qFB-C07-1	2014Bg	i34772Gh and i22256Gh	40.41	4.00		0.86		10.58	IF_2MPH

qFB-C08-1	2015Bg	i63682Gm and i40270Gh	3.71	3.35		0.61		7.04	HSBCF_1MPH
qFB-C08-2	2015Yc	i01126Gh and i04719Gh	53.61	3.88	0.76	0.81		4.52	IF_2
qFB-C09-1	2015Bg	i44872Gh and i07864Gh	9.91	3.77		-1.44		5.80	MARBCF_1MPH
qFB-C09-2	2014Bg	i33836Gh and i05402Gh	47.11	3.03		-0.62		7.63	IF_2MPH
qFB-C11-1	2015Yc	i47563Gh and i07468Gh	12.81	3.78		1.59		4.11	IF_2MPH
qFB-C11-2	2015Yc	i44818Gh and i07163Gh	21.01	4.78			-0.86	7.89	MARBCF_1
qFB-C11-3	2015Bg	i40251Gh and i43181Gh	35.51	3.53		0.13		7.81	IF_2MPH
qFB-C12-1	2014Yc	i48211Gh and i08275Gh	45.21	3.35		4.13		21.94	HSBCF_1MPH
qFB-C13-1	2014Bg	i20297Gh and i00187Gh	39.21	5.80		-1.39		14.31	HSBCF_1MPH
qFB-C14-1	2014Yc	i15536Gh and i05487Gh	1.11	4.88		4.97		17.67	HSBCF_1MPH
qFB-C14-2	2015Bg	i34963Gh and i44045Gh	50.51	4.02			-1.80	4.68	HSBCF_1
qFB-C15-1	2014Yc	i02538Gh and i35620Gh	17.71	3.10			0.07	8.04	MARBCF_1
qFB-C16-1	2015Bg	i29663Gh and i36412Gh	33.41	4.92		0.26		13.81	HSBCF_1MPH
	2015Bg	i29663Gh and i00384Gh	34.01	3.40			-0.43	7.12	HSBCF_1
qFB-C16-2	2014Bg	i27803Gh and i54704Gb	61.31	4.05			-0.84	3.65	MARBCF_1
qFB-C18-1	2014Bg	i13754Gh and i20346Gh	15.21	3.55	-0.29			7.40	RIL
qFB-C18-2	2015Yc	i32883Gh and i13851Gh	32.11	3.63	-0.30	1.06		29.65	IF_2
qFB-C18-3	2014Yc	i18042Gh and i18044Gh	57.21	3.27		3.59		3.15	IF_2MPH
qFB-C19-1	2014Bg	i09110Gh and i16566Gh	29.31	3.13		-1.24		17.06	MARBCF_1MPH
qFB-C21-1	2015Bg	i06952Gh and i07714Gh	12.91	3.57		0.13		21.94	IF_2MPH
qFB-C22-1	2015Yc	i20228Gh and i12581Gh	24.01	3.13		0.41		5.92	HSBCF_1MPH
qFB-C22-2	2015Yc	i12479Gh and i44682Gh	43.31	3.24		1.47		5.88	IF_2MPH
	2015Yc	i12464Gh and i44682Gh	43.31	4.96		0.43		22.24	HSBCF_1MPH
qFB-C23-1	2014Bg	i06287Gh and i06171Gh	16.01	4.23	-1.01	2.21		12.79	IF_2
qFB-C24-1	2015Bg	i04776Gh and i35290Gh	52.41	3.83			-0.08	9.75	HSBCF_1
qFB-C25-1	2014Bg	i00735Gh and i42629Gh	10.91	3.67		-0.71		4.09	HSBCF_1MPH

	qFB-C25-2	2014Yc	i11464Gh and i46788Gh	30.41	3.20	0.44	0.20		9.00	IF_2
		2014Yc	i18176Gh and i11465Gh	33.51	3.41		-1.38		11.02	IF_2MPH
	qFB-C25-3	2014Yc	i55440Gb and i11267Gh	44.01	4.97			-0.51	13.52	HSBCF_1
		2014Yc	i55440Gb and i30347Gh	44.01	3.39		-0.81		8.24	HSBCF_1MPH
	qFB-C25-4	2015Bg	i17145Gh and i10628Gh	70.51	4.08			0.74	27.09	MARBCF_1
	qFB-C26-1	2014Bg	i00879Gh and i08691Gh	0.01	3.47		-0.12		10.66	IF_2MPH
BN	qBN-C01-1	2015Yc	i48104Gh and i21823Gh	8.11	5.60		-0.71		9.92	IF_2MPH
	qBN-C01-2	2015Bg	i53010Gb and i41337Gh	10.61	3.10	-0.68	-0.22		6.44	IF_2
		2015Bg	i53010Gb and i41337Gh	10.61	5.93		-0.42		9.66	IF_2MPH
	qBN-C01-3	2015Yc	i02201Gh and i45431Gh	16.41	3.00		-1.14		8.95	IF_2MPH
	qBN-C01-4	2014Bg	i32268Gh and i63224Gt	28.11	4.24			-1.71	14.20	HSBCF_1
	qBN-C01-5	2014Yc	i40501Gh and i26341Gh	49.91	3.77	-3.99	-0.09		7.73	IF_2
	qBN-C02-1	2015Bg	i02761Gh and i16954Gh	25.11	3.76	2.71	-2.42		20.34	IF_2
		2014Yc	i02712Gh and i20804Gh	27.31	3.75	-3.77	-0.02		10.43	IF_2
	qBN-C02-2	2014Yc	i02258Gh and i02758Gh	44.51	6.24			1.36	6.61	HSBCF_1
		2015Bg	i02258Gh and i05624Gh	44.51	4.85			3.46	25.64	MARBCF_1
		2014Yc	i05624Gh and i39133Gh	45.11	3.29	-1.39	3.48		9.08	IF_2
	qBN-C02-3	2014Bg	i38985Gh and i30800Gh	82.51	4.33		-0.48		17.67	MARBCF_1MPH
	qBN-C04-1	2015Bg	i36496Gh and i10499Gh	31.11	4.55		-1.28		30.75	IF_2MPH
	qBN-C05-1	2015Yc	i23000Gh and i46433Gh	12.61	3.25			-0.66	26.74	MARBCF_1
		2015Bg	i23000Gh and i46433Gh	12.61	3.85			-0.31	34.41	MARBCF_1
		2015Yc	i23000Gh and i46433Gh	12.61	4.13		-3.78		33.59	MARBCF_1MPH
		2014Yc	i09498Gh and i20957Gh	14.11	3.22		-7.48		27.49	MARBCF_1MPH
	qBN-C05-2	2014Yc	i47720Gh and i53001Gb	25.81	5.46			-0.17	22.35	HSBCF_1
		2014Yc	i47720Gh and i53001Gb	25.81	3.77		-6.14		19.93	HSBCF_1MPH
		2014Bg	i47720Gh and i53001Gb	25.81	3.63		-9.75		26.29	HSBCF_1MPH

	2015Yc	i47720Gh and i53001Gb	26.11	5.87			0.52	8.11	HSBCF$_1$
qBN-C05-3	2015Bg	i16543Gh and i22374Gh	33.41	5.13			0.32	32.45	MARBCF$_1$
qBN-C06-1	2015Bg	i19214Gh and i31843Gh	15.11	4.27			-3.96	5.24	MARBCF$_1$
	2015Yc	i19214Gh and i43320Gh	15.81	4.33			0.25	11.28	MARBCF$_1$
qBN-C07-1	2014Yc	i01703Gh and i14398Gh	52.11	3.26			-0.80	27.62	HSBCF$_1$
	2014Yc	i01703Gh and i50051Gb	52.11	5.13		-6.57		32.83	HSBCF$_1$MPH
qBN-C08-1	2015Bg	i30195Gh and i04557Gh	35.61	8.32			4.32	6.78	MARBCF$_1$
qBN-C09-1	2014Bg	i07864Gh and i50078Gb	17.41	4.09			-0.22	13.39	HSBCF$_1$
	2015Bg	i07864Gh and i50078Gb	17.81	4.18			-0.23	9.01	MARBCF$_1$
qBN-C09-2	2014Yc	i26826Gh and i32479Gh	46.51	3.07	4.34			14.29	RIL
qBN-C11-1	2014Yc	i33855Gh and i43823Gh	5.31	4.14		10.53		18.14	HSBCF$_1$MPH
qBN-C11-2	2015Yc	i00403Gh and i07163Gh	20.01	3.70			-1.13	6.61	MARBCF$_1$
qBN-C12-1	2014Yc	i48211Gh and i08075Gh	45.21	3.45	3.12			32.42	RIL
qBN-C14-1	2015Yc	i22641Gh and i00458Gh	6.31	3.58	-2.17	-1.68		8.10	IF$_2$
	2015Bg	i22641Gh and i48509Gh	6.31	3.73			-3.05	6.87	HSBCF$_1$
qBN-C14-2	2014Yc	i35260Gh and i22218Gh	30.21	3.18		-0.34		4.80	MARBCF$_1$MPH
qBN-C14-3	2015Yc	i34963Gh and i44045Gh	50.51	3.31	-0.41			6.69	RIL
qBN-C16-1	2015Bg	i01279Gh and i01766Gh	13.51	3.71	1.94	-2.23		17.60	IF$_2$
qBN-C17-1	2015Bg	i03593Gh and i03537Gh	21.51	4.98			0.02	6.18	HSBCF$_1$
qBN-C17-2	2014Yc	i43615Gh and i03508Gh	33.41	4.48			-0.39	8.12	HSBCF$_1$
	2015Bg	i43615Gh and i03218Gh	33.41	4.45			-0.22	10.25	HSBCF$_1$
	2014Bg	i43615Gh and i03218Gh	33.41	3.15		-7.72		10.29	HSBCF$_1$MPH
qBN-C17-3	2014Yc	i03411Gh and i03527Gh	43.41	3.13		-2.56		3.76	MARBCF$_1$MPH
	2015Bg	i03527Gh and i14844Gh	44.31	3.13		0.95		9.31	MARBCF$_1$MPH
qBN-C18-1	2014Bg	i13754Gh and i29829Gh	13.31	3.33	-0.62			7.22	RIL
	2014Bg	i13145Gh and i20346Gh	16.11	4.21		0.30		10.38	IF$_2$MPH

	qBN-C18-2	2014Yc	i13133Gh and i13362Gh	58.31	4.60			-2.33	17.22	HSBCF$_1$
		2014Yc	i13133Gh and i13362Gh	58.31	4.41		-9.32		22.19	HSBCF$_1$MPH
	qBN-C19-1	2015Bg	i21172Gh and i09452Gh	47.51	4.68			-10.14	3.87	HSBCF$_1$
	qBN-C19-2	2014Yc	i21841Gh and i09665Gh	59.01	5.37		-2.80		11.38	MARBCF$_1$MPH
		2015Yc	i09665Gh and i21947Gh	61.31	5.32			-0.19	20.86	MARBCF$_1$
	qBN-C20-1	2015Bg	i11756Gh and i11734Gh	22.91	3.92		0.50		11.26	MARBCF$_1$MPH
	qBN-C20-2	2015Bg	i11735Gh and i42616Gh	30.71	3.15	2.21			10.97	RIL
	qBN-C22-1	2014Yc	i33051Gh and i42350Gh	15.41	3.29	1.21	0.38		4.99	IF$_2$
	qBN-C22-2	2014Yc	i12539Gh and i12581Gh	25.01	4.29	1.37	1.43		8.99	IF$_2$
	qBN-C22-3	2015Bg	i12810Gh and i17696Gh	40.01	3.10	1.78			13.24	RIL
		2015Bg	i12464Gh and i44682Gh	43.31	3.23	-2.69	-2.17		4.18	IF$_2$
		2015Bg	i12464Gh and i25111Gh	43.31	4.18		1.66		9.31	HSBCF$_1$MPH
		2015Yc	i12464Gh and i25111Gh	44.11	4.42		1.30		12.10	HSBCF$_1$MPH
	qBN-C23-1	2014Bg	i06287Gh and i06171Gh	22.01	3.64	2.83	1.56		15.82	IF$_2$
	qBN-C24-1	2015Yc	i04012Gh and i04423Gh	33.41	4.43		-5.21		3.90	IF$_2$MPH
	qBN-C24-2	2015Yc	i04684Gh and i15027Gh	46.61	3.72			-3.16	5.87	MARBCF$_1$
		2015Yc	i04684Gh and i15027Gh	46.61	3.95		-2.09		5.15	MARBCF$_1$MPH
	qBN-C24-3	2015Yc	i03705Gh and i03710Gh	62.61	3.95	4.46	-5.15		7.60	IF$_2$
	qBN-C25-1	2014Bg	i22495Gh and i11267Gh	41.91	4.85		-7.73		9.10	HSBCF$_1$MPH
	qBN-C25-2	2015Yc	i17145Gh and i10628Gh	74.51	3.81			3.64	22.55	MARBCF$_1$
		2015Bg	i17145Gh and i10628Gh	74.51	5.25			3.59	33.12	MARBCF$_1$
BW	qBW-C01-1	2014Yc	i33013Gh and i21390Gh	9.81	3.37	0.17	0.14		6.94	IF$_2$
	qBW-C01-2	2015Bg	i30221Gh and i45912Gh	18.31	3.77	0.00	0.57		4.50	IF$_2$
	qBW-C01-3	2014Yc	i23867Gh and i44115Gh	43.71	3.18			0.13	17.71	MARBCF$_1$
	qBW-C02-1	2014Yc	i03457Gh and i03459Gh	42.61	3.06	0.15	-2.49		4.39	IF$_2$
		2014Yc	i02758Gh and i02723Gh	44.81	3.30		1.76		24.37	HSBCF$_1$MPH

qBW-C03-1	2014Yc	i31859Gh and i05394Gh	92.31	4.28		-0.52		10.56	IF$_2$MPH
	2015Yc	i42939Gh and i34191Gh	97.91	3.94			0.94	12.25	MARBCF$_1$
qBW-C04-1	2015Yc	i50068Gb and i26515Gh	2.01	3.56			1.01	6.36	HSBCF$_1$
	2015Bg	i50068Gb and i31054Gh	4.71	3.00		-1.02		3.17	MARBCF$_1$MPH
qBW-C04-2	2015Yc	i28424Gh and i20890Gh	10.61	3.99			0.48	6.39	HSBCF$_1$
qBW-C05-1	2014Bg	i09498Gh and i22338Gh	14.11	3.38			1.14	3.14	MARBCF$_1$
qBW-C05-2	2014Yc	i09051Gh and i37479Gh	20.81	11.98			2.77	12.65	HSBCF$_1$
	2014Yc	i09051Gh and i37479Gh	20.81	8.21		1.42		3.48	HSBCF$_1$MPH
qBW-C05-3	2014Yc	i40932Gh and i40771Gh	30.51	5.74			0.03	14.88	HSBCF$_1$
qBW-C05-4	2014Yc	i34270Gh and i09003Gh	47.51	3.54		-0.67		5.95	HSBCF$_1$MPH
qBW-C06-1	2014Yc	i23722Gh and i37862Gh	44.91	3.20			0.03	12.24	MARBCF$_1$
qBW-C07-1	2014Bg	i01703Gh and i14398Gh	52.11	3.54			0.90	17.17	MARBCF$_1$
qBW-C08-1	2014Yc	i63682Gm and i37825Gh	0.01	4.14			-0.04	19.37	MARBCF$_1$
qBW-C08-2	2014Yc	i04573Gh and i32773Gh	23.91	5.14		0.20		18.57	IF$_2$MPH
qBW-C08-3	2015Yc	i42167Gh and i04772Gh	31.91	3.87			-0.12	12.19	HSBCF$_1$
	2015Bg	i30195Gh and i04557Gh	35.61	4.55			-0.14	28.14	HSBCF$_1$
	2014Yc	i40070Gh and i01126Gh	38.01	4.88		1.39		15.47	HSBCF$_1$MPH
qBW-C09-1	2014Yc	i50203Gb and i17373Gh	4.71	3.61		0.23		9.13	MARBCF$_1$MPH
qBW-C09-2	2015Yc	i46552Gh and i26826Gh	46.11	3.28	0.13			6.11	RIL
qBW-C09-3	2014Bg	i03687Gh and i02498Gh	50.61	3.17		-0.74		21.68	HSBCF$_1$MPH
qBW-C10-1	2014Yc	i38146Gh and i22401Gh	35.11	6.04	-1.25	0.94		25.86	IF$_2$
	2014Yc	i27166Gh and i45101Gh	36.01	4.47		-0.46		11.16	IF$_2$MPH
	2015Bg	i00311Gh and i40792Gh	36.81	4.27			-1.05	18.25	HSBCF$_1$
	2014Bg	i38186Gh and i26780Gh	41.21	3.73			-0.30	30.65	HSBCF$_1$
qBW-C10-2	2014Yc	i11724Gh and i22625Gh	57.61	6.17			-0.10	23.68	MARBCF$_1$
qBW-C13-1	2014Yc	i22596Gh and i12963Gh	1.11	4.80	-1.35	-1.16		6.76	IF$_2$

qBW-C13-2	2014Yc	i30934Gh and i18151Gh	19.41	5.07		1.56		32.54	HSBCF_1MPH
qBW-C13-3	2014Bg	i24929Gh and i36415Gh	30.21	3.20	-0.10	0.44		8.18	IF_2
	2014Bg	i36415Gh and i18150Gh	33.41	3.15			-0.39	3.45	HSBCF_1
qBW-C13-4	2014Yc	i32191Gh and i13740Gh	49.91	4.45			0.09	17.74	MARBCF_1
	2014Yc	i32191Gh and i13740Gh	49.91	3.34		1.22		33.09	MARBCF_1MPH
qBW-C14-1	2014Yc	i15345Gh and i00465Gh	20.91	3.51			0.34	4.35	HSBCF_1
qBW-C14-2	2014Yc	i23629Gh and i65685Gm	55.11	3.17		-0.43		6.21	IF_2MPH
qBW-C15-1	2015Bg	i64628Gm and i49465Gh	22.11	3.74			0.53	3.53	HSBCF_1
qBW-C16-1	2014Yc	i25228Gh and i01297Gh	6.01	3.39	-0.48	1.25		11.71	IF_2
qBW-C16-2	2015Yc	i51973Gb and i44137Gh	53.61	3.86		0.34		3.07	IF_2MPH
qBW-C17-1	2015Bg	i14907Gh and i14878Gh	0.01	3.01	-0.15	0.51		6.36	IF_2
qBW-C18-1	2015Yc	i13766Gh and i13145Gh	1.01	3.28	-0.13			6.11	RIL
qBW-C18-2	2015Bg	i41872Gh and i13532Gh	67.31	3.33	-0.23	0.36		11.89	IF_2
qBW-C19-1	2014Yc	i08933Gh and i28797Gh	9.61	4.38			-0.13	9.25	MARBCF_1
qBW-C19-2	2015Yc	i47122Gh and i08989Gh	27.51	3.87			1.62	5.12	MARBCF_1
qBW-C20-1	2014Yc	i11727Gh and i39228Gh	7.01	4.68		1.44		3.20	HSBCF_1MPH
	2015Bg	i39228Gh and i51628Gb	10.21	3.62	0.17			7.27	RIL
qBW-C20-2	2015Bg	i17500Gh and i17612Gh	53.91	3.57			-3.42	5.08	HSBCF_1
qBW-C20-3	2014Yc	i40369Gh and i11478Gh	58.81	3.43			0.98	4.36	HSBCF_1
	2014Yc	i11915Gh and i11478Gh	61.61	3.94		1.37		4.76	HSBCF_1MPH
qBW-C20-4	2015Bg	i11915Gh and i11478Gh	77.61	3.04			-0.46	5.42	HSBCF_1
qBW-C21-1	2014Bg	i06952Gh and i07714Gh	16.91	3.60			-1.38	7.10	HSBCF_1
qBW-C21-2	2014Bg	i47631Gh and i31769Gh	52.21	5.91			-0.69	5.05	HSBCF_1
	2015Yc	i41432Gh and i07219Gh	55.51	3.43		-0.99		6.75	HSBCF_1MPH
qBW-C24-1	2014Yc	i18793Gh and i04514Gh	71.61	5.27	-1.26	1.29		21.15	IF_2
qBW-C25-1	2014Yc	i17224Gh and i21899Gh	3.51	6.42	-0.65	1.26		17.14	IF_2

	qBW-C25-2	2015Yc	i42629Gh and i49170Gh	25.01	3.22		0.51		4.79	MARBCF$_1$MPH
	qBW-C25-3	2014Yc	i17145Gh and i10628Gh	72.51	4.55			2.09	5.29	MARBCF$_1$
	qBW-C26-1	2014Bg	i00879Gh and i32452Gh	2.01	3.07			-0.01	11.24	MARBCF$_1$
LP	qLP-C01-1	2015Yc	i02201Gh and i27043Gh	15.51	6.07		4.06		21.85	HSBCF$_1$MPH
		2014Bg	i02201Gh and i46188Gh	15.91	3.08			1.14	6.15	HSBCF$_1$
	qLP-C01-2	2014Yc	i48434Gh and i14597Gh	51.41	3.95			1.76	8.23	MARBCF$_1$
	qLP-C02-1	2014Yc	i16954Gh and i20804Gh	33.21	3.05	2.11	2.02		7.30	IF$_2$
		2014Bg	i16954Gh and i20804Gh	33.21	4.09			5.73	3.79	MARBCF$_1$
	qLP-C02-2	2014Yc	i55186Gb and i47290Gh	42.41	4.13			-0.70	9.24	MARBCF$_1$
	qLP-C02-3	2014Yc	i49488Gh and i30800Gh	79.81	3.23			0.04	16.34	MARBCF$_1$
		2014Yc	i49488Gh and i30800Gh	79.81	3.07		2.18		17.03	MARBCF$_1$MPH
	qLP-C03-1	2015Yc	i29727Gh and i47151Gh	3.51	5.06			1.65	21.99	HSBCF$_1$
	qLP-C04-1	2015Bg	i50068Gb and i31054Gh	3.71	4.03	0.62	-0.82		12.03	IF$_2$
	qLP-C04-2	2014Bg	i28424Gh and i24758Gh	11.01	4.10	0.57			9.28	RIL
		2015Bg	i28424Gh and i44542Gh	11.11	4.39	0.77	-0.54		14.35	IF$_2$
		2014Yc	i41405Gh and i24758Gh	11.41	3.25	0.52			7.63	RIL
	qLP-C04-3	2015Bg	i38159Gh and i00135Gh	17.11	4.07			0.19	12.79	MARBCF$_1$
	qLP-C05-1	2014Yc	i25837Gh and i46287Gh	1.11	4.52	-2.55	-1.60		8.01	IF$_2$
	qLP-C06-1	2015Bg	i23722Gh and i06396Gh	47.21	4.36	3.03	-2.88		22.04	IF$_2$
		2015Bg	i37862Gh and i06396Gh	47.21	5.34		-3.98		12.50	IF$_2$MPH
	qLP-C08-1	2015Yc	i00234Gh and i04570Gh	14.21	5.30	3.81	-4.24		3.41	IF$_2$
	qLP-C09-1	2015Bg	i08331Gh and i48103Gh	19.51	5.83	5.25	-5.26		18.64	IF$_2$
		2015Bg	i08331Gh and i48103Gh	19.51	3.01		-4.59		17.26	IF$_2$MPH
	qLP-C09-2	2015Bg	i39622Gh and i39335Gh	30.81	3.08	-2.88	-3.70		3.23	IF$_2$
		2014Yc	i19084Gh and i36196Gh	32.31	4.18	-3.11	3.25		31.41	IF$_2$
		2015Yc	i11678Gh and i39433Gh	35.51	5.40			0.73	13.84	MARBCF$_1$

	2015Yc	i11678Gh and i06090Gh	35.51	3.51		0.26		6.67	MARBCF$_1$MPH
qLP-C09-3	2015Yc	i35858Gh and i02498Gh	50.21	3.88	1.92			6.86	RIL
	2014Yc	i15768Gh and i29408Gh	54.31	3.41	-2.77	2.29		10.92	IF$_2$
	2015Bg	i15768Gh and i29408Gh	54.31	6.11	5.03	-5.56		19.13	IF$_2$
qLP-C09-4	2014Bg	i04801Gh and i13453Gh	72.01	3.97			-1.42	3.88	HSBCF$_1$
qLP-C10-1	2015Yc	i43940Gh and i25267Gh	2.01	7.76		2.18		5.58	IF$_2$MPH
	2015Yc	i43940Gh and i25267Gh	2.01	6.22		0.53		20.69	HSBCF$_1$MPH
qLP-C10-2	2014Yc	i38146Gh and i22401Gh	35.11	4.76	-2.30			9.90	RIL
	2015Bg	i38146Gh and i22401Gh	35.11	3.41	-1.96			6.61	RIL
	2014Bg	i27166Gh and i45101Gh	36.01	4.30		3.65		4.40	MARBCF$_1$MPH
	2015Yc	i45101Gh and i37965Gh	36.41	3.49		-3.27		3.16	HSBCF$_1$MPH
	2015Yc	i12268Gh and i11502Gh	38.01	4.62	4.51	-4.68		34.26	IF$_2$
qLP-C11-1	2014Bg	i47563Gh and i00403Gh	14.21	5.36		-3.62		4.61	IF$_2$MPH
qLP-C12-1	2014Bg	i40974Gh and i48211Gh	3.01	4.11	0.60			12.52	RIL
	2014Yc	i40974Gh and i48211Gh	4.01	3.20	0.61			10.16	RIL
qLP-C13-1	2014Yc	i13195Gh and i24557Gh	3.11	3.07	-2.33			6.67	RIL
qLP-C13-2	2015Yc	i27155Gh and i13635Gh	38.01	8.25		3.31		16.25	HSBCF$_1$MPH
	2015Yc	i36298Gh and i13635Gh	38.41	9.36		4.92		6.51	IF$_2$MPH
	2015Yc	i27155Gh and i13635Gh	38.41	5.78		2.70		3.52	MARBCF$_1$MPH
qLP-C13-3	2015Yc	i49487Gh and i35111Gh	44.41	3.91			1.36	6.31	HSBCF$_1$
	2014Bg	i13299Gh and i13049Gh	45.11	4.48			-0.12	12.64	HSBCF$_1$
qLP-C14-1	2015Yc	i24544Gh and i18840Gh	3.01	3.72			0.21	12.89	HSBCF$_1$
	2014Yc	i05482Gh and i18840Gh	3.01	3.06			0.90	15.99	MARBCF$_1$
	2014Yc	i05482Gh and i04837Gh	4.01	3.28		-4.27		25.77	MARBCF$_1$MPH
qLP-C14-2	2015Bg	i35889Gh and i32457Gh	8.11	3.82		1.24		4.15	HSBCF$_1$MPH
	2014Yc	i05711Gh and i43468Gh	9.31	3.46		-5.89		23.83	HSBCF$_1$MPH

qLP-C14-3	2015Yc	i05008Gh and i05040Gh	24.01	3.49	0.91			5.12	RIL
qLP-C14-4	2015Yc	i47441Gh and i34963Gh	48.71	4.18	-1.10			8.08	RIL
	2015Yc	i38897Gh and i15587Gh	53.81	5.99	-1.20			9.50	RIL
qLP-C14-5	2014Yc	i05035Gh and i22015Gh	73.21	3.71			-5.88	6.16	MARBCF$_1$
qLP-C15-1	2014Yc	i42293Gh and i37620Gh	35.01	3.00			-0.28	8.95	MARBCF$_1$
	2015Yc	i17844Gh and i02315Gh	36.21	5.83	3.40	-3.54		34.52	IF$_2$
qLP-C16-1	2014Yc	i30207Gh and i18361Gh	2.01	3.20	-0.77	-5.50		4.19	IF$_2$
	2014Yc	i01572Gh and i43060Gh	3.51	3.26	-3.38			7.22	RIL
qLP-C17-1	2014Bg	i03556Gh and i03433Gh	6.21	3.99			-9.09	3.48	MARBCF$_1$
	2014Bg	i27404Gh and i29679Gh	6.21	4.53		-5.57		5.21	MARBCF$_1$MPH
	2014Yc	i03433Gh and i03477Gh	6.51	3.42	-3.56			7.82	RIL
qLP-C17-2	2015Yc	i43615Gh and i14513Gh	33.41	4.30	4.48	-4.61		15.47	IF$_2$
	2015Bg	i43615Gh and i14513Gh	33.41	4.94	5.23	-5.32		30.88	IF$_2$
qLP-C18-1	2015Bg	i13754Gh and i29829Gh	8.31	3.58	-0.50			6.86	RIL
qLP-C18-2	2014Yc	i13256Gh and i13324Gh	56.51	3.49			1.02	13.30	MARBCF$_1$
	2015Yc	i00400Gh and i18042Gh	56.91	4.57		-2.41		3.84	HSBCF$_1$MPH
	2015Yc	i18042Gh and i18044Gh	57.21	3.05			-1.58	8.01	HSBCF$_1$
	2014Yc	i18045Gh and i00124Gh	57.71	3.13		8.99		5.76	MARBCF$_1$MPH
qLP-C19-1	2014Yc	i09631Gh and i16796Gh	29.61	4.43			-2.49	8.66	MARBCF$_1$
qLP-C19-2	2014Yc	i21841Gh and i09665Gh	59.01	4.35	1.65	2.25		3.45	IF$_2$
qLP-C20-1	2015Bg	i11735Gh and i24944Gh	30.71	4.58		-4.70		27.86	IF$_2$MPH
	2015Yc	i11735Gh and i42616Gh	31.11	4.33	3.12	-2.73		6.20	IF$_2$
qLP-C20-2	2015Yc	i17500Gh and i17612Gh	53.11	4.07			3.99	3.86	HSBCF$_1$
	2014Yc	i17500Gh and i12060Gh	53.91	4.10			-1.51	10.47	MARBCF$_1$
	2014Yc	i17500Gh and i12060Gh	53.91	3.04		1.54		9.51	MARBCF$_1$MPH
qLP-C21-1	2014Bg	i19359Gh and i16079Gh	45.01	5.95			0.64	13.13	MARBCF$_1$

	qLP-C22-1	2014Bg	i12928Gh and i20168Gh	3.51	3.66			-0.45	8.98	MARBCF$_1$
	qLP-C22-2	2015Yc	i20168Gh and i33089Gh	10.71	3.60			1.29	21.29	HSBCF$_1$
	qLP-C22-3	2014Yc	i45635Gh and i12927Gh	16.61	3.01	-2.36			7.04	RIL
		2014Yc	i35205Gh and i12927Gh	16.71	4.87	-1.10	-3.46		3.70	IF$_2$
	qLP-C24-1	2015Yc	i41754Gh and i04544Gh	21.31	7.35	-4.35	-5.05		4.71	IF$_2$
	qLP-C24-2	2015Yc	i31637Gh and i15169Gh	40.11	6.57		3.54		32.45	HSBCF$_1$MPH
		2015Yc	i48423Gh and i15169Gh	40.11	4.18		2.19		12.18	MARBCF$_1$MPH
	qLP-C25-1	2015Yc	i11321Gh and i23244Gh	2.01	3.34		-3.75		3.47	IF$_2$MPH
		2014Yc	i23207Gh and i23244Gh	2.11	3.97	-0.90	-5.31		3.00	IF$_2$
	qLP-C25-2	2015Yc	i00735Gh and i41210Gh	6.01	5.77	3.47	-3.30		12.71	IF$_2$
		2015Bg	i00735Gh and i41210Gh	6.01	5.79	4.10	-4.49		24.92	IF$_2$
		2015Bg	i00735Gh and i41210Gh	6.01	6.45		-4.09		17.86	IF$_2$MPH
	qLP-C25-3	2015Bg	i18176Gh and i11465Gh	32.51	5.50	5.12	-5.20		9.37	IF$_2$
		2015Bg	i18176Gh and i11465Gh	32.51	6.16		-4.30		8.75	IF$_2$MPH
		2015Bg	i18176Gh and i11465Gh	33.51	3.48	1.25			12.88	RIL
	qLP-C26-1	2015Yc	i00879Gh and i32452Gh	2.51	3.30	-0.94			5.03	RIL
	qLP-C26-2	2015Yc	i23175Gh and i08243Gh	53.11	4.54			-0.95	11.25	HSBCF$_1$
	qLP-C26-3	2015Yc	i16424Gh and i27141Gh	60.91	5.10	0.29	6.09		17.91	IF$_2$
SY	qSY-C01-1	2015Yc	i02201Gh and i27043Gh	14.51	3.61			223.35	7.60	HSBCF$_1$
	qSY-C01-2	2014Bg	i53496Gb and i02994Gh	24.91	3.04	254.33			11.40	RIL
	qSY-C02-1	2014Yc	i02712Gh and i20804Gh	26.31	3.11	277.37			12.56	RIL
		2015Yc	i02712Gh and i20804Gh	29.21	4.46			-39.21	26.01	MARBCF$_1$
	qSY-C02-2	2014Bg	i31133Gh and i00219Gh	42.81	4.69		-550.08		12.27	HSBCF$_1$MPH
		2015Bg	i14776Gh and i16398Gh	43.91	3.50		-189.62		14.23	IF$_2$MPH
		2015Bg	i14776Gh and i16398Gh	43.91	3.70			59.82	17.84	HSBCF$_1$
		2015Yc	i02758Gh and i02723Gh	44.81	3.15			271.49	6.59	MARBCF$_1$

qSY-C02-3	2014Yc	i02328Gh and i00463Gh	53.81	3.35			216.40	4.25	HSBCF$_1$
qSY-C02-4	2015Bg	i30800Gh and i09654Gh	89.51	5.23		269.17		23.28	HSBCF$_1$MPH
	2015Yc	i30800Gh and i07717Gh	89.51	3.51		172.03		29.39	MARBCF$_1$MPH
qSY-C03-1	2014Bg	i32361Gh and i27670Gh	9.81	4.13		-84.09		4.95	HSBCF$_1$MPH
qSY-C04-1	2015Bg	i50068Gb and i31054Gh	4.71	3.32		-146.44		5.19	MARBCF$_1$MPH
qSY-C05-1	2015Bg	i46287Gh and i49326Gh	5.01	3.12		-307.58		23.03	IF$_2$MPH
	2015Bg	i46287Gh and i49326Gh	5.01	3.91			68.98	21.10	HSBCF$_1$
	2014Bg	i43315Gh and i43323Gh	5.81	4.28		-547.58		22.17	HSBCF$_1$MPH
qSY-C05-2	2015Bg	i25259Gh and i41875Gh	12.11	3.77			77.24	16.59	HSBCF$_1$
	2015Bg	i25259Gh and i41875Gh	12.11	4.02		-186.11		18.04	HSBCF$_1$MPH
qSY-C05-3	2014Bg	i37479Gh and i08984Gh	27.81	4.15	-49.74	272.65		10.35	IF$_2$
qSY-C06-1	2015Bg	i19214Gh and i43320Gh	15.81	3.27			-377.31	4.19	MARBCF$_1$
qSY-C06-2	2015Bg	i21566Gh and i06526Gh	23.31	4.40			55.38	9.96	MARBCF$_1$
	2015Bg	i21566Gh and i06526Gh	23.61	4.23		100.31		9.91	MARBCF$_1$MPH
qSY-C06-3	2015Bg	i14061Gh and i15830Gh	30.81	4.11		-154.15		26.82	HSBCF$_1$MPH
qSY-C07-1	2014Bg	i01651Gh and i01602Gh	49.81	3.87		-544.23		11.57	HSBCF$_1$MPH
	2015Bg	i01651Gh and i01602Gh	49.81	4.00		455.35		6.16	HSBCF$_1$MPH
qSY-C08-1	2015Bg	i04772Gh and i45703Gh	32.31	3.88			-99.64	12.82	HSBCF$_1$
	2015Bg	i04772Gh and i45703Gh	32.31	5.03		-223.25		21.84	HSBCF$_1$MPH
qSY-C09-1	2015Bg	i05810Gh and i05840Gh	28.31	4.51		170.48		17.36	MARBCF$_1$MPH
	2015Bg	i39622Gh and i00142Gh	30.51	7.07		311.56		19.78	HSBCF$_1$MPH
	2015Bg	i38554Gh and i40112Gh	31.81	3.45			-34.62	21.28	HSBCF$_1$
qSY-C10-1	2015Yc	i43940Gh and i30274Gh	13.01	4.73			43.88	13.81	MARBCF$_1$
	2015Bg	i43940Gh and i30274Gh	13.21	3.26		71.82		9.04	MARBCF$_1$MPH
	2015Yc	i43940Gh and i30274Gh	14.21	3.54		92.45		8.32	MARBCF$_1$MPH
qSY-C10-2	2014Yc	i11724Gh and i22625Gh	57.61	3.38	275.08			12.38	RIL

qSY-C13-1	2015Yc	i13077Gh and i32868Gh	4.71	3.08	-115.29			7.49	RIL
qSY-C13-2	2014Bg	i30934Gh and i18151Gh	19.41	4.40	170.19			17.83	RIL
qSY-C13-3	2014Bg	i23966Gh and i29670Gh	29.11	5.17			-533.71	7.68	MARBCF$_1$
qSY-C13-4	2014Bg	i27775Gh and i36298Gh	37.81	3.48			-36.62	9.44	MARBCF$_1$
	2015Yc	i42046Gh and i38620Gh	40.91	3.12		-2.17		8.48	MARBCF$_1$MPH
qSY-C13-5	2014Bg	i26788Gh and i25788Gh	49.91	4.40	213.53			15.61	RIL
	2014Bg	i32191Gh and i13740Gh	49.91	3.27		-544.87		11.14	HSBCF$_1$MPH
qSY-C13-6	2015Bg	i25007Gh and i12964Gh	55.21	4.12		-342.24		11.84	IF$_2$MPH
qSY-C14-1	2014Yc	i43400Gh and i05130Gh	35.51	3.38	268.93			11.42	RIL
qSY-C14-2	2015Bg	i49621Gh and i38897Gh	53.41	3.47		-156.54		5.65	MARBCF$_1$MPH
qSY-C15-1	2014Yc	i02469Gh and i02538Gh	15.61	3.43	272.48			12.17	RIL
qSY-C16-1	2015Bg	i25908Gh and i43850Gh	3.01	4.03			364.20	4.23	HSBCF$_1$
	2014Bg	i01592Gh and i47454Gh	3.11	3.45		-564.13		15.03	HSBCF$_1$MPH
	2015Bg	i47454Gh and i29124Gh	3.41	4.85		203.19		4.61	HSBCF$_1$MPH
qSY-C16-2	2015Yc	i01639Gh and i49092Gh	26.61	3.58			-172.92	23.78	MARBCF$_1$
	2015Yc	i01639Gh and i20393Gh	26.61	3.09		-88.76		13.07	MARBCF$_1$MPH
	2015Bg	i01639Gh and i20393Gh	26.61	3.04		-96.66		12.75	MARBCF$_1$MPH
	2014Bg	i01374Gh and i24534Gh	28.31	3.12		129.60		6.74	IF$_2$MPH
qSY-C17-1	2014Bg	i03447Gh and i03434Gh	6.01	4.62		-624.26		14.76	HSBCF$_1$MPH
qSY-C18-1	2014Yc	i13754Gh and i29829Gh	3.91	3.79		-9.26		13.47	HSBCF$_1$MPH
qSY-C18-2	2015Bg	i20381Gh and i13851Gh	28.31	4.54	-173.67	-113.54		6.19	IF$_2$
	2015Yc	i20381Gh and i32883Gh	28.31	5.13		-104.65		8.64	IF$_2$MPH
	2015Bg	i20381Gh and i32883Gh	28.31	3.15		-11.08		8.92	IF$_2$MPH
qSY-C18-3	2014Bg	i13007Gh and i13362Gh	58.41	5.47		-479.69		34.83	HSBCF$_1$MPH
	2015Bg	i24219Gh and i17887Gh	61.21	3.55			292.42	3.40	HSBCF$_1$
qSY-C19-1	2014Yc	i41523Gh and i09665Gh	58.01	4.11	287.80			13.94	RIL

	2015Yc	i09665Gh and i21947Gh	60.31	4.44			-206.71	18.08	MARBCF$_1$
	2015Yc	i09665Gh and i21947Gh	60.31	5.00		-121.76		6.75	MARBCF$_1$MPH
	2015Bg	i09665Gh and i21947Gh	61.31	4.15		-105.97		4.39	MARBCF$_1$MPH
qSY-C20-1	2014Yc	i11611Gh and i11698Gh	13.11	3.30			136.80	4.33	HSBCF$_1$
qSY-C20-2	2014Yc	i11735Gh and i44450Gh	30.71	3.53	273.91			12.30	RIL
qSY-C21-1	2014Bg	i07262Gh and i07714Gh	7.91	3.16		-566.78		11.57	HSBCF$_1$MPH
	2015Bg	i06952Gh and i07714Gh	10.91	6.60		-135.16		13.62	HSBCF$_1$MPH
qSY-C21-2	2015Bg	i06952Gh and i38909Gh	18.91	4.87		301.98		32.18	HSBCF$_1$MPH
qSY-C21-3	2014Yc	i22400Gh and i07219Gh	53.31	3.80			108.24	5.14	MARBCF$_1$
qSY-C22-1	2015Bg	i28912Gh and i12813Gh	13.31	3.73			90.65	13.45	HSBCF$_1$
	2014Bg	i12812Gh and i43257Gh	13.91	4.30		-519.71		18.28	HSBCF$_1$MPH
qSY-C22-2	2014Bg	i17853Gh and i12538Gh	31.31	3.83	-181.30	-105.67		9.89	IF$_2$
	2014Yc	i17715Gh and i12538Gh	31.51	4.08			156.03	3.97	HSBCF$_1$
qSY-C23-1	2015Bg	i06162Gh and i33428Gh	27.11	4.03		-283.61		20.86	HSBCF$_1$MPH
	2015Bg	i05800Gh and i26750Gh	28.91	3.51			-15.76	33.40	HSBCF$_1$
qSY-C24-1	2015Yc	i41754Gh and i04544Gh	21.31	3.41		197.05		5.14	MARBCF$_1$MPH
qSY-C24-2	2014Bg	i04592Gh and i20725Gh	72.01	4.24		-498.78		15.41	HSBCF$_1$MPH
	2015Bg	i01121Gh and i04503Gh	72.91	3.43		-229.14		25.55	IF$_2$MPH
	2015Bg	i01121Gh and i04503Gh	72.91	3.55			39.58	18.96	HSBCF$_1$
	2015Bg	i01121Gh and i04503Gh	72.91	4.95		-138.26		21.38	HSBCF$_1$MPH
qSY-C25-1	2014Bg	i00735Gh and i42629Gh	9.91	4.03			-281.42	3.66	HSBCF$_1$
qSY-C25-2	2015Bg	i21894Gh and i10599Gh	45.81	3.49	29.53			8.12	RIL
qSY-C25-3	2015Yc	i17145Gh and i10628Gh	73.51	3.39		260.99		24.33	MARBCF$_1$MPH
qSY-C26-1	2014Bg	i08062Gh and i38136Gh	9.51	3.04		-2.58		7.33	MARBCF$_1$MPH
qSY-C26-2	2014Yc	i37251Gh and i01105Gh	20.11	3.62	48.21			8.39	RIL
	2014Bg	i37251Gh and i16418Gh	20.11	4.30	50.19			9.78	RIL

LY	qLY-C01-1	2014Bg	i02295Gh and i21520Gh	39.91	3.02			-49.39	28.19	MARBCF$_1$
	qLY-C01-2	2015Yc	i25005Gh and i38924Gh	49.11	3.12		-6.90		9.85	HSBCF$_1$MPH
	qLY-C02-1	2015Yc	i02761Gh and i16954Gh	25.11	4.54		25.12		34.06	HSBCF$_1$MPH
	qLY-C02-2	2015Bg	i14776Gh and i16398Gh	43.91	4.44			16.71	19.90	HSBCF$_1$
		2014Bg	i02758Gh and i02723Gh	44.81	4.18		334.79		5.99	HSBCF$_1$MPH
		2015Yc	i05624Gh and i46092Gh	45.11	3.75		68.35		3.88	HSBCF$_1$MPH
	qLY-C02-3	2014Bg	i14841Gh and i30800Gh	80.51	3.71			-45.71	3.60	MARBCF$_1$
		2015Yc	i38985Gh and i30800Gh	82.51	3.04		-37.93		24.62	IF$_2$MPH
	qLY-C05-1	2015Bg	i25259Gh and i41875Gh	12.11	4.36			14.14	19.82	HSBCF$_1$
		2015Bg	i25259Gh and i41875Gh	12.11	4.34		-88.67		21.40	HSBCF$_1$MPH
		2014Bg	i44670Gh and i45091Gh	12.31	3.16			65.38	11.20	MARBCF$_1$
	qLY-C05-2	2014Bg	i53001Gb and i08984Gh	27.81	3.27	-19.77	112.73		8.28	IF$_2$
		2015Yc	i53001Gb and i08984Gh	29.81	3.55			7.49	11.21	MARBCF$_1$
	qLY-C06-1	2015Bg	i19214Gh and i43320Gh	15.81	3.47			-161.58	4.66	MARBCF$_1$
	qLY-C06-2	2015Bg	i21566Gh and i06526Gh	23.31	3.65		35.77		8.91	MARBCF$_1$MPH
		2014Yc	i21566Gh and i06526Gh	23.61	3.33	-28.59	11.41		11.74	IF$_2$
		2015Bg	i26917Gh and i14061Gh	25.61	4.60			-88.68	6.79	MARBCF$_1$
		2015Bg	i14061Gh and i15830Gh	29.81	3.24			6.93	17.20	HSBCF$_1$
	qLY-C06-3	2015Bg	i23722Gh and i06396Gh	45.91	4.49			-8.19	3.26	HSBCF$_1$
	qLY-C07-1	2014Yc	i22038Gh and i35445Gh	44.21	3.06	66.98			14.58	RIL
	qLY-C07-2	2014Bg	i01602Gh and i21938Gh	51.31	3.04			115.46	9.39	MARBCF$_1$
		2015Bg	i01611Gh and i21938Gh	51.31	3.32		89.03		4.02	MARBCF$_1$MPH
	qLY-C08-1	2015Bg	i00234Gh and i04570Gh	14.21	3.73			1.03	13.21	HSBCF$_1$
	qLY-C08-2	2014Bg	i43992Gh and i15119Gh	37.11	3.84		-106.43		27.54	HSBCF$_1$MPH
	qLY-C09-1	2015Bg	i39622Gh and i00142Gh	30.51	4.44		99.10		15.45	HSBCF$_1$MPH
		2015Bg	i38554Gh and i40112Gh	31.81	4.38			-15.83	24.26	HSBCF$_1$

qLY-C09-2	2014Yc	i15489Gh and i52208Gb	58.01	4.95	-3.41	68.17		6.84	IF$_2$
qLY-C11-1	2014Bg	i07163Gh and i40251Gh	26.21	3.03		-68.38		22.72	MARBCF$_1$MPH
qLY-C13-1	2015Yc	i13077Gh and i32868Gh	4.71	3.44	-37.56			7.14	RIL
qLY-C13-2	2014Bg	i30934Gh and i18151Gh	18.41	4.07	51.87			18.68	RIL
qLY-C13-3	2014Bg	i23966Gh and i29670Gh	29.11	4.74			-233.59	5.41	MARBCF$_1$
qLY-C13-4	2014Bg	i26788Gh and i13740Gh	49.91	3.87	85.90			12.80	RIL
qLY-C14-1	2014Yc	i43400Gh and i05130Gh	35.51	3.21	102.08			11.85	RIL
qLY-C14-2	2015Bg	i49621Gh and i38897Gh	53.41	3.96		-63.18		4.23	MARBCF$_1$MPH
qLY-C15-1	2015Bg	i02955Gh and i02280Gh	4.01	3.14		-128.21		24.37	IF$_2$MPH
qLY-C15-2	2014Yc	i02469Gh and i02538Gh	15.61	3.04	100.57			11.64	RIL
qLY-C16-1	2015Bg	i01279Gh and i14406Gh	13.51	4.13		-70.74		31.44	HSBCF$_1$MPH
qLY-C16-2	2015Yc	i50349Gb and i21863Gh	44.41	3.60			142.69	3.40	MARBCF$_1$
qLY-C17-1	2014Yc	i14812Gh and i03541Gh	26.21	3.09		-58.36		3.57	MARBCF$_1$MPH
qLY-C18-1	2015Bg	i13146Gh and i13454Gh	26.11	3.86	-86.28	-65.73		4.88	IF$_2$
	2015Yc	i20381Gh and i32883Gh	28.31	4.42		-37.01		7.52	IF$_2$MPH
qLY-C18-2	2014Bg	i45991Gh and i13081Gh	97.21	3.38		-152.22		3.36	IF$_2$MPH
qLY-C19-1	2014Yc	i55376Gb and i09636Gh	26.51	4.26		-97.06		3.59	IF$_2$MPH
	2015Yc	i08945Gh and i09631Gh	29.21	3.72		76.55		3.93	HSBCF$_1$MPH
qLY-C19-2	2014Bg	i08789Gh and i16573Gh	54.81	4.60		62.64		4.06	MARBCF$_1$MPH
	2014Yc	i41523Gh and i09665Gh	58.01	3.10	100.46			11.83	RIL
	2015Yc	i09665Gh and i21947Gh	60.31	3.03		-35.79		8.10	MARBCF$_1$MPH
qLY-C21-1	2014Yc	i06952Gh and i07714Gh	16.91	5.49			-157.23	27.28	MARBCF$_1$
	2014Yc	i07714Gh and i38909Gh	21.11	4.90	-57.59	-141.76		18.43	IF$_2$
	2014Bg	i07714Gh and i47711Gh	24.51	3.49	29.83	-37.46		16.40	IF$_2$
qLY-C21-2	2014Yc	i23301Gh and i07219Gh	53.31	4.40			47.50	5.29	MARBCF$_1$
qLY-C21-3	2014Yc	i22642Gh and i33529Gh	64.91	3.14			-200.39	3.15	MARBCF$_1$

	2014Yc	i22991Gh and i43642Gh	68.01	3.28		-101.01		4.25	MARBCF$_1$MPH
qLY-C22-1	2015Bg	i30763Gh and i17698Gh	11.61	4.08			100.83	3.62	HSBCF$_1$
	2015Yc	i39918Gh and i17698Gh	11.61	4.92		173.76		28.91	HSBCF$_1$MPH
	2014Bg	i12449Gh and i12813Gh	13.21	4.56		162.91		4.47	HSBCF$_1$MPH
qLY-C22-2	2014Bg	i17853Gh and i17715Gh	31.31	4.42	-81.09	-45.52		11.62	IF$_2$
	2014Yc	i17715Gh and i12538Gh	31.51	4.47			64.15	4.17	HSBCF$_1$
	2014Yc	i17715Gh and i54007Gb	31.51	4.84		45.66		4.06	HSBCF$_1$MPH
qLY-C22-3	2014Bg	i44682Gh and i25111Gh	47.81	3.20	33.84	-31.15		18.20	IF$_2$
qLY-C23-1	2015Bg	i06162Gh and i33428Gh	27.11	4.57			-22.74	19.88	HSBCF$_1$
qLY-C24-1	2015Bg	i40219Gh and i04607Gh	71.21	4.92			-39.35	19.73	HSBCF$_1$
	2015Bg	i01121Gh and i04503Gh	72.91	4.82		-75.11		24.54	HSBCF$_1$MPH
qLY-C25-1	2014Bg	i00735Gh and i42629Gh	10.91	3.78			-103.57	3.06	HSBCF$_1$
	2014Bg	i00735Gh and i42629Gh	10.91	3.32		-37.41		4.65	HSBCF$_1$MPH
qLY-C25-2	2014Yc	i11397Gh and i39910Gh	34.51	3.51		-75.23		4.17	IF$_2$MPH
qLY-C25-3	2015Bg	i37080Gh and i10599Gh	45.81	4.93	13.72			11.77	RIL
qLY-C25-4	2015Yc	i17145Gh and i10628Gh	74.51	3.13		-61.75		12.29	MARBCF$_1$MPH
qLY-C26-1	2015Bg	i00879Gh and i32452Gh	2.51	3.26		39.66		3.15	HSBCF$_1$MPH
qLY-C26-2	2014Yc	i37251Gh and i01105Gh	20.11	3.05	17.08			7.21	RIL
	2014Bg	i37251Gh and i01105Gh	20.11	3.85	19.27			8.84	RIL
qLY-C26-3	2014Yc	i08565Gh and i08578Gh	39.31	3.41	53.18	-91.71		12.77	IF$_2$

[a] FB: number of fruit branches per plant; BN: number of bolls per plant; BW: boll weight; LP: lint percentage; SY: seed cotton yield; LY: lint yield

[b] 2014Yc: Yacheng, Hainan Province in 2014; 2014Bg: Baogang, Hainan Province in 2014; 2015Yc: Yacheng, Hainan Province in 2015; 2015Bg: Baogang, Hainan Province in 2015

[c] Position of QTL located on chromosome: as cM distance from the top of each chromosome

[d] A LOD threshold was used for declaration of QTL based on 1000 permutations at as significance level of 0.01

[e] The genetic expectation of a QTL effect obtained is the additive effect (A) when estimated from the RILs, the additive effect (A) and dominant effect (D) the IF2s, the additive and dominance effects (A+D) from the BCF$_1$s, and the dominance effect (D) from the MPH values

[f] Phenotypic variance explained by QTL
