# Supplementary material for: Genetic basis of heterosis for yield and yield components explored by QTL mapping across four genetic populations in upland cotton
Source: BMC Genomics. 2018 Dec 12;19:910. doi: 10.1186/s12864-018-5289-2 (PMC6292039; doi:10.1186/s12864-018-5289-2)
Supplement: Supplementary file 7 — Table S6. Main effects and environmental interactions detected for yield and yield components in RIL, IF2 and two BCF1 datasets using the ICIM method. (PDF 404 kb) [file 12864_2018_5289_MOESM7_ESM.pdf]

Table S6 Main effects and environmental interactions detected for yield and yield components in RIL, IF_2 and two BCF_1 populations using the ICIM method

Traits[a]	m-QTL	Chr.	Position[b]	Flanking markers[c]	LOD[d]	PV[e]	PV(A)[e]	PV(AE)[e]
				**RIL population**				
FB	RmaqFB-C02-1	2	47	i46092Gh-i02729Gh	3.64	2.44	2.29	0.15
	RmaqFB-C05-1	5	28	i53001Gb-i08984Gh	3.51	2.38	2.26	0.11
	RmaqFB-C07-1	7	39	i29789Gh-i42914Gh	3.50	2.03	1.00	1.03
	RmaqFB-C09-1	9	61	i05874Gh-i05194Gh	4.03	2.65	2.61	0.04
	RmaqFB-C14-1	14	15	i15343Gh-i31037Gh	3.60	2.67	1.82	0.85
	RmaqFB-C16-1	16	19	i14319Gh-i22603Gh	3.82	2.97	2.81	0.16
	RmaqFB-C24-1	24	50	i38401Gh-i04575Gh	3.56	2.38	1.42	0.97
BN	RmaqBN-C01-1	1	41	i37932Gh-i31455Gh	3.88	1.73	1.09	0.65
	RmaqBN-C03-1	3	1	i05699Gh-i44203Gh	4.17	2.12	1.48	0.64
	RmaqBN-C18-1	18	10	i13145Gh-i29829Gh	4.28	1.97	0.65	1.32
	RmaqBN-C18-2	18	90	i64918Gm-i45991Gh	3.81	1.22	1.18	0.03
	RmaqBN-C19-1	19	54	i10464Gh-i09386Gh	4.60	1.71	1.58	0.13
	RmaqBN-C24-1	24	72	i30899Gh-i20725Gh	3.93	1.24	1.17	0.08
BW	RmaqBW-C01-1	1	22	i14519Gh-i38692Gh	3.77	2.49	2.09	0.40
	RmaqBW-C02-1	2	48	i49496Gh-i39133Gh	4.38	2.53	1.97	0.56
	RmaqBW-C09-1	9	46	i24387Gh-i36372Gh	5.30	3.54	2.97	0.57
	RmaqBW-C15-1	15	9	i35552Gh-i02483Gh	4.55	3.09	2.69	0.40
	RmaqBW-C16-1	16	21	i24193Gh-i00389Gh	3.83	2.29	0.97	1.32
	RmaqBW-C18-1	18	0	i13766Gh-i13754Gh	3.68	2.78	1.54	1.24
	RmaqBW-C19-1	19	41	i16786Gh-i09475Gh	4.34	3.36	1.47	1.89
	RmaqBW-C24-1	24	40	i48423Gh-i43942Gh	4.67	3.23	3.21	0.02
	RmaqBW-C25-1	25	38	i11359Gh-i33416Gh	6.50	4.81	3.81	0.99
LP	RmaqLP-C02-1	2	53	i02328Gh-i02246Gh	5.81	3.36	2.61	0.75
	RmaqLP-C04-1	4	12	**i24786Gh-i41322Gh**	4.13	2.69	1.70	1.00
	RmaqLP-C05-1	5	58	i00180Gh-i20652Gh	3.67	2.39	2.33	0.06
	RmaqLP-C06-1	6	24	i26917Gh-i06526Gh	3.74	2.44	1.99	0.45
	RmaqLP-C06-2	6	28	i14061Gh-i34827Gh	8.57	5.79	5.13	0.66
	RmaqLP-C07-1	7	31	i26820Gh-i66007Ga	4.66	11.18	2.77	8.41
	RmaqLP-C08-1	8	15	i04570Gh-i04506Gh	5.48	14.18	4.39	9.79
	RmaqLP-C09-1	9	61	i05874Gh-i05194Gh	5.09	10.37	1.45	8.93
	RmaqLP-C10-1	10	53	i33011Gh-i22938Gh	3.95	2.46	2.03	0.42
	RmaqLP-C12-1	12	0	**i40974Gh-i48211Gh**	5.27	3.37	2.19	1.18
	RmaqLP-C13-1	13	9	i45163Gh-i30934Gh	4.01	2.51	1.81	0.70
	RmaqLP-C13-2	13	39	i20297Gh-i32206Gh	3.56	2.42	1.77	0.65
	RmaqLP-C14-1	14	36	i33821Gh-i05130Gh	3.68	1.91	1.04	0.87
	RmaqLP-C14-2	14	48	i23349Gh-i34963Gh	4.72	2.67	1.96	0.71
	RmaqLP-C16-1	16	35	i01640Gh-i00384Gh	3.83	3.02	2.67	0.35
	RmaqLP-C18-1	18	6	**i13145Gh-i29829Gh**	4.68	2.76	0.98	1.78

	RmaqLP-C20-1	20	36	i35292Gh-i31968Gh	6.21	4.58	0.42	4.16
	RmaqLP-C25-1	25	26	i27022Gh-i11449Gh	3.78	2.75	0.25	2.50
SY	RmaqSY-C07-1	7	24	i01453Gh-i33174Gh	6.48	4.61	3.95	0.66
	RmaqSY-C26-1	26	25	i08562Gh-i49188Gh	4.99	3.60	3.38	0.22
LY	RmaqLY-C7-1	7	24	i01453Gh-i33174Gh	7.29	6.16	4.93	1.23
	RmaqLY-C13-1	13	32	i32083Gh-i36415Gh	4.14	3.58	2.08	1.50
	RmaqLY-C13-2	13	41	i42844Gh-i38620Gh	3.62	2.86	2.49	0.37
	RmaqLY-C17-1	17	6	i27404Gh-i03434Gh	3.53	3.14	2.17	0.97
	RmaqLY-C26-1	26	20	i37251Gh-i23249Gh	3.69	3.19	2.86	0.33
	RmaqLY-C26-2	26	25	i08562Gh-i49188Gh	4.04	3.24	3.10	0.14
				**IF_2 population**				
FB	ImaqFB-C01-1	1	10	i53010Gb-i21390Gh	6.09	2.65	0.57	2.08
	ImaqFB-C02-1	2	62	i02432Gh-i14623Gh	4.79	2.13	0.50	1.63
	ImaqFB-C05-1	5	18	i35761Gh-i09052Gh	5.95	2.23	0.01	2.23
	ImaqFB-C14-1	14	24	i05007Gh-i34657Gh	4.54	2.16	1.25	0.91
	ImaqFB-C19-1	19	23	i09057Gh-i31823Gh	4.77	2.26	0.54	1.72
	ImaqFB-C22-1	22	38	i12810Gh-i17697Gh	4.47	1.85	0.54	1.31
BN	ImaqBN-C01-1	1	11	i31992Gh-i41337Gh	4.44	1.62	0.58	1.03
	ImaqBN-C05-1	5	5	i43315Gh-i49326Gh	4.86	2.03	0.92	1.10
	ImaqBN-C13-1	13	14	i30934Gh-i18151Gh	4.48	1.84	1.49	0.35
	ImaqBN-C13-2	13	44	i49771Gh-i49487Gh	5.57	2.20	1.86	0.34
BW	ImaqBW-C01-1	1	10	i53010Gb-i21390Gh	5.63	1.51	1.14	0.37
	ImaqBW-C01-2	1	18	i30221Gh-i37135Gh	6.73	3.20	2.45	0.75
	ImaqBW-C09-1	9	99	i13502Gh-i25039Gh	6.20	1.59	0.32	1.27
	ImaqBW-C10-1	10	35	i38146Gh-i12097Gh	10.78	3.06	1.42	1.64
	ImaqBW-C13-1	13	36	i23838Gh-i21879Gh	4.66	1.69	0.13	1.56
	ImaqBW-C14-1	14	31	i21167Gh-i44046Gh	6.67	2.20	1.84	0.36
	ImaqBW-C16-1	16	29	i21774Gh-i33622Gh	4.79	1.56	0.60	0.97
	ImaqBW-C25-1	25	47	i19984Gh-i19988Gh	4.64	0.71	0.19	0.52
LP	ImaqLP-C04-1	4	11	i45240Gh-i20890Gh	5.25	2.80	1.65	1.15
	ImaqLP-C08-1	8	38	i04524Gh-i01126Gh	4.82	1.77	1.25	0.52
	ImaqLP-C14-1	14	31	i21167Gh-i44046Gh	4.47	1.86	1.28	0.58
	ImaqLP-C19-1	19	26	i27871Gh-i09035Gh	14.08	2.06	0.11	1.96
	ImaqLP-C20-1	20	6	i46485Gh-i11727Gh	5.76	2.01	0.32	1.70
	ImaqLP-C20-1	20	32	i24944Gh-i42616Gh	7.35	1.41	0.78	0.63
	ImaqLP-C21-1	21	23	i07714Gh-i38909Gh	4.55	1.70	0.35	1.35
	ImaqLP-C25-1	25	27	**i49170Gh-i11464Gh**	4.73	1.90	1.58	0.32
SY	ImaqSY-C02-1	2	44	i02258Gh-i16398Gh	5.17	1.09	0.12	0.96
	ImaqSY-C02-2	2	56	i00463Gh-i25397Gh	4.43	1.67	1.05	0.61
	ImaqSY-C02-3	2	81	i38985Gh-i30800Gh	4.67	1.43	1.15	0.28
	ImaqSY-C03-1	3	4	**i47151Gh-i39378Gh**	4.95	1.68	0.71	0.97
	ImaqSY-C05-1	5	5	i43315Gh-i49326Gh	6.44	1.72	0.20	1.52

	ImaqSY-C07-1	7	38	**i21721Gh-i39888Gh**	4.44	1.34	0.59	0.75
	ImaqSY-C11-1	11	5	i33855Gh-i07729Gh	4.50	1.52	0.69	0.83
	ImaqSY-C13-1	13	24	i13079Gh-i36296Gh	4.88	1.38	1.37	0.01
	ImaqSY-C13-2	13	44	i49771Gh-i49487Gh	4.85	1.28	1.19	0.10
	ImaqSY-C13-3	13	56	i46408Gh-i12964Gh	4.54	1.36	1.23	0.13
	ImaqSY-C14-1	14	59	i37747Gh-i22568Gh	4.59	0.55	0.40	0.15
	ImaqSY-C15-1	15	26	i25137Gh-i23643Gh	5.77	2.11	1.51	0.60
	ImaqSY-C16-1	16	16	i01766Gh-i00144Gh	5.15	1.79	1.48	0.31
	ImaqSY-C16-2	16	19	i14319Gh-i22603Gh	6.56	2.00	1.75	0.25
	ImaqSY-C18-1	18	61	i24219Gh-i12999Gh	6.09	1.65	0.93	0.72
	ImaqSY-C18-2	18	107	i45991Gh-i13081Gh	5.59	1.52	1.48	0.04
	ImaqSY-C21-1	21	24	i22367Gh-i47711Gh	4.86	1.63	0.93	0.69
	ImaqSY-C23-1	23	28	i45538Gh-i05805Gh	5.02	0.82	0.13	0.69
	ImaqSY-C24-1	24	71	i44566Gh-i04593Gh	4.82	1.16	0.69	0.47
LY	ImaqLY-C02-1	2	44	i02258Gh-i16398Gh	5.10	1.39	0.21	1.18
	ImaqLY-C05-1	5	5	i43315Gh-i49326Gh	6.36	2.13	0.29	1.84
	ImaqLY-C07-1	7	38	i21721Gh-i39888Gh	4.50	1.73	0.74	0.99
	ImaqLY-C13-1	13	44	i49771Gh-i49487Gh	6.34	2.21	1.97	0.24
	ImaqLY-C15-1	15	23	**i34122Gh-i49465Gh**	4.98	2.00	1.24	0.77
	ImaqLY-C15-2	15	26	i25137Gh-i23643Gh	7.47	3.54	2.43	1.11
	ImaqLY-C16-1	16	19	i14319Gh-i22603Gh	4.72	1.90	1.41	0.49
	ImaqLY-C16-1	16	41	i45950Gh-i36953Gh	4.50	1.79	0.93	0.86
	ImaqLY-C18-1	18	61	i24219Gh-i12999Gh	4.85	1.65	0.76	0.89
	ImaqLY-C18-2	18	70	i49258Gh-i13532Gh	4.58	1.31	0.29	1.02
	ImaqLY-C21-1	21	24	i22367Gh-i47711Gh	4.77	2.24	1.09	1.15
	ImaqLY-C23-1	23	28	i45538Gh-i05805Gh	4.79	1.10	0.13	0.98
	ImaqLY-C24-1	24	0	i04792Gh-i43386Gh	4.93	1.99	0.03	1.96
	ImaqLY-C24-2	24	22	i45941Gh-i04544Gh	4.55	1.09	0.74	0.35
	ImaqLY-C24-3	24	71	i44566Gh-i04593Gh	4.89	1.63	0.85	0.78
				**HSBCF_1 population**				
FB	B_1maqFB-C01-1	1	0	i33646Gh-i40884Gh	4.19	6.32	0.63	5.69
	B_1maqFB-C06-1	6	33	**i34827Gh-i15830Gh**	5.19	5.43	3.59	1.84
	B_1maqFB-C13-1	13	38	i36298Gh-i33841Gh	4.84	7.52	5.27	2.24
BN	B_1maqBN-C02-1	2	44	i02258Gh-i16398Gh	14.02	2.45	1.22	1.24
	B_1maqBN-C09-1	9	23	i03659Gh-i35107Gh	4.61	2.89	0.78	2.11
	B_1maqBN-C14-1	14	51	i25181Gh-i28149Gh	4.10	3.37	2.65	0.72
	B_1maqBN-C19-1	19	36	i43120Gh-i09946Gh	5.20	2.63	2.52	0.11
BW	B_1maqBW-C05-1	5	32	i12465Gh-i12459Gh	5.07	5.73	2.48	3.25
	B_1maqBW-C07-1	7	6	i37773Gh-i30640Gh	4.55	4.96	4.49	0.47
	B_1maqBW-C08-1	8	32	i62711Gt-i04772Gh	5.37	6.64	2.56	4.08
	B_1maqBW-C16-1	16	14	i01279Gh-i14406Gh	4.61	4.97	1.69	3.29
	B_1maqBW-C20-1	20	40	i37554Gh-i47006Gh	4.18	4.76	1.47	3.29

LP	B_1maqLP-C13-1	13	45	i13299Gh-i35111Gh	4.62	3.55	2.96	0.60
	B_1maqLP-C26-1	26	50	i16464Gh-i28856Gh	4.84	4.66	4.23	0.43
SY	B_1maqSY-C01-1	1	16	i27043Gh-i32863Gh	4.93	7.27	0.53	6.74
	B_1maqSY-C01-2	1	26	**i02994Gh-i25056Gh**	4.05	6.10	0.89	5.21
	B_1maqSY-C05-1	5	11	i45777Gh-i25259Gh	6.68	6.04	0.94	5.10
	B_1maqSY-C06-1	6	0	i06039Gh-i30129Gh	4.29	6.81	5.61	1.20
	B_1maqSY-C14-1	14	52	**i28957Gh-i36740Gh**	4.16	8.54	1.25	7.29
LY	B_1maqLY-C01-1	1	16	i27043Gh-i32863Gh	5.97	4.72	0.31	4.41
	B_1maqLY-C02-1	2	44	i02258Gh-i16398Gh	5.58	2.58	0.33	2.24
	B_1maqLY-C02-2	2	58	i39651Gh-i02432Gh	10.11	4.57	3.50	1.07
	B_1maqLY-C06-1	6	49	i37862Gh-i06396Gh	4.44	2.63	2.55	0.09
	B_1maqLY-C14-1	14	51	**i25181Gh-i28149Gh**	4.30	4.88	0.81	4.07
**MARBCF_1 population**								
FB	B_2maqFB-C03-1	3	100	i23640Gh-i34190Gh	4.84	2.07	0.50	1.57
	B_2maqFB-C09-1	9	53	i03523Gh-i15768Gh	4.61	1.70	1.52	0.18
	B_2maqFB-C17-1	17	4	i26265Gh-i03246Gh	4.13	1.30	1.14	0.16
	B_2maqFB-C19-1	19	56	i09387Gh-i10445Gh	4.99	1.71	1.69	0.02
BN	B_2maqBN-C03-1	3	103	i34190Gh-i20709Gh	5.38	10.15	1.68	8.46
	B_2maqBN-C09-1	9	53	i03523Gh-i15768Gh	5.27	7.16	4.70	2.46
	B_2maqBN-C09-2	9	59	i52208Gb-i05874Gh	6.15	3.46	1.45	2.01
	B_2maqBN-C10-1	10	14	i25267Gh-i30274Gh	6.47	3.50	2.34	1.16
	B_2maqBN-C17-1	17	19	i03593Gh-i03537Gh	4.66	2.13	1.07	1.05
	B_2maqBN-C21-1	21	7	i07262Gh-i06952Gh	5.56	1.40	0.31	1.08
	B_2maqBN-C21-2	21	50	i35971Gh-i47631Gh	5.64	7.61	3.98	3.63
BW	B_2maqBW-C09-1	9	5	i25689Gh-i17373Gh	4.25	2.23	2.12	0.11
	B_2maqBW-C11-1	11	36	i07190Gh-i43181Gh	4.20	2.20	1.35	0.85
	B_2maqBW-C17-1	17	6	i27404Gh-i03434Gh	4.31	2.44	2.16	0.28
LP	B_2maqLP-C03-1	3	78	i43226Gh-i45963Gh	4.15	3.00	2.86	0.13
	B_2maqLP-C03-2	3	99	i05394Gh-i34191Gh	4.12	2.94	2.46	0.48
	B_2maqLP-C10-1	10	4	i43940Gh-i25267Gh	4.28	3.05	2.01	1.04
	B_2maqLP-C17-1	17	16	i03341Gh-i28038Gh	4.27	2.91	2.30	0.61
SY	B_2maqSY-C09-1	9	59	i52208Gb-i05874Gh	5.07	2.55	0.29	2.26
	B_2maqSY-C11-1	11	2	**i52789Gb-i07420Gh**	5.51	4.45	0.45	4.00
	B_2maqSY-C21-1	21	19	**i06952Gh-i07714Gh**	8.47	6.91	3.65	3.26
LY	B_2maqLY-C01-1	1	39	i55243Gb-i02295Gh	4.26	3.77	1.63	2.15
	B_2maqLY-C09-1	9	59	i52208Gb-i05874Gh	5.62	5.54	0.58	4.96
	B_2maqLY-C09-1	9	70	i00393Gh-i04801Gh	4.10	7.40	1.05	6.35
	B_2maqLY-C11-1	11	2	**i52789Gb-i07420Gh**	4.60	7.99	1.09	6.90
	B_2maqLY-C21-1	21	19	**i06952Gh-i07714Gh**	10.77	19.01	9.21	9.80
	B_2maqLY-C26-1	26	14	i25834Gh-i38136Gh	4.03	9.18	5.15	4.03
	B_2maqLY-C26-2	26	38	i08565Gh-i36067Gh	4.20	9.06	4.86	4.20

[a] FB: number of fruit branches per plant; BN: number of bolls per plant; BW: boll weight; LP: lint percentage; SY: seed cotton

yield; LY: lint yield

[b] Position of QTL located on chromosome: as cM distance from the top of each chromosome

[c] Flanking markers in bold are those flanking m-QTLs identified again in e-QTLS by ICIM in additional Table S8

[d] A LOD threshold was used for declaration of QTL based on 1000 permutations at as significance level of 0.01

[e] PV: the phenotypic variance that the total additive effect explained; PV(A): the phenotypic variation that the main additive effect explained; PV(AE): the phenotypic variation that the environmental additive effect explained
