# Supplementary material for: Genetic basis of heterosis for yield and yield components explored by QTL mapping across four genetic populations in upland cotton
Source: BMC Genomics. 2018 Dec 12;19:910. doi: 10.1186/s12864-018-5289-2 (PMC6292039; doi:10.1186/s12864-018-5289-2)
Supplement: Supplementary file 8 — Table S7. Main effects and environmental interactions detected for yield and yield components in IF2MPH, HSBCF1MPH, and MARBCF1MPH datasets using the ICIM method. (PDF 261 kb) [file 12864_2018_5289_MOESM8_ESM.pdf]

Table S7 Main effects and environmental interactions detected for yield and yield components in IF$_2$MPH, HSBCF$_1$MPH, and MARBCF$_1$MPH datasets using the ICIM method

Traits[a]	m-QTL	Chr.	Position[b]	Flanking markers[c]	LOD[d]	PV[e]	PV(A)[e]	PV(AE)[e]
				**MPHIF$_2$s**				
FB	IMmaqFB-C05-1	5	64	**i37142Gh-i48326Gh**	6.04	2.76	1.85	0.92
	IMmaqFB-C06-1	6	24	i26917Gh-i06526Gh	6.12	2.63	1.67	0.96
	IMmaqFB-C09-1	9	44	i46552Gh-i24387Gh	5.03	2.19	2.02	0.17
	IMmaqFB-C09-2	9	99	i13502Gh-i25039Gh	4.52	1.83	1.42	0.42
	IMmaqFB-C18-1	18	28	i20381Gh-i63505Gm	4.62	1.90	1.53	0.38
	IMmaqFB-C19-1	19	16	i09073Gh-i50906Gb	8.90	4.27	2.82	1.45
	IMmaqFB-C19-2	19	23	i09057Gh-i31823Gh	6.25	3.09	1.07	2.02
	IMmaqFB-C22-1	22	24	i20228Gh-i12539Gh	6.76	3.27	0.58	2.69
	IMmaqFB-C26-1	26	0	i00879Gh-i08691Gh	4.98	2.39	2.03	0.36
BN	IMmaqBN-C01-1	1	11	i31992Gh-i41337Gh	5.75	1.07	0.16	0.91
	IMmaqBN-C03-1	3	2	**i03466Gh-i03416Gh**	5.59	2.40	1.81	0.59
	IMmaqBN-C04-1	4	32	i36496Gh-i46763Gh	7.73	1.63	0.59	1.04
	IMmaqBN-C09-1	9	44	**i46552Gh-i24387Gh**	4.73	1.71	0.90	0.81
	IMmaqBN-C09-2	9	47	i33836Gh-i32479Gh	4.55	1.67	1.21	0.47
	IMmaqBN-C09-3	9	90	i13502Gh-i25039Gh	5.59	1.90	0.33	1.57
	IMmaqBN-C13-1	13	14	i30934Gh-i18151Gh	6.46	1.42	1.32	0.10
	IMmaqBN-C13-2	13	44	i49771Gh-i49487Gh	4.47	1.40	1.07	0.33
	IMmaqBN-C17-1	17	2	i03339Gh-i03336Gh	5.07	2.27	1.58	0.70
	IMmaqBN-C18-1	18	14	**i13145Gh-i29829Gh**	5.59	2.77	1.07	1.71
	IMmaqBN-C19-1	19	23	i09057Gh-i31823Gh	4.66	0.99	0.07	0.92
	IMmaqBN-C19-2	19	27	i09636Gh-i37157Gh	5.07	1.74	1.28	0.45
BW	IMmaqBW-C01-1	1	18	i30221Gh-i37135Gh	4.96	2.92	1.71	1.21
	IMmaqBW-C03-1	3	93	i31859Gh-i42939Gh	4.42	1.41	0.02	1.39
	IMmaqBW-C08-1	8	20	i25482Gh-i25868Gh	5.11	1.59	0.83	0.76
	IMmaqBW-C08-2	8	24	i26219Gh-i32773Gh	7.25	2.41	2.02	0.39
	IMmaqBW-C12-1	12	52	i25940Gh-i46260Gh	4.51	1.39	0.33	1.06
	IMmaqBW-C14-1	14	10	i46775Gh-i43468Gh	5.58	2.68	0.65	2.03
	IMmaqBW-C18-1	18	84	i25079Gh-i37364Gh	4.73	1.86	0.63	1.23
LP	IMmaqLP-C10-1	10	13	i43940Gh-i25267Gh	7.50	10.69	3.91	6.78
	IMmaqLP-C16-1	16	24	i01567Gh-i29688Gh	4.42	2.70	0.66	2.04
	IMmaqLP-C18-1	18	11	i13145Gh-i29829Gh	5.48	5.11	1.19	3.92
	IMmaqLP-C18-2	18	55	i13319Gh-i42821Gh	5.42	6.74	2.65	4.09
	IMmaqLP-C20-1	20	6	**i46485Gh-i11727Gh**	4.90	1.96	0.07	1.88
SY	IMmaqSY-C02-1	2	81	i38985Gh-i30800Gh	5.10	1.89	1.60	0.29
	IMmaqSY-C13-1	13	44	i49771Gh-i49487Gh	6.32	2.41	2.26	0.15
	IMmaqSY-C13-2	13	48	i49618Gh-i20979Gh	4.60	1.71	1.70	0.01
	IMmaqSY-C13-3	13	56	i46408Gh-i12964Gh	5.08	2.14	1.88	0.26
	IMmaqSY-C15-1	15	26	i25137Gh-i23643Gh	4.94	2.49	1.40	1.09

	IMmaqSY-C16-1	16	16	i01766Gh-i00144Gh	5.26	2.41	1.60	0.81
	IMmaqSY-C18-1	18	35	i32883Gh-i13851Gh	4.97	2.17	1.53	0.65
LY	IMmaqLY-C02-1	2	81	i38985Gh-i30800Gh	5.09	1.50	1.26	0.23
	IMmaqLY-C13-1	13	0	**i17985Gh-i12984Gh**	4.56	1.48	0.64	0.84
	IMmaqLY-C13-2	13	24	i13079Gh-i36296Gh	4.46	1.53	1.44	0.09
	IMmaqLY-C13-3	13	44	i49771Gh-i49487Gh	5.02	1.56	1.47	0.09
	IMmaqLY-C13-4	13	56	i46408Gh-i12964Gh	5.18	1.72	1.50	0.22
	IMmaqLY-C15-1	15	26	i25137Gh-i23643Gh	4.69	1.95	1.13	0.82
	IMmaqLY-C16-1	16	16	i01766Gh-i00144Gh	5.20	1.85	1.42	0.43
	IMmaqLY-C18-1	18	35	i32883Gh-i13851Gh	4.47	1.46	1.12	0.35
	IMmaqLY-C18-2	18	106	i45991Gh-i13081Gh	5.16	1.59	1.53	0.05
	IMmaqLY-C21-1	21	24	i22367Gh-i47711Gh	4.78	1.66	1.08	0.58
**HSBCF_1MPHs**								
FB	B_1MmaqFB-C07-1	7	30	i38586Gh-i26820Gh	5.02	4.68	3.61	1.08
	B_1MmaqFB-C16-1	16	34	i42833Gh-i36412Gh	4.03	3.85	2.54	1.31
	B_1MmaqFB-C22-1	22	34	i12408Gh-i12538Gh	4.71	4.14	2.54	1.60
BN	B_1MmaqBN-C01-1	1	29	i23213Gh-i24266Gh	4.23	1.02	0.75	0.27
	B_1MmaqBN-C03-1	3	80	i43226Gh-i45963Gh	4.17	1.66	1.21	0.45
	B_1MmaqBN-C10-1	10	4	i43940Gh-i25267Gh	6.25	2.43	2.03	0.40
	B_1MmaqBN-C14-1	14	42	i34003Gh-i05404Gh	4.03	1.95	1.80	0.15
	B_1MmaqBN-C14-1	14	50	i34963Gh-i44045Gh	5.55	2.90	2.65	0.24
	B_1MmaqBN-C20-1	20	0	i17414Gh-i17417Gh	4.92	3.27	2.17	1.10
	B_1MmaqBN-C21-1	21	57	**i41432Gh-i07219Gh**	4.32	1.60	0.06	1.54
LP	B_1MmaqLP-C03-1	3	80	i43226Gh-i45963Gh	4.47	3.92	3.74	0.18
	B_1MmaqLP-C05-1	5	53	**i64389Gm-i48057Gh**	4.50	4.41	2.92	1.49
	B_1MmaqLP-C10-1	10	7	i43940Gh-i25267Gh	5.83	4.39	3.23	1.16
SY	B_1MmaqSY-C01-1	1	29	i23213Gh-i24266Gh	5.06	3.00	1.80	1.21
	B_1MmaqSY-C05-1	5	11	i45777Gh-i25259Gh	8.98	7.54	1.79	5.76
	B_1MmaqSY-C13-1	13	33	i36415Gh-i62433Gt	4.93	5.61	0.20	5.42
	B_1MmaqSY-C13-2	13	40	i46668Gh-i00187Gh	4.36	3.49	2.54	0.94
	B_1MmaqSY-C14-1	14	52	**i28957Gh-i36740Gh**	6.83	15.93	8.86	7.07
	B_1MmaqSY-C18-1	18	10	i13145Gh-i29829Gh	4.08	7.74	3.62	4.12
LY	B_1MmaqLY-C13-1	13	33	i36415Gh-i62433Gt	4.24	4.42	0.21	4.21
	B_1MmaqLY-C14-1	14	52	**i28957Gh-i36740Gh**	5.67	11.88	6.89	4.98
**MARBCF_1MPHs**								
FB	B_2MmaqFB-C07-1	7	19	i01765Gh-i30139Gh	6.25	5.78	1.50	4.28
	B_2MmaqFB-C07-2	7	31	i26820Gh-i66007Ga	4.85	4.27	2.49	1.78
	B_2MmaqFB-C19-1	19	46	i21172Gh-i09629Gh	4.76	6.07	5.26	0.81
BN	B_2MmaqBN-C03-1	3	101	i34190Gh-i20709Gh	4.91	8.11	2.67	5.44
	B_2MmaqBN-C21-1	21	49	i31481Gh-i34296Gh	6.94	13.35	3.61	9.74
BW	B_2MmaqBW-C02-1	2	68	i02544Gh-i00890Gh	4.33	4.74	1.81	2.94
	B_2MmaqBW-C03-1	3	81	i45963Gh-i21218Gh	4.80	6.54	3.96	2.57

	B_2MmaqBW-C03-2	3	98	i42939Gh-i05394Gh	5.15	7.15	3.98	3.17
LP	B_2MmaqLP-C03-1	3	98	i42939Gh-i05394Gh	5.30	6.05	3.61	2.44
	B_2MmaqLP-C07-1	7	30	i38586Gh-i26820Gh	5.29	6.03	3.56	2.48
SY	B_2MmaqSY-C08-1	8	17	i00540Gh-i25482Gh	4.49	7.18	3.95	3.23
	B_2MmaqSY-C16-1	16	22	**i46747Gh-i01444Gh**	5.21	3.65	2.18	1.47
	B_2MmaqSY-C21-1	21	19	**i06952Gh-i07714Gh**	4.18	5.04	2.89	2.15
LY	B_2MmaqLY-C08-1	8	15	i04570Gh-i04506Gh	4.26	3.36	2.65	0.71
	B_2MmaqLY-C21-1	21	19	i06952Gh-i07714Gh	4.83	2.98	1.52	1.47

[a] FB: number of fruit branches per plant; BN: number of bolls per plant; BW: boll weight; LP: lint percentage; SY: seed cotton yield; LY: lint yield

[b] Position of QTL located on chromosome: as cM distance from the top of each chromosome

[c] Flanking markers in bold are those flanking m-QTLs identified again in e-QTLS by ICIM in additional Table S9

[d] A LOD threshold was used for declaration of QTL based on 1000 permutations at as significance level of 0.01

[e] PV: the phenotypic variance that the total additive and dominance effects explained; PV (A): phenotypic variance explained by the main additive and dominance effects; PV (AE): phenotypic variance explained by the environmental additive and dominance effects
