# Supplementary material for: Genetic basis of heterosis for yield and yield components explored by QTL mapping across four genetic populations in upland cotton
Source: BMC Genomics. 2018 Dec 12;19:910. doi: 10.1186/s12864-018-5289-2 (PMC6292039; doi:10.1186/s12864-018-5289-2)
Supplement: Supplementary file 9 — Table S8. Epistatic effects and environmental interactions detected for yield and yield components in RIL, IF2 and two BCF1 datasets using the ICIM method. (PDF 648 kb) [file 12864_2018_5289_MOESM9_ESM.pdf]

Table S8 Epistatic effects and environmental interactions detected for yield and yield components in RIL, IF_2 and two BCF_1 populations using the ICIM method

Traits[a]	e-QTL	Type of epistasis[b]	Chr.i[c]	Position1[d]	Flanking markers 1[e]	Chr.j[c]	Position2[d]	Flanking markers 2[e]	LOD[f]	PV[g]	PV(AA)[g]	PV(AAE)[g]
						RIL population						
FB	Rmeq-FB-1	III	5	35	i16543Gh-i22374Gh	13	50	i46212Gh-i13740Gh	7.42	3.26	1.70	1.56
	Rmeq-FB-2	III	15	0	i02955Gh-i02314Gh	20	5	i00478Gh-i11539Gh	8.78	4.76	4.51	0.26
	Rmeq-FB-3	III	4	0	i50068Gb-i26515Gh	24	10	i04567Gh-i15176Gh	6.09	3.21	2.91	0.31
	Rmeq-FB-4	III	22	35	i12538Gh-i12818Gh	24	30	i15200Gh-i04548Gh	7.25	3.68	1.30	2.38
BN	Rmeq-BN-1	III	4	0	i50068Gb-i26515Gh	9	25	i05664Gh-i08801Gh	6.95	2.31	2.18	0.13
	Rmeq-BN-2	III	3	115	i05514Gh-i29314Gh	9	60	i05874Gh-i05194Gh	7.69	3.45	1.40	2.05
	Rmeq-BN-3	III	3	70	i22169Gh-i33635Gh	10	55	i35169Gh-i11724Gh	9.13	4.46	2.79	1.67
	Rmeq-BN-4	III	3	85	i31859Gh-i42939Gh	11	35	i40251Gh-i07190Gh	7.00	4.11	0.95	3.16
	Rmeq-BN-5	III	2	55	i02246Gh-i00463Gh	13	30	i24929Gh-i13848Gh	6.66	3.12	1.81	1.30
	Rmeq-BN-6	III	1	35	i02298Gh-i42430Gh	14	5	i04862Gh-i15284Gh	6.97	3.89	2.86	1.03
	Rmeq-BN-7	III	3	5	i34758Gh-i49377Gh	14	5	i04862Gh-i15284Gh	6.91	3.10	2.34	0.76
	Rmeq-BN-8	III	6	35	i06036Gh-i06037Gh	14	5	i04862Gh-i15284Gh	7.27	1.76	1.75	0.01
	Rmeq-BN-9	III	7	30	i38586Gh-i26820Gh	14	5	i04862Gh-i15284Gh	7.33	3.91	2.90	1.01
	Rmeq-BN-10	III	10	45	i26780Gh-i33011Gh	14	35	i04933Gh-i43400Gh	7.88	3.75	2.72	1.03
	Rmeq-BN-11	III	15	40	i02315Gh-i07215Gh	16	15	i14406Gh-i01766Gh	6.77	3.49	2.54	0.95
	Rmeq-BN-12	III	1	30	i23213Gh-i24266Gh	18	15	i31442Gh-i13146Gh	7.89	4.05	3.06	0.99
	Rmeq-BN-13	III	3	115	i05514Gh-i29314Gh	20	20	i26356Gh-i11706Gh	8.50	4.30	2.92	1.38
	Rmeq-BN-14	III	18	25	i13146Gh-i20346Gh	20	40	i37554Gh-i47006Gh	8.13	3.99	2.10	1.89
	Rmeq-BN-15	III	1	35	i02298Gh-i42430Gh	21	5	i07547Gh-i20988Gh	6.80	3.78	2.78	1.00
	Rmeq-BN-16	III	3	5	i34758Gh-i49377Gh	21	5	i07547Gh-i20988Gh	6.77	2.99	2.25	0.74
	Rmeq-BN-17	III	6	35	i06036Gh-i06037Gh	21	5	i07547Gh-i20988Gh	7.46	1.71	1.69	0.03
	Rmeq-BN-18	III	7	30	i38586Gh-i26820Gh	21	5	i07547Gh-i20988Gh	7.31	3.80	2.81	0.99
	Rmeq-BN-19	III	11	10	i47563Gh-i01036Gh	21	5	i07547Gh-i20988Gh	6.64	2.13	1.73	0.40

	Rmeq-BN-20	III	16	30	i22650Gh-i27519Gh	21	5	i07547Gh-i20988Gh	6.66	1.65	1.65	0.00
	Rmeq-BN-21	III	14	5	i04862Gh-i15284Gh	22	45	i44682Gh-i25111Gh	6.88	3.47	2.59	0.87
	Rmeq-BN-22	III	21	5	i07547Gh-i20988Gh	22	45	i44682Gh-i25111Gh	6.73	3.37	2.52	0.85
	Rmeq-BN-23	III	22	35	i12538Gh-i12818Gh	24	30	i15200Gh-i04548Gh	7.31	3.94	2.20	1.75
	Rmeq-BN-24	III	3	100	i23640Gh-i34190Gh	24	40	i48423Gh-i43942Gh	7.13	3.75	2.83	0.93
	Rmeq-BN-25	III	14	5	i04862Gh-i15284Gh	25	35	i11465Gh-i39910Gh	6.85	3.68	2.70	0.98
	Rmeq-BN-26	III	21	5	i07547Gh-i20988Gh	25	35	i11465Gh-i39910Gh	6.77	3.58	2.62	0.96
	Rmeq-BN-27	III	18	75	i13532Gh-i43889Gh	26	40	i36067Gh-i08578Gh	8.87	4.24	2.69	1.55
BW	Rmeq-BW-1	III	6	25	i26917Gh-i06526Gh	11	35	i40251Gh-i07190Gh	6.14	2.93	2.65	0.29
	Rmeq-BW-2	III	13	60	i12964Gh-i29310Gh	16	45	i54957Gb-i59324Gb	6.26	3.11	2.37	0.74
	Rmeq-BW-3	III	13	10	i45163Gh-i30934Gh	18	95	i45991Gh-i13081Gh	6.06	3.01	2.56	0.45
	Rmeq-BW-4	III	8	50	i01126Gh-i04719Gh	20	80	i11915Gh-i11478Gh	6.29	3.37	2.93	0.44
LP	Rmeq-LP-1	III	7	55	i01629Gh-i14398Gh	11	15	i01036Gh-i07468Gh	6.23	2.91	1.40	1.51
	Rmeq-LP-2	III	11	15	i01036Gh-i07468Gh	16	70	i54704Gb-i01693Gh	7.41	3.71	1.43	2.28
	Rmeq-LP-3	II	9	55	i29408Gh-i03595Gh	18	5	**i13754Gh-i13145Gh**	6.05	2.07	1.15	0.91
	Rmeq-LP-4	III	18	35	i32883Gh-i13851Gh	18	50	i13451Gh-i38577Gh	6.13	2.96	1.72	1.23
	Rmeq-LP-5	III	17	55	i03522Gh-i03688Gh	18	110	i45991Gh-i13081Gh	8.56	3.43	2.36	1.07
	Rmeq-LP-6	III	11	40	i43181Gh-i16165Gh	22	40	i12810Gh-i17697Gh	6.04	2.64	1.59	1.04
	Rmeq-LP-7	III	13	15	i30934Gh-i18151Gh	25	50	i40453Gh-i46187Gh	6.91	2.70	1.87	0.83
	Rmeq-LP-8	II	4	15	**i41085Gh-i38159Gh**	26	20	i37251Gh-i23249Gh	6.85	3.28	1.61	1.66
	Rmeq-LP-9	II	12	0	**i40974Gh-i48211Gh**	26	20	i37251Gh-i23249Gh	6.40	3.34	1.38	1.96
	Rmeq-LP-10	III	16	25	i29957Gh-i01669Gh	26	25	i08562Gh-i49188Gh	7.24	2.93	1.83	1.10
SY	Rmeq-SY-1	III	9	0	i40221Gh-i15598Gh	9	10	i05758Gh-i19700Gh	7.55	3.28	1.68	1.60
	Rmeq-SY-2	III	1	0	i33646Gh-i40884Gh	15	5	i02306Gh-i02317Gh	6.57	4.05	2.74	1.31
	Rmeq-SY-3	III	4	30	i10502Gh-i36496Gh	15	5	i02306Gh-i02317Gh	6.11	3.12	2.58	0.53
	Rmeq-SY-4	III	6	10	i06061Gh-i05824Gh	15	35	i17844Gh-i37620Gh	6.34	3.12	2.36	0.76

	Rmeq-SY-5	III	11	10	i47563Gh-i01036Gh	18	80	i43889Gh-i25079Gh	6.54	2.99	2.11	0.88
	Rmeq-SY-6	III	5	40	i08988Gh-i45534Gh	19	40	i08786Gh-i00558Gh	7.04	3.90	2.82	1.08
	Rmeq-SY-7	III	3	70	i22169Gh-i33635Gh	21	60	i22642Gh-i41613Gh	6.14	3.51	3.41	0.10
	Rmeq-SY-8	III	4	20	i00135Gh-i47058Gh	22	20	i12927Gh-i12929Gh	6.60	3.62	3.13	0.49
	Rmeq-SY-9	III	8	25	i26219Gh-i32773Gh	22	50	i44682Gh-i25111Gh	6.79	3.83	3.43	0.40
	Rmeq-SY-10	III	18	110	i45991Gh-i13081Gh	25	45	i21894Gh-i19984Gh	9.80	5.11	2.89	2.22
	Rmeq-SY-11	III	20	80	i11915Gh-i11478Gh	25	55	i11287Gh-i17145Gh	7.06	3.90	3.24	0.65
	Rmeq-SY-12	III	2	75	i02276Gh-i43470Gh	26	5	i08062Gh-i33827Gh	6.27	3.24	1.55	1.69
LY	Rmeq-LY-1	III	9	0	i40221Gh-i15598Gh	9	10	i05758Gh-i19700Gh	6.54	2.64	1.35	1.29
	Rmeq-LY-2	III	6	45	i23722Gh-i37862Gh	14	45	i27231Gh-i36385Gh	6.06	3.14	2.89	0.25
	Rmeq-LY-3	III	1	0	i33646Gh-i40884Gh	15	5	i02306Gh-i02317Gh	7.15	4.24	2.94	1.30
	Rmeq-LY-4	III	13	25	i13079Gh-i36296Gh	15	5	i02306Gh-i02317Gh	6.27	3.11	2.81	0.29
	Rmeq-LY-5	III	13	25	i13079Gh-i36296Gh	18	110	i45991Gh-i13081Gh	7.23	1.47	1.47	0.00
	Rmeq-LY-6	III	14	55	i23629Gh-i15587Gh	18	110	i45991Gh-i13081Gh	7.40	3.99	3.19	0.79
	Rmeq-LY-7	III	5	40	i08988Gh-i45534Gh	19	40	i08786Gh-i00558Gh	6.06	3.30	2.24	1.05
	Rmeq-LY-8	III	13	25	i13079Gh-i36296Gh	20	0	i17414Gh-i17417Gh	6.17	2.69	1.90	0.79
	Rmeq-LY-9	III	3	65	i49177Gh-i39896Gh	20	25	i25398Gh-i11734Gh	6.98	3.91	3.54	0.37
	Rmeq-LY-10	III	3	70	i22169Gh-i33635Gh	21	60	i22642Gh-i41613Gh	7.40	4.12	4.09	0.03
	Rmeq-LY-11	III	4	20	i00135Gh-i47058Gh	22	20	i12927Gh-i12929Gh	6.20	3.35	3.02	0.33
	Rmeq-LY-12	III	18	110	i45991Gh-i13081Gh	25	45	i21894Gh-i19984Gh	10.90	5.64	3.61	2.03
	Rmeq-LY-13	III	20	5	i00478Gh-i11539Gh	25	55	i11287Gh-i17145Gh	8.17	4.31	4.24	0.07
	Rmeq-LY-14	III	2	75	i02276Gh-i43470Gh	26	5	i08062Gh-i33827Gh	6.33	3.25	1.82	1.43
	Rmeq-LY-15	III	10	0	i43940Gh-i25267Gh	26	15	i25512Gh-i07941Gh	6.99	3.93	2.11	1.82
**IF_2 population**												
FB	Imeq-FB-1	III	18	0	i13766Gh-i13754Gh	18	45	i39369Gh-i13456Gh	8.97	3.52	1.93	1.59
	Imeq-FB-2	III	18	35	i32883Gh-i13851Gh	24	55	i14999Gh-i14993Gh	8.73	2.76	1.35	1.42

BN	Imeq-BN-1	III	3	75	i43226Gh-i45963Gh	3	85	i31859Gh-i42939Gh	12.02	0.80	0.80	0.00
	Imeq-BN-2	III	5	40	i08988Gh-i45534Gh	6	40	i06505Gh-i23722Gh	8.76	0.32	0.08	0.24
	Imeq-BN-3	III	1	35	i02298Gh-i42430Gh	7	60	i14398Gh-i01824Gh	11.17	2.75	2.23	0.52
	Imeq-BN-4	III	2	20	i17680Gh-i02755Gh	7	60	i14398Gh-i01824Gh	10.79	1.86	1.50	0.36
	Imeq-BN-5	III	2	95	i07717Gh-i09654Gh	8	25	i26219Gh-i32773Gh	9.34	3.30	2.46	0.84
	Imeq-BN-6	III	4	15	i41085Gh-i38159Gh	9	45	i46552Gh-i24387Gh	10.64	3.93	0.78	3.15
	Imeq-BN-7	III	9	10	i05758Gh-i19700Gh	9	70	i00393Gh-i04801Gh	9.21	3.47	2.54	0.93
	Imeq-BN-8	III	5	35	i16543Gh-i22374Gh	10	50	i33011Gh-i22938Gh	8.79	1.01	0.83	0.18
	Imeq-BN-9	III	1	20	i47599Gh-i49208Gh	11	40	i43181Gh-i16165Gh	9.08	2.05	1.81	0.24
	Imeq-BN-10	III	10	50	i33011Gh-i22938Gh	14	55	i23629Gh-i15587Gh	9.01	1.59	1.48	0.11
	Imeq-BN-11	III	9	70	i00393Gh-i04801Gh	14	85	i05035Gh-i22015Gh	12.14	4.10	2.93	1.18
	Imeq-BN-12	III	4	0	i50068Gb-i26515Gh	15	0	i02955Gh-i02314Gh	10.37	3.49	2.57	0.92
	Imeq-BN-13	III	9	70	i00393Gh-i04801Gh	20	20	i26356Gh-i11706Gh	8.67	3.23	2.64	0.59
	Imeq-BN-14	III	21	50	i35971Gh-i47631Gh	21	60	i22642Gh-i41613Gh	9.92	1.09	1.09	0.00
	Imeq-BN-15	III	14	15	i15343Gh-i31037Gh	23	0	i06287Gh-i06171Gh	8.83	2.46	1.54	0.92
	Imeq-BN-16	III	21	30	i47711Gh-i07558Gh	24	30	i15200Gh-i04548Gh	10.14	3.06	2.57	0.49
	Imeq-BN-17	III	3	65	i49177Gh-i39896Gh	25	20	i41210Gh-i42629Gh	9.31	2.91	1.64	1.27
	Imeq-BN-18	III	10	50	i33011Gh-i22938Gh	25	50	i40453Gh-i46187Gh	9.45	1.88	0.20	1.68
	Imeq-BN-19	III	20	5	i00478Gh-i11539Gh	25	50	i40453Gh-i46187Gh	9.79	3.77	1.29	2.48
	Imeq-BN-20	III	9	70	i00393Gh-i04801Gh	26	30	i28715Gh-i21900Gh	9.99	2.99	2.65	0.34
	Imeq-BN-21	III	3	70	i22169Gh-i33635Gh	26	45	i22171Gh-i00945Gh	10.85	2.99	2.55	0.45
	Imeq-BN-22	III	7	55	i01629Gh-i14398Gh	26	45	i22171Gh-i00945Gh	10.36	3.23	2.65	0.58
	Imeq-BN-23	III	8	10	i24295Gh-i00234Gh	26	45	i22171Gh-i00945Gh	8.89	2.74	2.14	0.60
	Imeq-BN-24	III	10	55	i35169Gh-i11724Gh	26	45	i22171Gh-i00945Gh	9.25	2.25	1.98	0.27
	Imeq-BN-25	III	11	0	i52789Gb-i07420Gh	26	45	i22171Gh-i00945Gh	9.99	3.43	2.91	0.52
	Imeq-BN-26	III	13	40	i46668Gh-i00187Gh	26	45	i22171Gh-i00945Gh	8.51	3.02	2.34	0.68

	Imeq-BN-27	III	16	55	i21384Gh-i44137Gh	26	45	i22171Gh-i00945Gh	9.30	3.32	2.64	0.69
	Imeq-BN-28	III	19	50	i08832Gh-i09452Gh	26	45	i22171Gh-i00945Gh	9.24	2.80	2.54	0.26
	Imeq-BN-29	III	26	15	i25512Gh-i07941Gh	26	45	i22171Gh-i00945Gh	13.56	4.16	3.38	0.78
BW	Imeq-BW-1	III	8	50	i01126Gh-i04719Gh	14	10	i46775Gh-i43468Gh	8.13	2.81	1.78	1.03
LP	Imeq-LP-1	III	7	10	i24917Gh-i26814Gh	9	5	i25689Gh-i17373Gh	8.80	2.49	0.72	1.76
	Imeq-LP-2	III	3	115	i05514Gh-i29314Gh	17	25	i03512Gh-i22912Gh	9.41	1.56	1.26	0.31
	Imeq-LP-3	III	5	20	i35761Gh-i09052Gh	17	25	i03512Gh-i22912Gh	12.39	3.23	1.41	1.82
	Imeq-LP-4	III	6	40	i06505Gh-i23722Gh	17	25	i03512Gh-i22912Gh	9.72	1.88	1.14	0.74
	Imeq-LP-5	III	8	30	i54149Gb-i00217Gh	17	25	i03512Gh-i22912Gh	10.95	1.71	1.52	0.19
	Imeq-LP-6	III	12	0	i40974Gh-i48211Gh	17	25	i03512Gh-i22912Gh	9.90	2.99	1.97	1.01
	Imeq-LP-7	III	14	25	i15375Gh-i05040Gh	17	25	i03512Gh-i22912Gh	9.32	2.90	1.05	1.85
	Imeq-LP-8	III	14	85	i05035Gh-i22015Gh	18	15	i31442Gh-i13146Gh	9.21	2.02	0.72	1.30
	Imeq-LP-9	III	5	55	i09147Gh-i42098Gh	18	30	i32883Gh-i13851Gh	8.71	2.35	1.53	0.82
	Imeq-LP-10	III	2	75	i02276Gh-i43470Gh	18	40	i26970Gh-i39369Gh	8.59	2.82	0.89	1.93
	Imeq-LP-11	III	2	95	i07717Gh-i09654Gh	20	25	i25398Gh-i11734Gh	8.72	3.51	1.77	1.75
	Imeq-LP-12	III	4	0	i50068Gb-i26515Gh	21	70	i33529Gh-i43642Gh	10.17	2.58	1.40	1.18
	Imeq-LP-13	III	5	20	i35761Gh-i09052Gh	21	70	i33529Gh-i43642Gh	9.51	1.85	0.88	0.98
	Imeq-LP-14	III	6	40	i06505Gh-i23722Gh	21	70	i33529Gh-i43642Gh	10.49	1.55	0.58	0.96
	Imeq-LP-15	III	7	5	i37773Gh-i30640Gh	21	70	i33529Gh-i43642Gh	9.76	1.62	0.84	0.78
	Imeq-LP-16	III	2	95	i07717Gh-i09654Gh	22	20	i12927Gh-i12929Gh	8.77	2.51	1.29	1.22
	Imeq-LP-17	III	20	80	i11915Gh-i11478Gh	24	55	i14999Gh-i14993Gh	9.61	2.90	1.86	1.05
	Imeq-LP-18	II	17	25	i03512Gh-i22912Gh	25	25	**i27022Gh-i11449Gh**	9.51	2.15	1.26	0.89
	Imeq-LP-19	III	2	20	i17680Gh-i02755Gh	25	35	i11465Gh-i39910Gh	9.70	1.12	0.54	0.58
	Imeq-LP-20	III	5	55	i09147Gh-i42098Gh	25	35	i11465Gh-i39910Gh	8.59	1.27	0.57	0.69
	Imeq-LP-21	III	18	15	i31442Gh-i13146Gh	25	35	i11465Gh-i39910Gh	8.64	1.20	0.68	0.51
	Imeq-LP-22	III	21	70	i33529Gh-i43642Gh	25	45	i21894Gh-i19984Gh	9.96	1.10	0.64	0.46

	Imeq-LP-23	III	3	60	i35903Gh-i20966Gh	25	50	i40453Gh-i46187Gh	9.42	2.72	1.87	0.85
	Imeq-LP-24	III	21	50	i35971Gh-i47631Gh	26	5	i08062Gh-i33827Gh	9.51	3.45	1.14	2.31
	Imeq-LP-25	III	5	20	i35761Gh-i09052Gh	26	20	i37251Gh-i23249Gh	10.70	3.35	1.36	1.98
	Imeq-LP-26	III	20	25	i25398Gh-i11734Gh	26	20	i37251Gh-i23249Gh	9.44	3.47	1.37	2.10
SY	Imeq-SY-1	III	1	10	i53010Gb-i21390Gh	3	40	i00971Gh-i46613Gh	8.75	2.13	1.89	0.24
	Imeq-SY-2	III	2	60	i02432Gh-i14623Gh	3	40	i00971Gh-i46613Gh	9.83	1.93	1.76	0.17
	Imeq-SY-3	III	3	70	i22169Gh-i33635Gh	4	15	i41085Gh-i38159Gh	11.31	3.36	1.94	1.43
	Imeq-SY-4	III	4	15	i41085Gh-i38159Gh	5	45	i29825Gh-i01144Gh	8.88	3.04	1.82	1.22
	Imeq-SY-5	III	5	40	i08988Gh-i45534Gh	6	40	i06505Gh-i23722Gh	10.66	1.38	0.92	0.45
	Imeq-SY-6	III	3	40	i00971Gh-i46613Gh	7	30	i38586Gh-i26820Gh	10.75	0.93	0.65	0.28
	Imeq-SY-7	II	7	10	i24917Gh-i26814Gh	7	40	**i34772Gh-i43291Gh**	8.71	2.51	1.71	0.80
	Imeq-SY-8	III	2	20	i17680Gh-i02755Gh	7	60	i14398Gh-i01824Gh	10.55	1.48	1.48	0.00
	Imeq-SY-9	III	2	95	i07717Gh-i09654Gh	8	5	i31145Gh-i40270Gh	9.23	3.63	2.40	1.23
	Imeq-SY-10	III	1	5	i21210Gh-i35065Gh	8	50	i01126Gh-i04719Gh	9.50	3.51	2.31	1.20
	Imeq-SY-11	III	8	50	i01126Gh-i04719Gh	9	15	i40336Gh-i07864Gh	10.13	3.34	2.59	0.75
	Imeq-SY-12	III	5	65	i37142Gh-i48326Gh	9	40	i10438Gh-i08573Gh	9.45	3.06	1.10	1.97
	Imeq-SY-13	III	9	10	i05758Gh-i19700Gh	10	50	i33011Gh-i22938Gh	8.65	1.97	1.65	0.31
	Imeq-SY-14	III	4	25	i10502Gh-i36496Gh	11	0	i52789Gb-i07420Gh	9.33	3.46	1.81	1.65
	Imeq-SY-15	III	5	45	i29825Gh-i01144Gh	11	40	i43181Gh-i16165Gh	8.74	2.73	2.02	0.71
	Imeq-SY-16	III	9	70	i00393Gh-i04801Gh	13	10	i45163Gh-i30934Gh	11.23	3.92	3.24	0.68
	Imeq-SY-17	III	3	40	i00971Gh-i46613Gh	17	10	i44474Gh-i03341Gh	9.15	0.36	0.23	0.13
	Imeq-SY-18	III	15	0	i02955Gh-i02314Gh	17	50	i14844Gh-i03522Gh	9.32	2.81	2.35	0.46
	Imeq-SY-19	II	3	5	**i34758Gh-i49377Gh**	18	0	i13766Gh-i13754Gh	8.52	4.35	1.92	2.43
	Imeq-SY-20	III	7	20	i01696Gh-i57601Gb	18	0	i13766Gh-i13754Gh	8.82	3.30	2.06	1.24
	Imeq-SY-21	III	8	10	i24295Gh-i00234Gh	18	0	i13766Gh-i13754Gh	8.72	2.91	1.58	1.33
	Imeq-SY-22	III	14	25	i15375Gh-i05040Gh	20	5	i00478Gh-i11539Gh	8.89	2.56	1.72	0.84

	Imeq-SY-23	III	7	20	i01696Gh-i57601Gb	20	10	i39228Gh-i34769Gh	11.92	4.09	2.93	1.16
	Imeq-SY-24	III	8	55	i01126Gh-i04719Gh	21	45	i07446Gh-i16079Gh	9.12	0.33	0.05	0.28
	Imeq-SY-25	III	3	80	i43226Gh-i45963Gh	21	60	i22642Gh-i41613Gh	8.74	2.53	2.10	0.43
	Imeq-SY-26	III	21	50	i35971Gh-i47631Gh	21	60	i22642Gh-i41613Gh	11.78	1.46	1.46	0.00
	Imeq-SY-27	III	5	65	i37142Gh-i48326Gh	22	50	i44682Gh-i25111Gh	10.78	3.11	2.22	0.89
	Imeq-SY-28	III	4	10	i31054Gh-i41405Gh	24	45	i26213Gh-i00339Gh	9.16	2.30	1.64	0.65
	Imeq-SY-29	III	3	40	i00971Gh-i46613Gh	25	20	i41210Gh-i42629Gh	12.13	2.24	1.78	0.46
	Imeq-SY-30	III	12	15	i40974Gh-i48211Gh	25	20	i41210Gh-i42629Gh	8.90	3.10	1.65	1.45
	Imeq-SY-31	III	19	20	i08987Gh-i09220Gh	25	20	i41210Gh-i42629Gh	9.58	3.23	2.04	1.19
	Imeq-SY-32	II	3	5	**i34758Gh-i49377Gh**	26	0	i00879Gh-i08691Gh	8.69	2.33	1.45	0.88
	Imeq-SY-33	III	7	65	i14398Gh-i01824Gh	26	20	i37251Gh-i23249Gh	11.29	3.50	1.99	1.51
	Imeq-SY-34	III	21	60	i22642Gh-i41613Gh	26	45	i22171Gh-i00945Gh	9.31	3.29	1.72	1.57
LY	Imeq-LY-1	III	3	75	i43226Gh-i45963Gh	3	85	i31859Gh-i42939Gh	10.04	0.59	0.59	0.00
	Imeq-LY-2	III	3	70	i22169Gh-i33635Gh	4	15	i41085Gh-i38159Gh	9.94	2.68	1.37	1.32
	Imeq-LY-3	III	3	90	i31859Gh-i42939Gh	5	40	i08988Gh-i45534Gh	11.93	0.24	0.05	0.20
	Imeq-LY-4	III	1	10	i53010Gb-i21390Gh	6	40	i06505Gh-i23722Gh	9.43	3.24	2.63	0.61
	Imeq-LY-5	III	2	20	i17680Gh-i02755Gh	7	60	i14398Gh-i01824Gh	9.35	1.63	1.63	0.00
	Imeq-LY-6	III	2	95	i07717Gh-i09654Gh	8	5	i31145Gh-i40270Gh	10.35	3.76	2.34	1.41
	Imeq-LY-7	III	3	65	i49177Gh-i39896Gh	8	25	i26219Gh-i32773Gh	8.61	2.86	1.02	1.84
	Imeq-LY-8	III	6	55	i06396Gh-i06056Gh	8	30	i54149Gb-i00217Gh	9.00	2.62	1.50	1.12
	Imeq-LY-9	III	8	10	i24295Gh-i00234Gh	8	30	i54149Gb-i00217Gh	8.53	2.58	1.25	1.33
	Imeq-LY-10	III	1	5	i21210Gh-i35065Gh	8	50	i01126Gh-i04719Gh	9.49	3.62	2.29	1.34
	Imeq-LY-11	III	8	50	i01126Gh-i04719Gh	9	15	i40336Gh-i07864Gh	8.68	3.03	2.31	0.71
	Imeq-LY-12	III	5	65	i37142Gh-i48326Gh	9	40	i10438Gh-i08573Gh	9.24	3.22	1.00	2.22
	Imeq-LY-13	III	7	60	i14398Gh-i01824Gh	11	40	i43181Gh-i16165Gh	8.51	2.54	1.94	0.60
	Imeq-LY-14	III	8	30	i54149Gb-i00217Gh	11	40	i43181Gh-i16165Gh	8.52	3.56	1.67	1.90

	Imeq-LY-15	III	2	25	i02761Gh-i02712Gh	13	10	i45163Gh-i30934Gh	10.22	3.33	2.57	0.76
	Imeq-LY-16	III	9	70	i00393Gh-i04801Gh	13	10	i45163Gh-i30934Gh	10.12	3.71	3.06	0.65
	Imeq-LY-17	III	7	65	i14398Gh-i01824Gh	13	15	i30934Gh-i18151Gh	10.68	2.94	2.21	0.72
	Imeq-LY-18	III	15	0	i02955Gh-i02314Gh	17	50	i14844Gh-i03522Gh	9.67	3.00	2.50	0.50
	Imeq-LY-19	III	8	10	i24295Gh-i00234Gh	18	0	i13766Gh-i13754Gh	8.84	3.07	1.75	1.32
	Imeq-LY-20	II	15	20	**i29719Gh-i64628Gm**	18	0	i13766Gh-i13754Gh	8.53	2.56	1.78	0.78
	Imeq-LY-21	III	17	55	i03522Gh-i03688Gh	18	0	i13766Gh-i13754Gh	11.53	3.18	1.64	1.53
	Imeq-LY-22	III	7	20	i01696Gh-i57601Gb	20	10	i39228Gh-i34769Gh	11.45	3.95	2.68	1.27
	Imeq-LY-23	III	21	50	i35971Gh-i47631Gh	21	60	i22642Gh-i41613Gh	10.99	1.19	1.19	0.00
	Imeq-LY-24	III	5	65	i37142Gh-i48326Gh	22	50	i44682Gh-i25111Gh	9.87	3.18	2.09	1.09
	Imeq-LY-25	III	6	55	i06396Gh-i06056Gh	24	30	i15200Gh-i04548Gh	9.00	2.64	1.49	1.15
	Imeq-LY-26	III	4	10	i31054Gh-i41405Gh	24	45	i26213Gh-i00339Gh	8.90	2.45	1.61	0.84
	Imeq-LY-27	III	12	15	i40974Gh-i48211Gh	25	20	i41210Gh-i42629Gh	9.02	2.98	1.55	1.43
	Imeq-LY-28	III	17	40	i03508Gh-i18575Gh	25	25	i27022Gh-i11449Gh	8.53	2.86	2.13	0.74
	Imeq-LY-29	III	4	15	i41085Gh-i38159Gh	26	5	i08062Gh-i33827Gh	8.55	2.60	1.90	0.70
	Imeq-LY-30	III	7	65	i14398Gh-i01824Gh	26	20	i37251Gh-i23249Gh	8.79	2.83	1.51	1.32
	Imeq-LY-31	III	19	20	i08987Gh-i09220Gh	26	20	i37251Gh-i23249Gh	8.82	3.07	2.34	0.73
	Imeq-LY-32	III	21	60	i22642Gh-i41613Gh	26	45	i22171Gh-i00945Gh	9.20	3.05	1.42	1.63
					**HSBCF_1 population**							
FB	B_1meq-FB-1	III	8	25	i26219Gh-i32773Gh	9	10	i05758Gh-i19700Gh	6.87	2.74	2.14	0.60
	B_1meq-FB-2	III	9	45	i46552Gh-i24387Gh	13	15	i30934Gh-i18151Gh	6.68	2.39	1.75	0.64
	B_1meq-FB-3	III	7	65	i14398Gh-i01824Gh	13	65	i29310Gh-i00241Gh	6.07	2.14	0.89	1.25
	B_1meq-FB-4	III	3	85	i31859Gh-i42939Gh	15	35	i17844Gh-i37620Gh	6.30	2.56	2.20	0.36
	B_1meq-FB-5	III	6	40	i06505Gh-i23722Gh	20	10	i39228Gh-i34769Gh	7.13	2.76	1.38	1.38
	B_1meq-FB-6	III	5	30	i53001Gb-i08984Gh	20	40	i37554Gh-i47006Gh	6.95	2.85	2.49	0.36
	B_1meq-FB-7	III	19	30	i16566Gh-i08941Gh	20	40	i37554Gh-i47006Gh	6.97	2.75	2.50	0.26

	B_1meq-FB-8	III	5	50	i16666Gh-i09095Gh	23	0	i06287Gh-i06171Gh	7.38	2.95	1.37	1.58
	B_1meq-FB-9	III	16	70	i54704Gb-i01693Gh	24	10	i04567Gh-i15176Gh	6.09	2.15	1.43	0.72
	B_1meq-FB-10	III	9	75	i05825Gh-i14639Gh	26	5	i08062Gh-i33827Gh	6.40	2.60	1.14	1.46
	B_1meq-FB-11	II	6	35	**i06036Gh-i06037Gh**	26	15	i25512Gh-i07941Gh	6.49	2.52	2.15	0.37
	B_1meq-FB-12	III	14	60	i26838Gh-i01129Gh	26	40	i36067Gh-i08578Gh	6.41	2.26	1.79	0.46
BN	B_1meq-BN-1	III	1	45	i02245Gh-i44115Gh	2	60	i02432Gh-i14623Gh	6.22	0.29	0.27	0.02
	B_1meq-BN-2	III	1	45	i02245Gh-i44115Gh	7	30	i38586Gh-i26820Gh	6.97	0.27	0.16	0.11
	B_1meq-BN-3	III	7	60	i14398Gh-i01824Gh	8	15	i04570Gh-i04506Gh	6.78	2.81	1.83	0.98
	B_1meq-BN-4	III	3	45	i35903Gh-i20966Gh	8	20	i25482Gh-i25868Gh	7.07	2.92	1.77	1.16
	B_1meq-BN-5	III	8	25	i26219Gh-i32773Gh	9	10	i05758Gh-i19700Gh	6.13	2.60	1.36	1.24
	B_1meq-BN-6	III	6	30	i34827Gh-i15830Gh	9	45	i46552Gh-i24387Gh	6.66	2.69	1.26	1.43
	B_1meq-BN-7	III	1	15	i02201Gh-i27043Gh	11	20	i36064Gh-i44818Gh	6.49	1.62	0.96	0.66
	B_1meq-BN-8	III	5	60	i52543Gb-i35080Gh	11	20	i36064Gh-i44818Gh	6.32	2.46	1.87	0.59
	B_1meq-BN-9	III	7	25	i33174Gh-i01631Gh	13	55	i21560Gh-i46408Gh	6.03	1.61	1.33	0.28
	B_1meq-BN-10	III	8	30	i54149Gb-i00217Gh	14	60	i26838Gh-i01129Gh	10.48	4.12	2.48	1.64
	B_1meq-BN-11	III	11	15	i01036Gh-i07468Gh	15	30	i02459Gh-i02486Gh	6.18	2.70	1.69	1.02
	B_1meq-BN-12	III	15	20	i29719Gh-i64628Gm	15	35	i17844Gh-i37620Gh	7.42	2.76	0.98	1.78
	B_1meq-BN-13	III	14	60	i26838Gh-i01129Gh	15	40	i02315Gh-i07215Gh	7.83	3.40	1.68	1.73
	B_1meq-BN-14	III	14	25	i15375Gh-i05040Gh	16	55	i21384Gh-i44137Gh	6.64	2.55	1.16	1.39
	B_1meq-BN-15	III	13	35	i37089Gh-i41278Gh	17	15	i03341Gh-i28038Gh	6.09	2.06	0.27	1.80
	B_1meq-BN-16	III	18	25	i13146Gh-i20346Gh	18	50	i13451Gh-i38577Gh	6.69	1.50	0.71	0.79
	B_1meq-BN-17	III	3	45	i35903Gh-i20966Gh	18	70	i49258Gh-i13532Gh	8.56	3.15	1.03	2.12
	B_1meq-BN-18	III	5	50	i16666Gh-i09095Gh	18	70	i49258Gh-i13532Gh	7.16	2.73	1.67	1.06
	B_1meq-BN-19	III	19	30	i16566Gh-i08941Gh	20	5	i00478Gh-i11539Gh	10.20	3.76	1.05	2.71
	B_1meq-BN-20	III	6	40	i06505Gh-i23722Gh	20	10	i39228Gh-i34769Gh	9.41	3.75	1.48	2.27
	B_1meq-BN-21	III	18	5	i13754Gh-i13145Gh	20	20	i26356Gh-i11706Gh	6.17	2.48	1.93	0.55

	B_1meq-BN-22	III	20	25	i25398Gh-i11734Gh	20	35	i40942Gh-i35292Gh	10.39	0.52	0.52	0.00
	B_1meq-BN-23	III	3	70	i22169Gh-i33635Gh	20	40	i37554Gh-i47006Gh	6.33	2.45	1.81	0.64
	B_1meq-BN-24	III	1	5	i21210Gh-i35065Gh	22	35	i12538Gh-i12818Gh	6.26	2.54	1.07	1.47
	B_1meq-BN-25	III	18	80	i43889Gh-i25079Gh	22	45	i44682Gh-i25111Gh	7.03	2.71	0.83	1.88
	B_1meq-BN-26	III	3	45	i35903Gh-i20966Gh	24	30	i15200Gh-i04548Gh	6.77	2.64	1.95	0.69
	B_1meq-BN-27	III	14	60	i26838Gh-i01129Gh	24	30	i15200Gh-i04548Gh	8.81	3.82	2.40	1.41
	B_1meq-BN-28	III	16	10	i13939Gh-i01279Gh	24	45	i26213Gh-i00339Gh	6.11	2.36	1.55	0.81
	B_1meq-BN-29	III	3	85	i31859Gh-i42939Gh	25	20	i41210Gh-i42629Gh	7.48	3.07	1.62	1.45
	B_1meq-BN-30	III	20	20	i26356Gh-i11706Gh	25	20	i41210Gh-i42629Gh	6.99	3.02	0.83	2.19
	B_1meq-BN-31	III	7	30	i38586Gh-i26820Gh	25	45	i21894Gh-i19984Gh	6.64	2.41	0.97	1.44
	B_1meq-BN-32	III	6	10	i06061Gh-i05824Gh	26	5	i08062Gh-i33827Gh	6.34	2.25	1.41	0.84
	B_1meq-BN-33	III	9	75	i05825Gh-i14639Gh	26	10	i33827Gh-i25834Gh	10.17	4.10	1.74	2.36
	B_1meq-BN-34	III	10	10	i43940Gh-i25267Gh	26	20	i37251Gh-i23249Gh	7.50	2.72	1.82	0.90
	B_1meq-BN-35	III	14	40	i22707Gh-i38937Gh	26	45	i22171Gh-i00945Gh	7.75	3.28	1.65	1.63
BW	B_1meq-BW-1	III	3	115	i05514Gh-i29314Gh	6	30	i34827Gh-i15830Gh	6.82	2.52	0.24	2.28
	B_1meq-BW-2	III	6	15	i19214Gh-i31843Gh	20	25	i25398Gh-i11734Gh	6.02	2.24	1.61	0.63
	B_1meq-BW-3	III	14	35	i04933Gh-i43400Gh	22	35	i12538Gh-i12818Gh	6.30	2.37	0.93	1.43
	B_1meq-BW-4	III	17	0	i14907Gh-i14878Gh	22	40	i12810Gh-i17697Gh	7.89	2.85	2.17	0.68
LP	B_1meq-LP-1	III	9	50	i35858Gh-i03687Gh	10	15	i25267Gh-i30274Gh	6.10	2.19	2.10	0.09
	B_1meq-LP-2	III	8	0	i63682Gm-i37825Gh	10	45	i26780Gh-i33011Gh	7.29	3.40	1.32	2.08
	B_1meq-LP-3	III	7	20	i01696Gh-i57601Gb	14	25	i15375Gh-i05040Gh	7.26	3.07	2.03	1.04
	B_1meq-LP-4	III	4	30	i10502Gh-i36496Gh	16	35	i01640Gh-i00384Gh	6.11	2.66	1.33	1.33
	B_1meq-LP-5	III	14	35	i04933Gh-i43400Gh	18	110	i45991Gh-i13081Gh	8.40	3.65	3.41	0.24
	B_1meq-LP-6	III	17	15	i03341Gh-i28038Gh	19	35	i50235Gb-i52709Gb	6.06	2.23	2.19	0.04
	B_1meq-LP-7	III	11	30	i20872Gh-i40251Gh	20	80	i11915Gh-i11478Gh	6.05	2.42	2.31	0.11
	B_1meq-LP-8	III	4	30	i10502Gh-i36496Gh	21	60	i22642Gh-i41613Gh	7.89	3.46	2.33	1.13

	B_1meq-LP-9	III	15	45	i02315Gh-i07215Gh	21	65	i22642Gh-i41613Gh	6.08	2.42	1.84	0.58
	B_1meq-LP-10	III	2	95	i07717Gh-i09654Gh	24	55	i14999Gh-i14993Gh	6.04	2.60	2.05	0.55
	B_1meq-LP-11	III	18	35	i32883Gh-i13851Gh	26	45	i22171Gh-i00945Gh	6.15	2.59	1.88	0.70
SY	B_1meq-SY-1	II	1	25	**i14664Gh-i02994Gh**	4	30	i10502Gh-i36496Gh	6.70	2.90	1.60	1.30
	B_1meq-SY-2	III	6	10	i06061Gh-i05824Gh	6	35	i06036Gh-i06037Gh	6.87	2.71	2.13	0.58
	B_1meq-SY-3	III	6	35	i06036Gh-i06037Gh	7	10	i24917Gh-i26814Gh	6.57	2.91	1.02	1.89
	B_1meq-SY-4	III	6	35	i06036Gh-i06037Gh	8	10	i24295Gh-i00234Gh	10.60	4.36	1.37	2.99
	B_1meq-SY-5	III	4	25	i10502Gh-i36496Gh	8	25	i26219Gh-i32773Gh	7.89	3.32	0.75	2.58
	B_1meq-SY-6	III	9	10	i05758Gh-i19700Gh	9	15	i40336Gh-i07864Gh	7.70	2.67	1.49	1.18
	B_1meq-SY-7	III	6	30	i34827Gh-i15830Gh	9	45	i46552Gh-i24387Gh	7.42	2.97	1.04	1.92
	B_1meq-SY-8	III	5	25	i47720Gh-i37479Gh	11	0	i52789Gb-i07420Gh	6.66	2.21	0.91	1.31
	B_1meq-SY-9	III	3	70	i22169Gh-i33635Gh	13	25	i13079Gh-i36296Gh	6.57	2.46	1.28	1.18
	B_1meq-SY-10	II	3	115	i05514Gh-i29314Gh	14	55	**i23629Gh-i15587Gh**	6.58	2.46	0.86	1.60
	B_1meq-SY-11	III	13	25	i13079Gh-i36296Gh	15	25	i18410Gh-i38002Gh	7.34	2.86	0.98	1.88
	B_1meq-SY-12	III	15	25	i18410Gh-i38002Gh	15	30	i02459Gh-i02486Gh	9.34	3.73	2.45	1.28
	B_1meq-SY-13	III	11	10	i47563Gh-i01036Gh	17	15	i03341Gh-i28038Gh	6.80	2.28	1.00	1.28
	B_1meq-SY-14	III	1	10	i53010Gb-i21390Gh	18	5	i13754Gh-i13145Gh	7.26	2.83	1.58	1.25
	B_1meq-SY-15	III	2	50	i02554Gh-i02563Gh	18	5	i13754Gh-i13145Gh	9.18	3.17	2.11	1.07
	B_1meq-SY-16	III	5	50	i16666Gh-i09095Gh	18	50	i13451Gh-i38577Gh	7.16	2.86	1.57	1.28
	B_1meq-SY-17	III	15	25	i18410Gh-i38002Gh	19	20	i08987Gh-i09220Gh	7.39	2.90	0.92	1.98
	B_1meq-SY-18	III	6	40	i06505Gh-i23722Gh	20	10	i39228Gh-i34769Gh	13.36	5.18	2.77	2.41
	B_1meq-SY-19	III	18	5	i13754Gh-i13145Gh	20	25	i25398Gh-i11734Gh	9.41	3.83	1.30	2.52
	B_1meq-SY-20	III	19	20	i08987Gh-i09220Gh	20	40	i37554Gh-i47006Gh	8.13	3.38	1.48	1.89
	B_1meq-SY-21	III	20	10	i39228Gh-i34769Gh	20	45	i17500Gh-i47439Gh	9.80	2.65	1.60	1.05
	B_1meq-SY-22	III	5	50	i16666Gh-i09095Gh	22	40	i12810Gh-i17697Gh	8.60	3.37	1.96	1.41
	B_1meq-SY-23	III	8	15	i04570Gh-i04506Gh	22	50	i44682Gh-i25111Gh	10.51	4.43	1.22	3.20

	B_1meq-SY-24	III	22	50	i44682Gh-i25111Gh	24	30	i15200Gh-i04548Gh	6.26	2.57	0.95	1.62
	B_1meq-SY-25	III	19	40	i08786Gh-i00558Gh	25	35	i11465Gh-i39910Gh	9.43	3.16	2.18	0.98
	B_1meq-SY-26	III	17	50	i14844Gh-i03522Gh	25	45	i21894Gh-i19984Gh	6.52	1.95	1.25	0.70
	B_1meq-SY-27	III	6	10	i06061Gh-i05824Gh	26	10	i33827Gh-i25834Gh	6.41	2.43	1.34	1.08
	B_1meq-SY-28	III	10	15	i25267Gh-i30274Gh	26	20	i37251Gh-i23249Gh	7.54	3.04	1.10	1.95
	B_1meq-SY-29	III	9	0	i40221Gh-i15598Gh	26	45	i22171Gh-i00945Gh	9.62	3.26	1.11	2.15
	B_1meq-SY-30	III	5	55	i09147Gh-i42098Gh	26	50	i16464Gh-i28856Gh	6.62	1.19	0.66	0.52
	B_1meq-SY-31	II	14	55	**i23629Gh-i15587Gh**	26	50	i16464Gh-i28856Gh	6.83	1.86	0.78	1.08
LY	B_1meq-LY-1	III	6	10	i06061Gh-i05824Gh	6	35	i06036Gh-i06037Gh	7.43	2.96	2.07	0.89
	B_1meq-LY-2	III	6	35	i06036Gh-i06037Gh	8	10	i24295Gh-i00234Gh	11.72	4.74	1.38	3.36
	B_1meq-LY-3	III	4	25	i10502Gh-i36496Gh	8	25	i26219Gh-i32773Gh	7.58	3.22	0.81	2.42
	B_1meq-LY-4	III	9	10	i05758Gh-i19700Gh	9	15	i40336Gh-i07864Gh	8.55	2.71	1.69	1.02
	B_1meq-LY-5	III	3	70	i22169Gh-i33635Gh	13	25	i13079Gh-i36296Gh	7.88	3.22	1.91	1.31
	B_1meq-LY-6	II	14	45	i27231Gh-i36385Gh	14	50	**i34963Gh-i44045Gh**	8.69	0.25	0.25	0.00
	B_1meq-LY-7	III	11	0	i52789Gb-i07420Gh	15	5	i02306Gh-i02317Gh	8.61	3.79	2.19	1.60
	B_1meq-LY-8	III	13	25	i13079Gh-i36296Gh	15	25	i18410Gh-i38002Gh	8.53	3.49	1.29	2.20
	B_1meq-LY-9	III	15	25	i18410Gh-i38002Gh	15	30	i02459Gh-i02486Gh	8.34	3.37	2.36	1.01
	B_1meq-LY-10	III	1	10	i53010Gb-i21390Gh	18	5	i13754Gh-i13145Gh	7.91	3.16	1.45	1.71
	B_1meq-LY-11	III	2	50	i02554Gh-i02563Gh	18	5	i13754Gh-i13145Gh	10.57	3.67	2.29	1.38
	B_1meq-LY-12	III	5	50	i16666Gh-i09095Gh	18	50	i13451Gh-i38577Gh	8.38	3.48	1.57	1.92
	B_1meq-LY-13	III	13	25	i13079Gh-i36296Gh	18	110	i45991Gh-i13081Gh	7.07	0.54	0.54	0.00
	B_1meq-LY-14	III	15	25	i18410Gh-i38002Gh	19	20	i08987Gh-i09220Gh	8.74	3.43	1.25	2.18
	B_1meq-LY-15	III	2	85	i38985Gh-i30800Gh	20	0	i17414Gh-i17417Gh	7.19	2.33	1.15	1.18
	B_1meq-LY-16	III	6	40	i06505Gh-i23722Gh	20	10	i39228Gh-i34769Gh	13.20	5.13	2.77	2.36
	B_1meq-LY-17	III	18	5	i13754Gh-i13145Gh	20	25	i25398Gh-i11734Gh	10.41	4.31	1.35	2.96
	B_1meq-LY-18	III	19	20	i08987Gh-i09220Gh	20	40	i37554Gh-i47006Gh	7.56	3.33	1.36	1.98

	B_1meq-LY-19	III	20	10	i39228Gh-i34769Gh	20	45	i17500Gh-i47439Gh	11.32	3.17	1.78	1.39
	B_1meq-LY-20	III	8	15	i04570Gh-i04506Gh	22	50	i44682Gh-i25111Gh	9.85	4.17	0.79	3.38
	B_1meq-LY-21	III	19	40	i08786Gh-i00558Gh	25	35	i11465Gh-i39910Gh	11.12	3.66	2.36	1.29
	B_1meq-LY-22	III	1	20	i47599Gh-i49208Gh	26	50	i16464Gh-i28856Gh	7.57	2.44	0.98	1.46
	B_1meq-LY-23	III	2	85	i38985Gh-i30800Gh	26	50	i16464Gh-i28856Gh	7.56	3.36	1.38	1.97
	B_1meq-LY-24	III	5	55	i09147Gh-i42098Gh	26	50	i16464Gh-i28856Gh	8.98	1.77	1.14	0.63
	B_1meq-LY-25	III	6	45	i23722Gh-i37862Gh	26	50	i16464Gh-i28856Gh	7.80	3.02	0.85	2.17
	B_1meq-LY-26	III	13	30	i24929Gh-i13848Gh	26	50	i16464Gh-i28856Gh	8.75	2.00	1.07	0.93
	B_1meq-LY-27	III	14	55	i23629Gh-i15587Gh	26	50	i16464Gh-i28856Gh	8.63	2.24	1.23	1.02
	B_1meq-LY-28	III	15	5	i02306Gh-i02317Gh	26	50	i16464Gh-i28856Gh	8.49	3.63	1.39	2.24
	B_1meq-LY-29	III	16	20	i24194Gh-i40100Gh	26	50	i16464Gh-i28856Gh	8.32	1.62	1.04	0.58
	B_1meq-LY-30	III	17	55	i03522Gh-i03688Gh	26	50	i16464Gh-i28856Gh	8.72	2.28	1.08	1.20
	B_1meq-LY-31	III	18	75	i13532Gh-i43889Gh	26	50	i16464Gh-i28856Gh	7.60	0.62	0.62	0.00
	B_1meq-LY-32	III	20	5	i00478Gh-i11539Gh	26	50	i16464Gh-i28856Gh	8.01	2.53	1.18	1.36
	B_1meq-LY-33	III	22	20	i12927Gh-i12929Gh	26	50	i16464Gh-i28856Gh	7.88	0.82	0.82	0.00
**MARBCF_1 population**												
FB	B_2meq-FB-1	III	3	40	i00971Gh-i46613Gh	7	15	i46540Gh-i01765Gh	6.06	2.52	2.41	0.11
	B_2meq-FB-2	III	7	15	i46540Gh-i01765Gh	8	55	i01126Gh-i04719Gh	6.32	2.66	2.47	0.19
	B_2meq-FB-3	III	8	5	i31145Gh-i40270Gh	13	40	i46668Gh-i00187Gh	6.80	3.01	2.70	0.31
	B_2meq-FB-4	III	13	35	i37089Gh-i41278Gh	14	40	i22707Gh-i38937Gh	6.04	2.67	1.87	0.80
	B_2meq-FB-5	III	16	25	i29957Gh-i01669Gh	17	40	i03508Gh-i18575Gh	6.26	2.04	1.36	0.68
	B_2meq-FB-6	III	14	40	i22707Gh-i38937Gh	22	50	i44682Gh-i25111Gh	6.31	2.74	1.95	0.80
	B_2meq-FB-7	III	18	0	i13766Gh-i13754Gh	25	5	i00735Gh-i41210Gh	6.01	1.66	1.63	0.03
	B_2meq-FB-8	III	25	5	i00735Gh-i41210Gh	25	40	i22495Gh-i55440Gb	6.39	2.26	2.22	0.04
	B_2meq-FB-9	III	3	75	i43226Gh-i45963Gh	26	50	i16464Gh-i28856Gh	6.44	2.66	2.52	0.14
	B_2meq-FB-10	III	14	25	i15375Gh-i05040Gh	26	50	i16464Gh-i28856Gh	6.41	2.50	2.28	0.22

	B_2meq-FB-11	III	18	5	i13754Gh-i13145Gh	26	50	i16464Gh-i28856Gh	6.58	2.56	2.33	0.23
	B_2meq-FB-12	III	20	40	i37554Gh-i47006Gh	26	50	i16464Gh-i28856Gh	6.72	2.40	2.06	0.34
	B_2meq-FB-13	III	26	40	i36067Gh-i08578Gh	26	50	i16464Gh-i28856Gh	6.36	2.19	1.88	0.31
BN	B_2meq-BN-1	III	3	110	i20709Gh-i23313Gh	8	0	i63682Gm-i37825Gh	7.47	1.49	0.90	0.59
	B_2meq-BN-2	III	4	5	i26515Gh-i43091Gh	11	40	i43181Gh-i16165Gh	6.11	2.64	1.51	1.13
	B_2meq-BN-3	III	5	65	i37142Gh-i48326Gh	11	40	i43181Gh-i16165Gh	6.34	2.45	0.71	1.73
	B_2meq-BN-4	III	4	5	i26515Gh-i43091Gh	13	10	i45163Gh-i30934Gh	7.12	2.57	1.35	1.22
	B_2meq-BN-5	III	4	15	i41085Gh-i38159Gh	14	20	i15345Gh-i18849Gh	6.23	2.74	1.11	1.62
	B_2meq-BN-6	III	11	15	i01036Gh-i07468Gh	17	0	i14907Gh-i14878Gh	6.86	2.93	1.60	1.33
	B_2meq-BN-7	III	2	70	i00890Gh-i02271Gh	19	0	i09067Gh-i09082Gh	6.27	2.37	1.05	1.32
	B_2meq-BN-8	III	18	110	i45991Gh-i13081Gh	19	10	i08933Gh-i28797Gh	7.90	2.85	1.87	0.97
	B_2meq-BN-9	III	9	10	i05758Gh-i19700Gh	20	10	i39228Gh-i34769Gh	6.29	2.27	2.07	0.20
	B_2meq-BN-10	III	17	40	i03508Gh-i18575Gh	20	30	i11735Gh-i44450Gh	8.59	2.80	1.62	1.18
	B_2meq-BN-11	III	7	65	i14398Gh-i01824Gh	20	35	i40942Gh-i35292Gh	7.01	1.96	0.57	1.39
	B_2meq-BN-12	III	8	25	i26219Gh-i32773Gh	20	65	i11915Gh-i11478Gh	6.00	1.78	0.41	1.38
	B_2meq-BN-13	III	14	15	i15343Gh-i31037Gh	22	30	i12906Gh-i17853Gh	6.62	2.39	0.98	1.41
	B_2meq-BN-14	III	6	55	i06396Gh-i06056Gh	22	50	i44682Gh-i25111Gh	6.16	1.95	1.31	0.64
	B_2meq-BN-15	III	19	35	i50235Gb-i52709Gb	25	40	i22495Gh-i55440Gb	6.98	2.58	1.45	1.13
	B_2meq-BN-16	III	13	55	i21560Gh-i46408Gh	25	45	i21894Gh-i19984Gh	6.25	2.50	0.41	2.09
	B_2meq-BN-17	III	5	25	i47720Gh-i37479Gh	26	15	i25512Gh-i07941Gh	6.19	2.20	2.16	0.03
BW	B_2meq-BW-1	III	1	45	i02245Gh-i44115Gh	14	35	i04933Gh-i43400Gh	6.26	0.96	0.64	0.32
	B_2meq-BW-2	III	15	20	i29719Gh-i64628Gm	16	15	i14406Gh-i01766Gh	6.83	2.97	1.91	1.05
	B_2meq-BW-3	III	6	10	i06061Gh-i05824Gh	21	70	i33529Gh-i43642Gh	6.19	2.58	1.41	1.17
LP	B_2meq-LP-1	III	8	10	i24295Gh-i00234Gh	10	45	i26780Gh-i33011Gh	6.57	2.46	1.76	0.70
SY	B_2meq-SY-1	III	1	0	i33646Gh-i40884Gh	3	105	i20709Gh-i23313Gh	6.39	2.78	0.17	2.61
	B_2meq-SY-2	III	1	0	i33646Gh-i40884Gh	9	5	i25689Gh-i17373Gh	6.40	2.82	0.12	2.70

	B_2meq-SY-3	III	2	95	i07717Gh-i09654Gh	9	75	i05825Gh-i14639Gh	6.25	2.58	1.14	1.43
	B_2meq-SY-4	III	8	10	i24295Gh-i00234Gh	10	45	i26780Gh-i33011Gh	6.44	2.25	1.00	1.25
	B_2meq-SY-5	II	8	25	i26219Gh-i32773Gh	11	0	**i52789Gb-i07420Gh**	6.39	2.70	2.54	0.16
	B_2meq-SY-6	III	7	65	i14398Gh-i01824Gh	11	20	i36064Gh-i44818Gh	6.31	2.43	1.37	1.06
	B_2meq-SY-7	III	11	35	i40251Gh-i07190Gh	18	115	i13081Gh-i13709Gh	6.66	2.45	0.83	1.63
	B_2meq-SY-8	III	15	45	i02315Gh-i07215Gh	18	115	i13081Gh-i13709Gh	7.11	2.36	0.54	1.82
	B_2meq-SY-9	III	18	30	i32883Gh-i13851Gh	19	0	i09067Gh-i09082Gh	7.26	2.76	1.68	1.08
	B_2meq-SY-10	II	21	20	**i07714Gh-i38909Gh**	21	25	i22367Gh-i47711Gh	6.23	0.68	0.68	0.00
	B_2meq-SY-11	III	16	15	i14406Gh-i01766Gh	21	45	i07446Gh-i16079Gh	7.43	3.00	2.04	0.96
	B_2meq-SY-12	III	5	40	i08988Gh-i45534Gh	22	45	i44682Gh-i25111Gh	6.24	2.49	1.10	1.39
	B_2meq-SY-13	III	7	30	i38586Gh-i26820Gh	23	0	i06287Gh-i06171Gh	6.61	2.62	1.35	1.27
	B_2meq-SY-14	III	11	40	i43181Gh-i16165Gh	24	50	i38401Gh-i04575Gh	6.50	2.22	1.41	0.81
	B_2meq-SY-15	III	21	65	i22642Gh-i41613Gh	24	55	i14999Gh-i14993Gh	7.62	2.86	0.65	2.21
	B_2meq-SY-16	III	5	30	i53001Gb-i08984Gh	24	60	i03705Gh-i33113Gh	6.61	2.45	2.29	0.16
	B_2meq-SY-17	III	7	30	i38586Gh-i26820Gh	25	60	i17145Gh-i10628Gh	7.17	2.59	1.47	1.12
	B_2meq-SY-18	III	10	45	i26780Gh-i33011Gh	26	5	i08062Gh-i33827Gh	7.30	2.68	1.87	0.81
	B_2meq-SY-19	III	13	45	i13299Gh-i35111Gh	26	15	i25512Gh-i07941Gh	8.47	3.05	2.10	0.95
LY	B_2meq-LY-1	III	2	95	i07717Gh-i09654Gh	9	75	i05825Gh-i14639Gh	6.23	2.52	0.92	1.60
	B_2meq-LY-2	III	7	20	i01696Gh-i57601Gb	12	10	i40974Gh-i48211Gh	6.27	1.77	0.96	0.81
	B_2meq-LY-3	III	15	45	i02315Gh-i07215Gh	18	75	i13532Gh-i43889Gh	8.07	2.59	0.76	1.83
	B_2meq-LY-4	II	11	0	**i52789Gb-i07420Gh**	18	115	i13081Gh-i13709Gh	7.24	2.59	1.08	1.51
	B_2meq-LY-5	III	2	25	i02761Gh-i02712Gh	19	0	i09067Gh-i09082Gh	6.36	2.32	0.31	2.01
	B_2meq-LY-6	III	18	30	i32883Gh-i13851Gh	19	0	i09067Gh-i09082Gh	7.72	2.82	1.43	1.39
	B_2meq-LY-7	II	2	35	i02523Gh-i27649Gh	21	20	**i07714Gh-i38909Gh**	7.03	1.53	1.02	0.52
	B_2meq-LY-8	II	3	110	i20709Gh-i23313Gh	21	20	**i07714Gh-i38909Gh**	6.60	2.38	0.93	1.45
	B_2meq-LY-9	II	4	30	i10502Gh-i36496Gh	21	20	**i07714Gh-i38909Gh**	6.43	2.80	1.06	1.74

B_2meq-LY-10	II	13	25	i13079Gh-i36296Gh	21	20	**i07714Gh-i38909Gh**	6.55	1.54	0.45	1.10
B_2meq-LY-11	II	20	80	i11915Gh-i11478Gh	21	20	**i07714Gh-i38909Gh**	6.76	2.36	1.18	1.18
B_2meq-LY-12	II	21	20	**i07714Gh-i38909Gh**	21	25	i22367Gh-i47711Gh	8.56	0.73	0.73	0.00
B_2meq-LY-13	III	16	15	i14406Gh-i01766Gh	21	45	i07446Gh-i16079Gh	7.86	3.03	1.85	1.18
B_2meq-LY-14	III	7	30	i38586Gh-i26820Gh	23	0	i06287Gh-i06171Gh	6.03	2.39	1.08	1.31
B_2meq-LY-15	III	11	40	i43181Gh-i16165Gh	24	50	i38401Gh-i04575Gh	6.75	2.21	1.42	0.79
B_2meq-LY-16	III	21	65	i22642Gh-i41613Gh	24	55	i14999Gh-i14993Gh	8.21	3.02	0.64	2.38
B_2meq-LY-17	III	5	30	i53001Gb-i08984Gh	24	60	i03705Gh-i33113Gh	6.37	2.30	2.11	0.19
B_2meq-LY-18	III	7	30	i38586Gh-i26820Gh	25	60	i17145Gh-i10628Gh	7.01	2.47	1.32	1.15
B_2meq-LY-19	III	10	45	i26780Gh-i33011Gh	26	5	i08062Gh-i33827Gh	8.04	2.86	1.82	1.04
B_2meq-LY-20	III	13	45	i13299Gh-i35111Gh	26	15	i25512Gh-i07941Gh	8.88	3.03	2.03	1.01

[a] FB: number of fruit branches per plant; BN: number of bolls per plant; BW: boll weight; LP: lint percentage; SY: seed cotton yield; LY: lint yield

[b] Type of epistasis: (I) two loci with m-QTL, (II) one loci with m-QTL and the other loci without significant m-QTL and (III) two loci without significant m-QTL

[c] Chi and Chj represent the chromosome number of the loci being tested in the analysis

[d] Position of e-QTL located on chromosome: as cM distance from the top of each chromosome

[e] Flanking markers in bold are those flanking m-QTLs identified by ICIM in additional Table S6

[f] A LOD threshold was used for declaration of QTL based on 1000 permutations at as significance level of 0.01

[g] PV: the phenotypic variation that the total epistasis effect explained; PV(AA): the phenotypic variation that the main epistasis effect explained; PV (AAE): the phenotypic variation that the environmental interaction of the epistasis effect explained
