# Supplementary material for: Genetic basis of heterosis for yield and yield components explored by QTL mapping across four genetic populations in upland cotton
Source: BMC Genomics. 2018 Dec 12;19:910. doi: 10.1186/s12864-018-5289-2 (PMC6292039; doi:10.1186/s12864-018-5289-2)
Supplement: Supplementary file 10 — Table S9. Epistatic effects and environmental interactions detected for yield and yield components in IF2MPH, HSBCF1MPH, and MARBCF1MPH datasets using the ICIM method. (PDF 631 kb) [file 12864_2018_5289_MOESM10_ESM.pdf]

Table S9 Epistatic effects and environmental interactions detected for yield and yield components in IF_2MPH, HSBCF_1MPH, and MARBCF_1MPH datasets using the ICIM method

Traits[a]	e-QTL	Type of epistasis[b]	Chr.i[c]	Position1[d]	Flanking markers 1[e]	Chr.j[c]	Position2[d]	Flanking markers 2[e]	LOD[f]	PV[g]	PV(AA)[g]	PV(AAE)[g]
					**MPHIF_2s**							
FB	IMmeq-FB-1	III	1	5	i21210Gh-i35065Gh	5	55	i09147Gh-i42098Gh	9.07	3.10	2.36	0.73
	IMmeq-FB-2	III	3	80	i43226Gh-i45963Gh	13	0	i17985Gh-i12984Gh	8.59	2.89	0.56	2.33
	IMmeq-FB-3	III	13	0	i17985Gh-i12984Gh	15	0	i02955Gh-i02314Gh	8.95	2.73	0.59	2.14
	IMmeq-FB-4	III	17	40	i03508Gh-i18575Gh	18	35	i32883Gh-i13851Gh	10.08	3.19	1.28	1.91
	IMmeq-FB-5	III	5	40	i08988Gh-i45534Gh	20	5	i00478Gh-i11539Gh	8.87	0.32	0.10	0.22
	IMmeq-FB-6	III	8	10	i24295Gh-i00234Gh	20	10	i39228Gh-i34769Gh	11.30	3.90	1.46	2.43
	IMmeq-FB-7	III	9	55	i29408Gh-i03595Gh	20	25	i25398Gh-i11734Gh	9.25	2.66	1.95	0.71
	IMmeq-FB-8	III	4	20	i00135Gh-i47058Gh	21	45	i07446Gh-i16079Gh	12.72	0.11	0.01	0.10
	IMmeq-FB-9	III	15	25	i18410Gh-i38002Gh	21	50	i35971Gh-i47631Gh	8.78	2.89	1.34	1.56
	IMmeq-FB-10	III	21	60	i22642Gh-i41613Gh	22	20	i12927Gh-i12929Gh	9.23	2.80	1.63	1.17
	IMmeq-FB-11	III	8	5	i31145Gh-i40270Gh	22	45	i44682Gh-i25111Gh	9.07	3.01	2.37	0.64
	IMmeq-FB-12	III	12	0	i40974Gh-i48211Gh	24	10	i04567Gh-i15176Gh	9.04	2.14	1.20	0.93
	IMmeq-FB-13	III	20	10	i39228Gh-i34769Gh	24	35	i04535Gh-i04423Gh	10.64	3.41	2.28	1.13
	IMmeq-FB-14	III	18	0	i13766Gh-i13754Gh	25	25	i27022Gh-i11449Gh	8.89	3.34	1.52	1.82
	IMmeq-FB-15	III	7	10	i24917Gh-i26814Gh	25	40	i22495Gh-i55440Gb	9.10	2.95	2.63	0.32
	IMmeq-FB-16	III	13	0	i17985Gh-i12984Gh	25	40	i22495Gh-i55440Gb	9.23	3.20	1.01	2.19
	IMmeq-FB-17	II	5	65	**i37142Gh-i48326Gh**	25	45	i21894Gh-i19984Gh	8.73	2.90	1.91	0.99
	IMmeq-FB-18	III	20	80	i11915Gh-i11478Gh	25	50	i40453Gh-i46187Gh	10.04	3.25	2.02	1.23
	IMmeq-FB-19	III	11	25	i07163Gh-i56975Gb	26	5	i08062Gh-i33827Gh	8.78	0.82	0.17	0.65
	IMmeq-FB-20	III	13	0	i17985Gh-i12984Gh	26	15	i25512Gh-i07941Gh	9.34	2.78	0.84	1.94
	IMmeq-FB-21	III	8	30	i54149Gb-i00217Gh	26	20	i37251Gh-i23249Gh	10.28	3.29	1.24	2.05
	IMmeq-FB-22	III	9	75	i05825Gh-i14639Gh	26	35	i08565Gh-i36067Gh	8.52	2.64	1.48	1.17

BN	IMmeq-BN-1	III	5	40	i08988Gh-i45534Gh	6	40	i06505Gh-i23722Gh	9.98	1.26	0.73	0.53
	IMmeq-BN-2	III	7	10	i24917Gh-i26814Gh	7	40	i34772Gh-i43291Gh	9.72	3.04	1.76	1.28
	IMmeq-BN-3	III	8	25	i26219Gh-i32773Gh	8	50	i01126Gh-i04719Gh	8.57	2.34	1.56	0.78
	IMmeq-BN-4	III	9	10	i05758Gh-i19700Gh	9	70	i00393Gh-i04801Gh	9.37	3.62	2.51	1.11
	IMmeq-BN-5	II	3	5	**i34758Gh-i49377Gh**	10	15	i25267Gh-i30274Gh	8.76	3.01	1.99	1.01
	IMmeq-BN-6	III	2	25	i02761Gh-i02712Gh	11	5	i33855Gh-i07729Gh	8.58	2.31	1.63	0.68
	IMmeq-BN-7	III	3	80	i43226Gh-i45963Gh	11	5	i33855Gh-i07729Gh	10.32	3.64	2.43	1.22
	IMmeq-BN-8	III	5	35	i16543Gh-i22374Gh	11	5	i33855Gh-i07729Gh	9.89	3.43	2.22	1.21
	IMmeq-BN-9	III	6	10	i06061Gh-i05824Gh	11	5	i33855Gh-i07729Gh	9.03	2.84	1.78	1.06
	IMmeq-BN-10	III	7	60	i14398Gh-i01824Gh	11	5	i33855Gh-i07729Gh	8.80	1.88	1.65	0.22
	IMmeq-BN-11	II	9	45	**i46552Gh-i24387Gh**	11	5	i33855Gh-i07729Gh	8.90	2.88	1.69	1.19
	IMmeq-BN-12	III	10	15	i25267Gh-i30274Gh	11	5	i33855Gh-i07729Gh	9.68	3.12	1.87	1.24
	IMmeq-BN-13	III	11	5	i33855Gh-i07729Gh	11	10	i47563Gh-i01036Gh	9.14	3.42	1.83	1.58
	IMmeq-BN-14	III	13	0	i17985Gh-i12984Gh	14	20	i15345Gh-i18849Gh	8.99	3.95	2.95	0.99
	IMmeq-BN-15	III	14	15	i15343Gh-i31037Gh	14	20	i15345Gh-i18849Gh	9.51	0.69	0.69	0.00
	IMmeq-BN-16	III	11	5	i33855Gh-i07729Gh	14	85	i05035Gh-i22015Gh	8.80	3.06	1.98	1.08
	IMmeq-BN-17	III	6	10	i06061Gh-i05824Gh	16	35	i01640Gh-i00384Gh	9.30	2.31	1.96	0.35
	IMmeq-BN-18	III	11	5	i33855Gh-i07729Gh	16	65	i54704Gb-i01693Gh	9.28	3.41	2.36	1.05
	IMmeq-BN-19	II	1	25	i14664Gh-i02994Gh	18	15	**i31442Gh-i13146Gh**	8.58	2.17	1.55	0.61
	IMmeq-BN-20	III	14	40	i22707Gh-i38937Gh	18	40	i26970Gh-i39369Gh	8.75	3.14	1.39	1.76
	IMmeq-BN-21	III	11	5	i33855Gh-i07729Gh	18	45	i39369Gh-i13456Gh	9.77	3.34	1.90	1.43
	IMmeq-BN-22	III	11	5	i33855Gh-i07729Gh	20	5	i00478Gh-i11539Gh	9.17	3.32	2.21	1.10
	IMmeq-BN-23	III	9	70	i00393Gh-i04801Gh	20	15	i24747Gh-i11698Gh	11.24	3.16	2.35	0.81
	IMmeq-BN-24	III	13	40	i46668Gh-i00187Gh	20	55	i12251Gh-i11715Gh	8.74	2.96	2.17	0.78
	IMmeq-BN-25	III	13	0	i17985Gh-i12984Gh	22	45	i44682Gh-i25111Gh	9.85	3.66	2.94	0.72
	IMmeq-BN-26	III	11	5	i33855Gh-i07729Gh	24	45	i26213Gh-i00339Gh	10.56	2.74	2.13	0.60

	IMmeq-BN-27	II	3	5	**i34758Gh-i49377Gh**	24	55	i14999Gh-i14993Gh	10.88	4.07	3.00	1.06
	IMmeq-BN-28	III	9	70	i00393Gh-i04801Gh	24	55	i14999Gh-i14993Gh	8.88	2.60	1.64	0.96
	IMmeq-BN-29	III	14	55	i23629Gh-i15587Gh	24	55	i14999Gh-i14993Gh	10.25	2.68	2.02	0.66
	IMmeq-BN-30	III	3	40	i00971Gh-i46613Gh	25	20	i41210Gh-i42629Gh	10.42	3.17	1.53	1.63
	IMmeq-BN-31	III	14	60	i26838Gh-i01129Gh	25	30	i11464Gh-i46788Gh	9.11	3.07	2.44	0.63
	IMmeq-BN-32	III	9	40	i10438Gh-i08573Gh	26	45	i22171Gh-i00945Gh	8.59	1.90	1.39	0.51
	IMmeq-BN-33	III	26	15	i25512Gh-i07941Gh	26	45	i22171Gh-i00945Gh	9.84	2.92	2.18	0.74
BW	IMmeq-BW-1	III	9	15	i40336Gh-i07864Gh	10	0	i43940Gh-i25267Gh	8.78	2.50	1.20	1.30
	IMmeq-BW-2	III	9	90	i13502Gh-i25039Gh	13	40	i46668Gh-i00187Gh	10.59	2.86	1.35	1.51
	IMmeq-BW-3	III	13	55	i21560Gh-i46408Gh	18	80	i43889Gh-i25079Gh	9.56	2.85	1.27	1.58
	IMmeq-BW-4	III	18	80	i43889Gh-i25079Gh	18	110	i45991Gh-i13081Gh	9.75	3.00	1.24	1.76
LP	IMmeq-LP-1	III	4	15	i41085Gh-i38159Gh	6	10	i06061Gh-i05824Gh	8.67	2.56	0.85	1.70
	IMmeq-LP-2	III	4	15	i41085Gh-i38159Gh	9	75	i05825Gh-i14639Gh	10.00	3.38	0.95	2.43
	IMmeq-LP-3	III	3	90	i31859Gh-i42939Gh	10	0	i43940Gh-i25267Gh	8.83	3.20	0.53	2.67
	IMmeq-LP-4	III	9	5	i25689Gh-i17373Gh	10	0	i43940Gh-i25267Gh	10.05	3.51	0.67	2.84
	IMmeq-LP-5	III	5	40	i08988Gh-i45534Gh	12	0	i40974Gh-i48211Gh	10.03	0.46	0.15	0.31
	IMmeq-LP-6	III	9	75	i05825Gh-i14639Gh	12	0	i40974Gh-i48211Gh	8.96	3.33	0.94	2.39
	IMmeq-LP-7	III	8	5	i31145Gh-i40270Gh	17	55	i03522Gh-i03688Gh	10.10	2.53	1.04	1.49
	IMmeq-LP-8	III	17	0	i14907Gh-i14878Gh	18	0	i13766Gh-i13754Gh	8.57	2.52	0.97	1.55
	IMmeq-LP-9	III	4	15	i41085Gh-i38159Gh	18	15	i31442Gh-i13146Gh	9.57	1.04	0.72	0.32
	IMmeq-LP-10	III	14	20	i15345Gh-i18849Gh	20	10	i39228Gh-i34769Gh	8.67	2.73	1.31	1.42
	IMmeq-LP-11	III	3	75	i43226Gh-i45963Gh	20	80	i11915Gh-i11478Gh	8.60	3.07	0.72	2.35
	IMmeq-LP-12	III	8	55	i01126Gh-i04719Gh	21	45	i07446Gh-i16079Gh	9.97	0.47	0.32	0.16
	IMmeq-LP-13	III	1	10	i53010Gb-i21390Gh	22	20	i12927Gh-i12929Gh	9.29	2.46	1.00	1.46
	IMmeq-LP-14	III	2	60	i02432Gh-i14623Gh	22	20	i12927Gh-i12929Gh	11.46	3.48	1.36	2.12
	IMmeq-LP-15	III	5	65	i37142Gh-i48326Gh	22	20	i12927Gh-i12929Gh	9.44	2.61	1.03	1.58

	IMmeq-LP-16	III	5	45	i29825Gh-i01144Gh	24	20	i36485Gh-i41754Gh	8.80	1.92	0.61	1.30
	IMmeq-LP-17	III	3	90	i31859Gh-i42939Gh	24	40	i48423Gh-i43942Gh	9.26	2.53	0.52	2.01
	IMmeq-LP-18	III	9	5	i25689Gh-i17373Gh	24	40	i48423Gh-i43942Gh	8.68	2.45	0.56	1.89
	IMmeq-LP-19	III	14	85	i05035Gh-i22015Gh	25	40	i22495Gh-i55440Gb	8.84	2.33	1.09	1.24
	IMmeq-LP-20	II	20	5	**i00478Gh-i11539Gh**	26	5	i08062Gh-i33827Gh	10.05	3.34	1.26	2.07
	IMmeq-LP-21	III	5	20	i35761Gh-i09052Gh	26	20	i37251Gh-i23249Gh	12.15	2.75	0.89	1.86
SY	IMmeq-SY-1	III	3	75	i43226Gh-i45963Gh	3	85	i31859Gh-i42939Gh	10.60	0.92	0.92	0.00
	IMmeq-SY-2	III	1	10	i53010Gb-i21390Gh	6	40	i06505Gh-i23722Gh	10.16	3.73	3.07	0.66
	IMmeq-SY-3	III	7	15	i46540Gh-i01765Gh	7	40	i34772Gh-i43291Gh	9.31	2.62	2.37	0.25
	IMmeq-SY-4	III	2	95	i07717Gh-i09654Gh	8	5	i31145Gh-i40270Gh	11.74	4.21	2.87	1.34
	IMmeq-SY-5	III	4	15	i41085Gh-i38159Gh	8	30	i54149Gb-i00217Gh	9.15	2.11	1.79	0.32
	IMmeq-SY-6	III	1	5	i21210Gh-i35065Gh	8	50	i01126Gh-i04719Gh	9.95	3.60	2.83	0.78
	IMmeq-SY-7	III	8	10	i24295Gh-i00234Gh	8	50	i01126Gh-i04719Gh	8.64	2.61	1.15	1.46
	IMmeq-SY-8	III	5	65	i37142Gh-i48326Gh	9	40	i10438Gh-i08573Gh	8.95	3.18	1.48	1.70
	IMmeq-SY-9	III	8	5	i31145Gh-i40270Gh	13	10	i45163Gh-i30934Gh	9.14	2.85	1.47	1.38
	IMmeq-SY-10	III	9	70	i00393Gh-i04801Gh	13	10	i45163Gh-i30934Gh	9.82	3.48	3.06	0.43
	IMmeq-SY-11	III	7	65	i14398Gh-i01824Gh	13	15	i30934Gh-i18151Gh	8.87	2.19	1.72	0.47
	IMmeq-SY-12	III	1	10	i53010Gb-i21390Gh	13	40	i46668Gh-i00187Gh	8.90	3.16	2.86	0.30
	IMmeq-SY-13	III	2	20	i17680Gh-i02755Gh	13	40	i46668Gh-i00187Gh	9.10	3.18	2.82	0.36
	IMmeq-SY-14	III	13	10	i45163Gh-i30934Gh	14	20	i15345Gh-i18849Gh	8.62	3.31	2.71	0.59
	IMmeq-SY-15	III	10	65	i22625Gh-i22107Gh	14	55	i23629Gh-i15587Gh	10.14	3.39	2.74	0.65
	IMmeq-SY-16	III	9	10	i05758Gh-i19700Gh	14	60	i26838Gh-i01129Gh	12.77	0.37	0.26	0.11
	IMmeq-SY-17	III	5	40	i08988Gh-i45534Gh	17	45	i00956Gh-i42547Gh	11.01	1.15	0.56	0.59
	IMmeq-SY-18	III	15	0	i02955Gh-i02314Gh	17	50	i14844Gh-i03522Gh	10.09	3.23	2.08	1.15
	IMmeq-SY-19	III	7	20	i01696Gh-i57601Gb	18	0	i13766Gh-i13754Gh	9.46	2.95	1.77	1.18
	IMmeq-SY-20	III	1	10	i53010Gb-i21390Gh	18	10	i13145Gh-i29829Gh	8.60	3.08	2.71	0.37

	IMmeq-SY-21	III	17	35	i03218Gh-i03216Gh	18	40	i26970Gh-i39369Gh	14.72	0.76	0.33	0.43
	IMmeq-SY-22	III	18	0	i13766Gh-i13754Gh	18	65	i41872Gh-i45940Gh	9.23	2.43	1.44	0.99
	IMmeq-SY-23	III	5	40	i08988Gh-i45534Gh	20	10	i39228Gh-i34769Gh	13.27	0.33	0.09	0.24
	IMmeq-SY-24	III	21	50	i35971Gh-i47631Gh	21	60	i22642Gh-i41613Gh	12.83	1.47	1.47	0.00
	IMmeq-SY-25	III	3	70	i22169Gh-i33635Gh	22	25	i12539Gh-i12581Gh	9.17	2.37	1.96	0.41
	IMmeq-SY-26	III	7	65	i14398Gh-i01824Gh	22	45	i44682Gh-i25111Gh	9.11	2.13	1.78	0.35
	IMmeq-SY-27	III	5	65	i37142Gh-i48326Gh	22	50	i44682Gh-i25111Gh	9.25	3.09	2.06	1.03
	IMmeq-SY-28	III	16	40	i45950Gh-i36953Gh	24	15	i04568Gh-i25656Gh	9.33	3.11	2.21	0.90
	IMmeq-SY-29	III	8	50	i01126Gh-i04719Gh	24	60	i03705Gh-i33113Gh	8.76	2.40	1.39	1.01
	IMmeq-SY-30	III	12	15	i40974Gh-i48211Gh	25	25	i27022Gh-i11449Gh	9.19	3.02	2.26	0.75
	IMmeq-SY-31	III	24	20	i36485Gh-i41754Gh	25	45	i21894Gh-i19984Gh	10.36	3.50	3.08	0.42
	IMmeq-SY-32	III	16	55	i21384Gh-i44137Gh	26	45	i22171Gh-i00945Gh	8.54	2.60	1.57	1.03
	IMmeq-SY-33	III	20	25	i25398Gh-i11734Gh	26	45	i22171Gh-i00945Gh	8.54	2.86	2.36	0.50
	IMmeq-SY-34	III	21	60	i22642Gh-i41613Gh	26	45	i22171Gh-i00945Gh	9.33	3.11	1.59	1.52
LY	IMmeq-LY-1	III	3	75	i43226Gh-i45963Gh	3	85	i31859Gh-i42939Gh	12.77	0.99	0.99	0.00
	IMmeq-LY-2	III	1	10	i53010Gb-i21390Gh	6	40	i06505Gh-i23722Gh	9.06	3.18	2.73	0.45
	IMmeq-LY-3	III	2	60	i02432Gh-i14623Gh	6	40	i06505Gh-i23722Gh	9.20	3.23	2.78	0.45
	IMmeq-LY-4	III	5	40	i08988Gh-i45534Gh	6	40	i06505Gh-i23722Gh	10.34	1.76	1.36	0.40
	IMmeq-LY-5	III	2	95	i07717Gh-i09654Gh	8	5	i31145Gh-i40270Gh	9.19	3.45	2.39	1.06
	IMmeq-LY-6	III	1	5	i21210Gh-i35065Gh	8	50	i01126Gh-i04719Gh	9.60	3.42	2.65	0.76
	IMmeq-LY-7	III	8	15	i04570Gh-i04506Gh	9	10	i05758Gh-i19700Gh	9.89	0.07	0.03	0.04
	IMmeq-LY-8	III	5	65	i37142Gh-i48326Gh	9	40	i10438Gh-i08573Gh	9.32	3.22	1.26	1.96
	IMmeq-LY-9	III	8	10	i24295Gh-i00234Gh	12	0	i40974Gh-i48211Gh	8.80	3.47	1.89	1.59
	IMmeq-LY-10	III	11	40	i43181Gh-i16165Gh	12	0	i40974Gh-i48211Gh	8.64	3.33	2.35	0.98
	IMmeq-LY-11	III	9	70	i00393Gh-i04801Gh	13	10	i45163Gh-i30934Gh	9.43	3.22	2.77	0.45
	IMmeq-LY-12	III	7	65	i14398Gh-i01824Gh	13	15	i30934Gh-i18151Gh	9.33	2.06	1.63	0.43

	IMmeq-LY-13	III	9	70	i00393Gh-i04801Gh	14	85	i05035Gh-i22015Gh	9.96	3.29	2.70	0.59
	IMmeq-LY-14	III	16	25	i29957Gh-i01669Gh	17	40	i03508Gh-i18575Gh	9.83	2.60	2.22	0.37
	IMmeq-LY-15	III	5	40	i08988Gh-i45534Gh	17	45	i00956Gh-i42547Gh	13.35	1.45	0.69	0.76
	IMmeq-LY-16	III	15	0	i02955Gh-i02314Gh	17	50	i14844Gh-i03522Gh	10.45	3.17	2.12	1.04
	IMmeq-LY-17	III	17	55	i03522Gh-i03688Gh	18	0	i13766Gh-i13754Gh	10.80	2.47	1.01	1.46
	IMmeq-LY-18	III	1	10	i53010Gb-i21390Gh	18	10	i13145Gh-i29829Gh	8.83	3.02	2.67	0.35
	IMmeq-LY-19	III	7	5	i37773Gh-i30640Gh	18	50	i13451Gh-i38577Gh	8.69	2.63	1.30	1.33
	IMmeq-LY-20	III	7	65	i14398Gh-i01824Gh	20	10	i39228Gh-i34769Gh	9.20	2.50	1.56	0.94
	IMmeq-LY-21	III	21	50	i35971Gh-i47631Gh	21	60	i22642Gh-i41613Gh	13.01	1.53	1.53	0.00
	IMmeq-LY-22	III	15	20	i29719Gh-i64628Gm	22	20	i12927Gh-i12929Gh	8.85	2.91	2.07	0.84
	IMmeq-LY-23	III	3	70	i22169Gh-i33635Gh	22	25	i12539Gh-i12581Gh	8.62	2.15	1.70	0.45
	IMmeq-LY-24	III	7	65	i14398Gh-i01824Gh	22	45	i44682Gh-i25111Gh	10.88	2.58	2.05	0.54
	IMmeq-LY-25	III	5	65	i37142Gh-i48326Gh	22	50	i44682Gh-i25111Gh	9.49	2.95	1.99	0.95
	IMmeq-LY-26	III	21	50	i35971Gh-i47631Gh	24	10	i04567Gh-i15176Gh	9.07	3.54	2.67	0.87
	IMmeq-LY-27	III	22	45	i44682Gh-i25111Gh	24	45	i26213Gh-i00339Gh	9.11	2.58	2.23	0.36
	IMmeq-LY-28	III	3	65	i49177Gh-i39896Gh	25	25	i27022Gh-i11449Gh	10.00	3.29	2.39	0.90
	IMmeq-LY-29	III	12	15	i40974Gh-i48211Gh	25	25	i27022Gh-i11449Gh	9.06	3.14	2.30	0.84
	IMmeq-LY-30	II	13	0	**i17985Gh-i12984Gh**	25	45	i21894Gh-i19984Gh	8.56	3.45	1.08	2.37
	IMmeq-LY-31	III	24	20	i36485Gh-i41754Gh	25	45	i21894Gh-i19984Gh	8.81	2.77	2.48	0.29
	IMmeq-LY-32	III	20	25	i25398Gh-i11734Gh	26	45	i22171Gh-i00945Gh	9.61	3.16	2.69	0.47
	IMmeq-LY-33	III	21	60	i22642Gh-i41613Gh	26	45	i22171Gh-i00945Gh	8.87	3.20	1.66	1.54
					**HSBCF_1MPHs**							
FB	B_1Mmeq-FB-1	III	7	25	i33174Gh-i01631Gh	13	55	i21560Gh-i46408Gh	6.90	2.59	2.51	0.08
	B_1Mmeq-FB-2	III	11	15	i01036Gh-i07468Gh	14	25	i15375Gh-i05040Gh	7.11	2.87	1.41	1.46
	B_1Mmeq-FB-3	III	14	30	i35260Gh-i35101Gh	15	15	i02469Gh-i46030Gh	7.18	2.85	1.57	1.28
	B_1Mmeq-FB-4	III	14	30	i35260Gh-i35101Gh	20	40	i37554Gh-i47006Gh	7.24	3.00	1.56	1.44

	B_1Mmeq-FB-5	III	4	5	i26515Gh-i43091Gh	24	55	i14999Gh-i14993Gh	6.20	2.48	1.48	0.99
	B_1Mmeq-FB-6	III	24	55	i14999Gh-i14993Gh	26	10	i33827Gh-i25834Gh	6.62	2.52	0.29	2.22
	B_1Mmeq-FB-7	III	6	35	i06036Gh-i06037Gh	26	15	i25512Gh-i07941Gh	6.51	2.72	2.16	0.56
BN	B_1Mmeq-BN-1	III	4	30	i10502Gh-i36496Gh	9	0	i40221Gh-i15598Gh	6.32	2.81	2.15	0.67
	B_1Mmeq-BN-2	III	7	0	i32739Gh-i37773Gh	9	45	i46552Gh-i24387Gh	6.41	2.47	2.20	0.27
	B_1Mmeq-BN-3	III	13	25	i13079Gh-i36296Gh	13	65	i29310Gh-i00241Gh	6.28	2.68	2.21	0.47
	B_1Mmeq-BN-4	III	8	30	i54149Gb-i00217Gh	14	15	i15343Gh-i31037Gh	6.15	2.40	2.17	0.23
	B_1Mmeq-BN-5	III	11	15	i01036Gh-i07468Gh	14	30	i35260Gh-i35101Gh	7.18	2.79	2.18	0.61
	B_1Mmeq-BN-6	III	5	55	i09147Gh-i42098Gh	19	15	i28797Gh-i09073Gh	6.28	2.05	2.04	0.01
	B_1Mmeq-BN-7	III	8	0	i63682Gm-i37825Gh	20	25	i25398Gh-i11734Gh	6.39	2.66	2.13	0.53
	B_1Mmeq-BN-8	III	4	30	i10502Gh-i36496Gh	20	40	i37554Gh-i47006Gh	9.05	3.71	2.53	1.18
	B_1Mmeq-BN-9	III	14	30	i35260Gh-i35101Gh	20	40	i37554Gh-i47006Gh	6.79	2.88	2.21	0.67
	B_1Mmeq-BN-10	II	4	30	i10502Gh-i36496Gh	21	60	**i22642Gh-i41613Gh**	8.34	3.42	2.28	1.15
	B_1Mmeq-BN-11	III	8	0	i63682Gm-i37825Gh	24	20	i36485Gh-i41754Gh	6.27	2.66	1.98	0.68
BW	B_1Mmeq-BW-1	III	6	15	i19214Gh-i31843Gh	6	35	i06036Gh-i06037Gh	6.37	2.24	0.55	1.69
	B_1Mmeq-BW-2	III	3	90	i31859Gh-i42939Gh	16	40	i45950Gh-i36953Gh	6.28	1.92	0.46	1.47
	B_1Mmeq-BW-3	III	7	65	i14398Gh-i01824Gh	18	35	i32883Gh-i13851Gh	6.18	2.35	0.65	1.71
	B_1Mmeq-BW-4	III	17	0	i14907Gh-i14878Gh	22	40	i12810Gh-i17697Gh	6.24	2.24	1.18	1.06
LP	B_1Mmeq-LP-1	III	4	30	i10502Gh-i36496Gh	9	0	i40221Gh-i15598Gh	6.32	2.81	2.15	0.67
	B_1Mmeq-LP-2	III	7	0	i32739Gh-i37773Gh	9	45	i46552Gh-i24387Gh	6.41	2.47	2.20	0.27
	B_1Mmeq-LP-3	III	13	25	i13079Gh-i36296Gh	13	65	i29310Gh-i00241Gh	6.28	2.68	2.21	0.47
	B_1Mmeq-LP-4	III	8	30	i54149Gb-i00217Gh	14	15	i15343Gh-i31037Gh	6.15	2.40	2.17	0.23
	B_1Mmeq-LP-5	III	11	15	i01036Gh-i07468Gh	14	30	i35260Gh-i35101Gh	7.18	2.79	2.18	0.61
	B_1Mmeq-LP-6	II	5	55	**i09147Gh-i42098Gh**	19	15	i28797Gh-i09073Gh	6.28	2.05	2.04	0.01
	B_1Mmeq-LP-7	III	8	0	i63682Gm-i37825Gh	20	25	i25398Gh-i11734Gh	6.39	2.66	2.13	0.53
	B_1Mmeq-LP-8	III	4	30	i10502Gh-i36496Gh	20	40	i37554Gh-i47006Gh	9.05	3.71	2.53	1.18

	B_1Mmeq-LP-9	III	14	30	i35260Gh-i35101Gh	20	40	i37554Gh-i47006Gh	6.79	2.88	2.21	0.67
	B_1Mmeq-LP-10	III	4	30	i10502Gh-i36496Gh	21	60	i22642Gh-i41613Gh	8.34	3.42	2.28	1.15
	B_1Mmeq-LP-11	III	8	0	i63682Gm-i37825Gh	24	20	i36485Gh-i41754Gh	6.27	2.66	1.98	0.68
SY	B_1Mmeq-SY-1	III	2	70	i00890Gh-i02271Gh	5	55	i09147Gh-i42098Gh	7.00	2.28	0.57	1.71
	B_1Mmeq-SY-2	III	2	75	i02276Gh-i43470Gh	6	40	i06505Gh-i23722Gh	6.53	2.35	0.75	1.60
	B_1Mmeq-SY-3	III	6	35	i06036Gh-i06037Gh	7	10	i24917Gh-i26814Gh	6.80	2.51	1.38	1.13
	B_1Mmeq-SY-4	III	6	35	i06036Gh-i06037Gh	8	10	i24295Gh-i00234Gh	7.50	2.82	1.51	1.31
	B_1Mmeq-SY-5	III	3	75	i43226Gh-i45963Gh	9	5	i25689Gh-i17373Gh	8.32	1.82	1.48	0.34
	B_1Mmeq-SY-6	II	14	45	i27231Gh-i36385Gh	14	50	**i34963Gh-i44045Gh**	7.38	0.14	0.14	0.00
	B_1Mmeq-SY-7	III	7	0	i32739Gh-i37773Gh	16	70	i54704Gb-i01693Gh	7.22	2.49	1.43	1.06
	B_1Mmeq-SY-8	III	3	95	i42939Gh-i05394Gh	18	40	i26970Gh-i39369Gh	6.07	2.09	1.47	0.61
	B_1Mmeq-SY-9	III	14	40	i22707Gh-i38937Gh	19	15	i28797Gh-i09073Gh	6.05	1.85	0.72	1.13
	B_1Mmeq-SY-10	III	3	40	i00971Gh-i46613Gh	19	35	i50235Gb-i52709Gb	6.05	2.12	1.11	1.01
	B_1Mmeq-SY-11	III	6	40	i06505Gh-i23722Gh	20	10	i39228Gh-i34769Gh	6.80	2.51	0.99	1.52
	B_1Mmeq-SY-12	III	18	5	i13754Gh-i13145Gh	20	25	i25398Gh-i11734Gh	7.25	2.53	1.61	0.92
	B_1Mmeq-SY-13	III	20	10	i39228Gh-i34769Gh	20	45	i17500Gh-i47439Gh	6.95	1.84	0.75	1.08
	B_1Mmeq-SY-14	III	9	0	i40221Gh-i15598Gh	21	65	i22642Gh-i41613Gh	7.00	2.73	1.85	0.88
	B_1Mmeq-SY-15	III	8	15	i04570Gh-i04506Gh	22	50	i44682Gh-i25111Gh	6.02	2.19	0.70	1.49
	B_1Mmeq-SY-16	III	13	60	i12964Gh-i29310Gh	24	55	i14999Gh-i14993Gh	6.49	2.37	1.46	0.91
	B_1Mmeq-SY-17	III	18	5	i13754Gh-i13145Gh	25	20	i41210Gh-i42629Gh	8.56	2.87	1.42	1.45
	B_1Mmeq-SY-18	III	19	40	i08786Gh-i00558Gh	25	35	i11465Gh-i39910Gh	7.13	2.03	1.63	0.40
	B_1Mmeq-SY-19	III	7	10	i24917Gh-i26814Gh	25	45	i21894Gh-i19984Gh	6.87	2.48	1.78	0.71
	B_1Mmeq-SY-20	III	5	30	i53001Gb-i08984Gh	26	0	i00879Gh-i08691Gh	7.11	2.73	1.84	0.89
	B_1Mmeq-SY-21	III	6	10	i06061Gh-i05824Gh	26	5	i08062Gh-i33827Gh	6.43	2.25	1.36	0.90
	B_1Mmeq-SY-22	III	14	25	i15375Gh-i05040Gh	26	15	i25512Gh-i07941Gh	6.49	2.50	1.85	0.65
	B_1Mmeq-SY-23	III	9	0	i40221Gh-i15598Gh	26	45	i22171Gh-i00945Gh	6.24	2.18	1.02	1.16

LY	B_1Mmeq-LY-1	III	2	70	i00890Gh-i02271Gh	5	55	i09147Gh-i42098Gh	7.30	2.63	0.77	1.86
	B_1Mmeq-LY-2	III	2	75	i02276Gh-i43470Gh	6	40	i06505Gh-i23722Gh	6.67	2.55	0.77	1.78
	B_1Mmeq-LY-3	III	6	35	i06036Gh-i06037Gh	7	15	i46540Gh-i01765Gh	7.31	2.81	1.73	1.08
	B_1Mmeq-LY-4	III	6	35	i06036Gh-i06037Gh	8	10	i24295Gh-i00234Gh	8.72	3.41	1.58	1.83
	B_1Mmeq-LY-5	III	4	25	i10502Gh-i36496Gh	8	25	i26219Gh-i32773Gh	7.57	3.04	1.02	2.02
	B_1Mmeq-LY-6	III	5	20	i35761Gh-i09052Gh	9	0	i40221Gh-i15598Gh	6.63	2.61	1.74	0.87
	B_1Mmeq-LY-7	III	3	75	i43226Gh-i45963Gh	9	5	i25689Gh-i17373Gh	8.05	2.00	1.36	0.64
	B_1Mmeq-LY-8	III	9	10	i05758Gh-i19700Gh	9	15	i40336Gh-i07864Gh	6.94	2.26	0.83	1.43
	B_1Mmeq-LY-9	III	4	25	i10502Gh-i36496Gh	14	15	i15343Gh-i31037Gh	6.65	2.44	0.44	2.00
	B_1Mmeq-LY-10	II	14	45	i27231Gh-i36385Gh	14	50	**i34963Gh-i44045Gh**	8.64	0.17	0.17	0.00
	B_1Mmeq-LY-11	III	2	5	i17680Gh-i02755Gh	15	30	i02459Gh-i02486Gh	6.06	1.94	1.77	0.17
	B_1Mmeq-LY-12	III	15	25	i18410Gh-i38002Gh	15	30	i02459Gh-i02486Gh	6.21	2.29	0.57	1.71
	B_1Mmeq-LY-13	III	11	15	i01036Gh-i07468Gh	15	45	i02315Gh-i07215Gh	6.16	2.18	1.66	0.51
	B_1Mmeq-LY-14	III	4	25	i10502Gh-i36496Gh	18	110	i45991Gh-i13081Gh	6.95	2.62	1.08	1.55
	B_1Mmeq-LY-15	III	14	40	i22707Gh-i38937Gh	19	15	i28797Gh-i09073Gh	6.94	2.32	0.70	1.62
	B_1Mmeq-LY-16	III	16	10	i13939Gh-i01279Gh	19	35	i50235Gb-i52709Gb	7.18	2.61	1.25	1.36
	B_1Mmeq-LY-17	III	2	85	i38985Gh-i30800Gh	20	0	i17414Gh-i17417Gh	6.47	1.79	1.28	0.51
	B_1Mmeq-LY-18	III	6	40	i06505Gh-i23722Gh	20	10	i39228Gh-i34769Gh	7.70	2.91	1.08	1.83
	B_1Mmeq-LY-19	III	18	5	i13754Gh-i13145Gh	20	25	i25398Gh-i11734Gh	6.70	2.64	1.27	1.37
	B_1Mmeq-LY-20	III	20	10	i39228Gh-i34769Gh	20	45	i17500Gh-i47439Gh	8.58	2.29	1.01	1.29
	B_1Mmeq-LY-21	III	9	0	i40221Gh-i15598Gh	21	65	i22642Gh-i41613Gh	7.38	3.04	1.67	1.37
	B_1Mmeq-LY-22	III	12	15	i40974Gh-i48211Gh	22	5	i00125Gh-i20168Gh	6.08	1.99	1.43	0.56
	B_1Mmeq-LY-23	III	4	15	i41085Gh-i38159Gh	22	20	i12927Gh-i12929Gh	6.88	2.75	1.81	0.94
	B_1Mmeq-LY-24	III	20	40	i37554Gh-i47006Gh	22	25	i12539Gh-i12581Gh	6.79	1.83	0.73	1.11
	B_1Mmeq-LY-25	III	4	25	i10502Gh-i36496Gh	25	20	i41210Gh-i42629Gh	6.05	2.42	0.68	1.75
	B_1Mmeq-LY-26	III	18	5	i13754Gh-i13145Gh	25	20	i41210Gh-i42629Gh	6.06	2.19	1.12	1.07

	B_1Mmeq-LY-27	III	19	40	i08786Gh-i00558Gh	25	35	i11465Gh-i39910Gh	8.83	2.59	1.80	0.80
	B_1Mmeq-LY-28	III	7	10	i24917Gh-i26814Gh	25	45	i21894Gh-i19984Gh	7.20	2.75	1.96	0.78
	B_1Mmeq-LY-29	III	1	10	i53010Gb-i21390Gh	25	50	i40453Gh-i46187Gh	6.44	2.45	1.19	1.26
	B_1Mmeq-LY-30	III	1	20	i47599Gh-i49208Gh	26	50	i16464Gh-i28856Gh	6.42	1.91	1.07	0.85
	B_1Mmeq-LY-31	III	2	80	i14841Gh-i38985Gh	26	50	i16464Gh-i28856Gh	6.36	0.52	0.52	0.00
	B_1Mmeq-LY-32	III	4	25	i10502Gh-i36496Gh	26	50	i16464Gh-i28856Gh	7.62	2.66	1.18	1.48
	B_1Mmeq-LY-33	III	5	55	i09147Gh-i42098Gh	26	50	i16464Gh-i28856Gh	7.38	1.18	1.02	0.16
	B_1Mmeq-LY-34	III	6	45	i23722Gh-i37862Gh	26	50	i16464Gh-i28856Gh	6.12	2.10	0.91	1.19
	B_1Mmeq-LY-35	III	13	25	i13079Gh-i36296Gh	26	50	i16464Gh-i28856Gh	7.43	2.60	1.31	1.30
	B_1Mmeq-LY-36	II	14	55	**i23629Gh-i15587Gh**	26	50	i16464Gh-i28856Gh	7.22	1.58	1.05	0.53
	B_1Mmeq-LY-37	III	15	5	i02306Gh-i02317Gh	26	50	i16464Gh-i28856Gh	6.94	2.56	1.23	1.33
	B_1Mmeq-LY-38	III	16	20	i24194Gh-i40100Gh	26	50	i16464Gh-i28856Gh	6.72	0.94	0.93	0.02
	B_1Mmeq-LY-39	III	17	55	i03522Gh-i03688Gh	26	50	i16464Gh-i28856Gh	7.16	1.57	1.08	0.49
	B_1Mmeq-LY-40	III	18	75	i13532Gh-i43889Gh	26	50	i16464Gh-i28856Gh	6.14	0.56	0.56	0.00
	B_1Mmeq-LY-41	III	20	10	i39228Gh-i34769Gh	26	50	i16464Gh-i28856Gh	6.84	1.25	0.92	0.33
	B_1Mmeq-LY-42	III	22	20	i12927Gh-i12929Gh	26	50	i16464Gh-i28856Gh	6.36	0.64	0.64	0.00
**MARBCF$_1$MPHs**												
FB	B_2Mmeq-FB-1	III	7	15	i46540Gh-i01765Gh	8	55	i01126Gh-i04719Gh	6.26	2.33	2.14	0.19
	B_2Mmeq-FB-2	III	8	30	i54149Gb-i00217Gh	17	25	i03512Gh-i22912Gh	6.01	2.21	1.03	1.18
	B_2Mmeq-FB-3	III	15	30	i02459Gh-i02486Gh	22	0	i00456Gh-i12928Gh	8.85	3.67	3.16	0.51
	B_2Mmeq-FB-4	III	6	30	i34827Gh-i15830Gh	26	30	i28715Gh-i21900Gh	6.22	1.83	1.18	0.65
	B_2Mmeq-FB-5	III	11	35	i40251Gh-i07190Gh	26	40	i36067Gh-i08578Gh	6.30	2.42	0.22	2.19
BN	B_2Mmeq-BN-1	III	7	15	i46540Gh-i01765Gh	8	55	i01126Gh-i04719Gh	6.13	2.45	2.31	0.14
	B_2Mmeq-BN-2	III	4	5	i26515Gh-i43091Gh	13	10	i45163Gh-i30934Gh	6.48	2.54	1.15	1.39
	B_2Mmeq-BN-3	III	9	15	i40336Gh-i07864Gh	17	0	i14907Gh-i14878Gh	7.23	2.87	2.75	0.12
	B_2Mmeq-BN-4	III	7	65	i14398Gh-i01824Gh	20	35	i40942Gh-i35292Gh	7.33	2.10	0.52	1.58

	B_2Mmeq-BN-5	III	19	5	i16591Gh-i08933Gh	21	65	i22642Gh-i41613Gh	8.20	3.06	2.84	0.22
	B_2Mmeq-BN-6	III	7	30	i38586Gh-i26820Gh	24	65	i03764Gh-i03721Gh	6.33	2.22	1.93	0.29
	B_2Mmeq-BN-7	III	5	25	i47720Gh-i37479Gh	26	15	i25512Gh-i07941Gh	6.37	2.33	2.28	0.05
BW	B_2Mmeq-BW-1	III	1	25	i14664Gh-i02994Gh	13	45	i13299Gh-i35111Gh	6.08	2.59	1.89	0.70
	B_2Mmeq-BW-2	III	15	40	i02315Gh-i07215Gh	26	35	i08565Gh-i36067Gh	6.65	2.61	0.66	1.95
LP	B_2Mmeq-LP-1	III	5	45	i29825Gh-i01144Gh	7	35	i42900Gh-i21721Gh	6.86	2.42	2.33	0.09
	B_2Mmeq-LP-2	III	8	25	i26219Gh-i32773Gh	18	10	i13145Gh-i29829Gh	8.66	3.44	2.79	0.65
	B_2Mmeq-LP-3	III	11	25	i07163Gh-i56975Gb	18	90	i64918Gm-i45991Gh	7.17	3.10	2.92	0.18
	B_2Mmeq-LP-4	III	9	40	i10438Gh-i08573Gh	20	5	i00478Gh-i11539Gh	7.17	3.10	2.62	0.48
	B_2Mmeq-LP-5	III	15	0	i02955Gh-i02314Gh	20	80	i11915Gh-i11478Gh	6.02	2.70	1.69	1.01
	B_2Mmeq-LP-6	III	16	20	i24194Gh-i40100Gh	22	40	i12810Gh-i17697Gh	6.29	2.26	2.13	0.14
	B_2Mmeq-LP-7	III	20	5	i00478Gh-i11539Gh	26	35	i08565Gh-i36067Gh	9.45	3.70	2.77	0.94
SY	B_2Mmeq-SY-1	III	1	0	i33646Gh-i40884Gh	3	105	i20709Gh-i23313Gh	9.10	4.07	0.49	3.59
	B_2Mmeq-SY-2	III	2	20	i17680Gh-i02755Gh	8	55	i01126Gh-i04719Gh	6.02	2.15	0.71	1.45
	B_2Mmeq-SY-3	III	1	0	i33646Gh-i40884Gh	9	5	i25689Gh-i17373Gh	9.10	4.08	0.39	3.69
	B_2Mmeq-SY-4	III	7	0	i32739Gh-i37773Gh	10	10	i43940Gh-i25267Gh	7.87	2.97	1.18	1.78
	B_2Mmeq-SY-5	III	8	25	i26219Gh-i32773Gh	11	0	i52789Gb-i07420Gh	6.13	2.78	2.71	0.08
	B_2Mmeq-SY-6	III	13	40	i46668Gh-i00187Gh	14	55	i23629Gh-i15587Gh	7.81	3.05	0.87	2.19
	B_2Mmeq-SY-7	III	11	30	i20872Gh-i40251Gh	14	85	i05035Gh-i22015Gh	6.27	2.13	1.06	1.07
	B_2Mmeq-SY-8	III	5	50	i16666Gh-i09095Gh	15	20	i29719Gh-i64628Gm	6.29	2.42	1.29	1.13
	B_2Mmeq-SY-9	III	2	0	i17680Gh-i02755Gh	16	55	i21384Gh-i44137Gh	6.40	2.62	1.45	1.18
	B_2Mmeq-SY-10	III	1	0	i33646Gh-i40884Gh	17	0	i14907Gh-i14878Gh	7.48	3.72	0.37	3.35
	B_2Mmeq-SY-11	III	11	35	i40251Gh-i07190Gh	18	115	i13081Gh-i13709Gh	6.57	2.43	0.90	1.53
	B_2Mmeq-SY-12	III	2	25	i02761Gh-i02712Gh	19	0	i09067Gh-i09082Gh	7.77	3.01	0.25	2.76
	B_2Mmeq-SY-13	III	11	35	i40251Gh-i07190Gh	20	35	i40942Gh-i35292Gh	6.26	2.72	1.45	1.27
	B_2Mmeq-SY-14	III	14	45	i27231Gh-i36385Gh	21	30	i47711Gh-i07558Gh	6.03	2.08	0.90	1.18

	B_2Mmeq-SY-15	III	7	60	i14398Gh-i01824Gh	21	60	i22642Gh-i41613Gh	6.59	2.72	1.25	1.47
	B_2Mmeq-SY-16	III	13	40	i46668Gh-i00187Gh	22	5	i00125Gh-i20168Gh	6.10	2.19	1.42	0.77
	B_2Mmeq-SY-17	III	1	35	i02298Gh-i42430Gh	22	20	i12927Gh-i12929Gh	6.02	2.36	1.64	0.73
	B_2Mmeq-SY-18	III	19	5	i16591Gh-i08933Gh	24	5	i04105Gh-i18808Gh	6.06	1.56	0.26	1.30
	B_2Mmeq-SY-19	II	21	20	**i07714Gh-i38909Gh**	24	50	i38401Gh-i04575Gh	6.85	1.75	1.40	0.35
	B_2Mmeq-SY-20	II	16	25	**i29957Gh-i01669Gh**	24	55	i14999Gh-i14993Gh	6.09	2.19	0.62	1.57
	B_2Mmeq-SY-21	III	12	10	i40974Gh-i48211Gh	24	60	i03705Gh-i33113Gh	6.21	2.09	1.46	0.63
	B_2Mmeq-SY-22	III	5	50	i16666Gh-i09095Gh	25	45	i21894Gh-i19984Gh	7.31	2.91	2.34	0.57
	B_2Mmeq-SY-23	III	7	30	i38586Gh-i26820Gh	25	60	i17145Gh-i10628Gh	6.58	2.40	1.29	1.11
	B_2Mmeq-SY-24	III	10	45	i26780Gh-i33011Gh	26	5	i08062Gh-i33827Gh	6.98	2.69	1.75	0.94
	B_2Mmeq-SY-25	III	17	50	i14844Gh-i03522Gh	26	5	i08062Gh-i33827Gh	6.08	2.37	1.70	0.67
	B_2Mmeq-SY-26	III	24	55	i14999Gh-i14993Gh	26	15	i25512Gh-i07941Gh	6.33	3.06	1.59	1.47
	B_2Mmeq-SY-27	III	19	35	i50235Gb-i52709Gb	26	20	i37251Gh-i23249Gh	6.11	2.57	1.07	1.50
LY	B_2Mmeq-LY-1	III	1	0	i33646Gh-i40884Gh	3	105	i20709Gh-i23313Gh	8.55	4.00	0.70	3.29
	B_2Mmeq-LY-2	III	4	15	i41085Gh-i38159Gh	6	5	i30129Gh-i06111Gh	6.11	2.61	2.51	0.10
	B_2Mmeq-LY-3	III	1	0	i33646Gh-i40884Gh	9	5	i25689Gh-i17373Gh	8.61	4.01	0.45	3.56
	B_2Mmeq-LY-4	III	7	0	i32739Gh-i37773Gh	10	10	i43940Gh-i25267Gh	7.02	2.85	1.34	1.51
	B_2Mmeq-LY-5	III	5	35	i16543Gh-i22374Gh	13	40	i46668Gh-i00187Gh	7.50	2.46	1.92	0.54
	B_2Mmeq-LY-6	III	13	40	i46668Gh-i00187Gh	14	55	i23629Gh-i15587Gh	7.45	3.03	0.94	2.08
	B_2Mmeq-LY-7	III	1	0	i33646Gh-i40884Gh	17	0	i14907Gh-i14878Gh	7.61	3.77	0.43	3.34
	B_2Mmeq-LY-8	III	14	25	i15375Gh-i05040Gh	18	70	i49258Gh-i13532Gh	7.33	3.11	1.59	1.52
	B_2Mmeq-LY-9	III	11	0	i52789Gb-i07420Gh	18	115	i13081Gh-i13709Gh	6.57	2.59	1.12	1.47
	B_2Mmeq-LY-10	III	2	75	i02276Gh-i43470Gh	19	30	i16566Gh-i08941Gh	8.76	3.50	1.27	2.22
	B_2Mmeq-LY-11	III	7	60	i14398Gh-i01824Gh	21	40	i07558Gh-i07515Gh	6.09	2.23	0.56	1.68
	B_2Mmeq-LY-12	III	13	40	i46668Gh-i00187Gh	22	5	i00125Gh-i20168Gh	6.12	2.33	1.42	0.90
	B_2Mmeq-LY-13	III	1	35	i02298Gh-i42430Gh	22	20	i12927Gh-i12929Gh	6.20	2.57	1.60	0.97

B_2Mmeq-LY-14	III	14	55	i23629Gh-i15587Gh	24	50	i38401Gh-i04575Gh	6.01	2.36	0.49	1.87
B_2Mmeq-LY-15	III	21	65	i22642Gh-i41613Gh	24	55	i14999Gh-i14993Gh	7.13	2.99	0.32	2.66
B_2Mmeq-LY-16	III	7	30	i38586Gh-i26820Gh	25	60	i17145Gh-i10628Gh	7.16	2.80	1.06	1.74
B_2Mmeq-LY-17	III	10	45	i26780Gh-i33011Gh	26	5	i08062Gh-i33827Gh	7.16	2.90	1.83	1.07
B_2Mmeq-LY-18	III	17	50	i14844Gh-i03522Gh	26	5	i08062Gh-i33827Gh	6.20	2.53	1.78	0.75
B_2Mmeq-LY-19	III	24	55	i14999Gh-i14993Gh	26	15	i25512Gh-i07941Gh	7.28	3.61	2.17	1.45

[a] FB: number of fruit branches per plant; BN: number of bolls per plant; BW: boll weight; LP: lint percentage; SY: seed cotton yield; LY: lint yield

[b] Type of epistasis: (I) two loci with m-QTL, (II) one loci with m-QTL and the other loci without significant m-QTL and (III) two loci without significant m-QTL

[c] Chi and Chj represent the chromosome number of the loci being tested in the analysis

[d] Position of e-QTL located on chromosome: as cM distance from the top of each chromosome

[e] Flanking markers in bold are those flanking m-QTLs identified by ICIM in additional Table S7

[f] A LOD threshold was used for declaration of QTL based on 1000 permutations at as significance level of 0.01

[g] PV: the phenotypic variation that the total epistasis effect explained; PV(AA): the phenotypic variation that the main epistasis effect explained; PV (AAE): the phenotypic variation that the environmental interaction of the epistasis effect explained
